# Supplementary material for: Environmental variables drive plant species composition and distribution in the moist temperate forests of Northwestern Himalaya, Pakistan
Source: PLoS One. 2022 Feb 24;17(2):e0260687. doi: 10.1371/journal.pone.0260687 (PMC8870539; doi:10.1371/journal.pone.0260687)
Supplement: Attachment — Submitted filename: PONE-D-21-11989_R1_Editorial Comments.pdf [file pone.0260687.s005.pdf]

PLOS ONE

Environmental variables drive plant species composition and distribution in the moist temperate forests of Northwestern Himalaya, Pakistan

--Manuscript Draft--

**Manuscript Number:**	PONE-D-21-11989R1
**Article Type:**	Research Article
**Full Title:**	Environmental variables drive plant species composition and distribution in the moist temperate forests of Northwestern Himalaya, Pakistan
**Short Title:**	Plant communities' distribution in moist temperate forests
**Corresponding Author:**	Inayat Ur Rahman, PhD Scholar<br>Hazara University<br>Mansehra, PAKISTAN
**Keywords:**	environmental variables; plant species composition; plant species distribution; moist temperate forest; environmental gradients; hotspots of biodiversity; climate change; indicator species; multivariate approaches; Northwestern Himalaya; Plant community; environmental drivers
**Abstract:**	Questions – By assessing plant species composition and distribution in biodiversity hotspots influenced by environmental gradients, we greatly advance our understanding of the local plant community and how environmental factors are affecting these communities, which is a proxy for assessing how impacts of climate change can affect plant communities located in mountainous regions ("space-for-time" substitution). We evaluated plant species composition and distribution, and how and which environmental variables drive the plant communities in moist temperate forest of Northwestern Himalaya, Pakistan.<br>Location – Moist temperate zone of Manoor valley (District Mansehra, Khyber Pakhtunkhwa), Northwestern Himalayan belt of Pakistan.<br>Methods – During four consecutive years (2015-2018), we sampled 30 sampling sites, measuring 21 environmental variables and recording all plant species present in an altitudinal gradient range of 1932-3168 m.a.s.l. We used different multivariate analyses to identify potential plant communities, and to determine the relative importance of each environmental variable in the species composition and distribution. Finally, we also evaluated diversity patterns, by comparing diversity indices and beta diversity processes.<br>Results – We found that (i) the moist temperate zone in this region can be divided in four different major plant communities; (ii) each plant community has a specific set of environmental drivers; (iii) there is a significant variation in plant species composition between communities, in which six species contributed most to the plant composition dissimilarity; (iv) there is a significant difference of the four diversity indices between communities; and (v) plant community structure is twice more influenced by the spatial turnover of species than by the species loss.<br>Conclusions – Overall, we showed that altitudinal gradients offer an important range of different environmental variables, highlighting the existence of micro-climates that drive the structure and composition of plant species in each micro-region. Each plant community along the altitudinal gradient has a set of environmental drivers, which lead to the presence of indicator species in each micro-region.
**Order of Authors:**	Inayat Ur Rahman, PhD Scholar
	Robbie E Hart
	Farhana Ijaz
	Aftab Afzal
	Zafar Iqbal
	Eduardo Soares Calixto
	Elsayed Fathi Abd_Allah
	Abdulaziz A. Alqarawi

Powered by Editorial Manager® and ProduXion Manager® from Aries Systems Corporation

	Abeer Hashem
	Bandari Fahad Al-Arjani
	Rukhsana Kausar
	Sheikh Marifatul Haq
**Opposed Reviewers:**	
**Response to Reviewers:**	Revision notes<br>Reviewer's comments black font; author answers in blue font; responses are numbered (R#1, R#2, etc. for reference)<br>Reviewer(s)' Comments to Author:<br>Reviewer #1<br>General comments:<br>In this research paper, the authors studied influence of environmental variables in shaping plant species composition and their distribution in the moist temperate forests of Northwestern Himalaya, Pakistan. Covering 30 sampling sites, they measured 21 environmental variables for four consecutive years. Generated data is analysed using different multivariate analyses in order to identify potential plant communities and influence of environmental variable in species composition, distribution and diversity patterns. Overall the present research is well-designed and presents interesting results of multivariate analysis in order to understand how the existence of micro-climates drive the structure and composition of plant species in studied area.<br>R#1 – We really appreciate the positive words.<br><br>Major issue:<br>1.In Conclusions (line 46-48) author stated that overall, we showed that altitudinal gradients offer an important range of different environmental variables, highlighting the existence of micro-climates that drive the structure and composition of plant species in each micro-region. However there is no mention of elevation range of study area across the manuscript.<br>R#2 – We now added this information in the manuscript (L.14-15,93,237-246). The altitudinal range of our data sampling ranged from 1932 to 3168 m.a.s.l.<br><br>2.Kindly add some detailed information on sampling method such as: if quadrat method is opted during the study what was the size of these used quadrats. Furthermore it will be good for readers to understand the study if information regarding the number of sampling quadrates in each transect will be mentioned in the methodology section.<br>R#3 – Required information regarding the vegetation sampling has been added. "The surveyed stands (study area) were subdivided into 30 stands and three points randomly selected within each stand were sampled along 50 meters transect (total = 90 transects). The interval distance kept between the stands was 200 meters and 100 meters between transects." (L. 97-110)<br><br>3.A detailed table describing four communities (IHC; VIP; CPI; PCP) needs to be added highlighting species composition, environmental variables, elevation range, dominant families etc.<br>R#4 – We now added two new supplementary tables (Table S1 and S2) describing the communities. One table describes environmental variables and the other table the species composition for each plant community.<br><br>4.Page 9 line 177 author mentioned "A total of 244 plant species were recorded 177 in moist temperate forest of Manoor valley, Himalaya, Pakistan". Kindly add details of 244 plant species belonging to ……………genus and …….families respectively.<br>R#5 – As per suggestion, the required details has been added i.e., 194 genera and 74 families. (L. 220)<br><br>Other comments:<br>1.Kindly follow uniformity across the manuscript text to avoid confusion, such as spelling variations (in line 122 analyses and 123 analyzes) and such omissions needs to be checked throughout the manuscript.<br>R#6 – Done. Thank you. (L. 15, 139, 146, 219, 294)

2.Author has used the term Northwestern Himalaya (line 23); Himalaya (line 31): Northwestern Himalaya (line 102); Himalayan mountains (line 260) etc. thus it is suggested to use term Northwestern Himalaya to indicate study site throughout the manuscript.
R#7 – We changed to Northwestern Himalaya in the places that we are talking about the specific region of Himalayas. Other parts are related to the Himalayas in a general perspective and then we can change it. We appreciate your understanding. (L. 10, 12, 40, 71, 80, 92, 95 and onwards)

3.For wider acceptability of the manuscript it is suggested to discuss similar studies carried out in other parts of Himalaya in discussion section.
R#8 – We added more citations about studies conducted in the Himalayan mountains. We have now discussed it in detail. We now also added more citations about plant community studies in the Himalayas. (L. 327-329, 367-369, 376-382, 398-432)

Reviewer #2
The topic is timely, and such a kind of study, especially from the underrepresented areas, is essential and requires our attention. The authors have come up with clear questions and have analyzed them well.
R#9 – We really appreciate your positive words.

My main concern is with the methodology and discussion section; I see much basic information missing, especially concerning the data collection and study site; please look at each of the comments provided below. Discussion lacks proper explanation, key findings of different communities and predictor variables associated with them are interesting, but these points are not discussed well. Currently, the discussion is too general, but there is much potential to improve it. I also felt that your questions 2 and 4 explain the same points, so instead of keeping them as standalone objectives, it will be nice to combine them. In objective two results, all the four communities were found separately in clumps with apparent differences based on the environmental gradients, so I dint understand the need to again look into the variation of environmental variables among plant communities (iv). Lastly, instead of just reporting six species names, it will be good to have a table (primary or supplementary table), including all details of plant species in each community, their abundance, elevation range, etc.
R#10 – We now improved our manuscript and cordially ask you to check our responses for each question raised. Overall, we added more information in methods (L. 93, 97-110, 112-114, 121-125, 136-138, 140-142, 144-145, 148-156) and discussion (L. 327-329, 367-369, 376-382, 398-432) sections. Following reviewer's very important suggestion, two new supplementary tables with a summary of the environmental variables and the species composition based on the importance value were also added.

L39-40: ii) each plant community has a specific set of environmental drivers & L41-42 iv) most of the environmental variables were significantly different between communities. I feel both of these sentences highlight the same points; my advice would be to combine both and write them as a single point.
R#11 – Thank you for your suggestion. We agreed and combined accordingly. (L. 22, 84, 339)

L90-91- and L94: I am highlighting this point again; there is an overlap in your questions 2 and 4
R#12 – Please see R#11 for the answer about this question. (L. 22, 84, 339)

L106 What were the growing seasons? Please mention the seasons name
R#13 – We now added the months that represent the growing seasons in the area, which were from March to October. (L. 97)

L106 Was the same stand sampled for four years, meaning four times? Please note that many important information on sampling design is missing; for example, how were the individuals counted, those falling precisely on the line? Furthermore, was the line horizontal or vertical? Was it stratified random sampling? Also, what was the distance between the two lines transect?
R#14 – Yes. We repeated the stands over years, but not the same transects inside each stand. We now added more information about it the text. The surveyed stands

	(study area) were subdivided into 30 stands and three points randomly selected within each stand were sampled along 50 meters transect (total = 90 transects). The interval distance kept between the stands was 200 meters and 100 meters between transects. The individuals of plant species falling precisely on the line were noted. (L. 98-110)<br><br>L116 There is no clarity on how soil samples, Ph values were collected. Was it collected at a single location per stand, and what depth, slope, well-exposed sunlit areas or not?<br>Ph varies a lot, even at a short distance between north and south facing slopes, so it is better to provide all the sampling details.<br>R#15 – Basic details regarding the data collection of vegetation and soil sampling are now mentioned in Vegetation sampling (L. 98-110, 112-114), and Environmental gradients (L. 121-125, 136-138) respectively. A supplementary table S2 has also been included to show the variability of environmental gradients among the four plant communities.<br><br>L121 At what distance was the weather station located from those 30 stands? The environmental variable varies across the elevation, and authors have also mentioned the importance of microclimates, so I am curious to know how many weather stations were installed.<br>The overall sampling design will become more apparent if the authors explain how the line transects and weather stations are distributed along the elevation gradient.<br>R#16 – We used a small remote weather station and recorded the data at transect level and then averaged it to stand level. Required details regarding vegetation sampling using line transect are incorporated into the Vegetation sampling section (M & M).<br><br>Results:<br>I feel that apart from the given figures, it will be good to have a table where the species names, elevation range, abundance in each community are mentioned. A comprehensive table with all these details may give a clearer picture of your findings. You may decide how much information you would want to share in the main manuscript and supplementary information.<br>R#17 – Thank you for your comment. We now added two supplementary tables about plant species composition and environmental variables values in each community. Since we already have many tables and figures in the manuscript, and these other tables will be so long, we are adding them as supplementary tables (S1 and S2).<br><br>Fig 3: Legend need to be rewritten, the author has mentioned four communities' names and four colors corresponding to each community, but the link between each color and community is missing. Also, what do the alphabet and various numbers indicate on the left side of the graph?<br>R#18 – We now updated our legend. Numbers and letter are the stands.<br>“Figure 3. Clustering method using two way indicator species analysis (TWINSPAN) indicating four different plant communities. IHC (red triangle): Indigofera heterantha-Heracleum candicans-Cynodon dactylon, VIP (blue circle): Viburnum grandiflorum-Indigofera heterantha-Pinus wallichiana, CPI (green square): Cedrus deodara-Pinus wallichiana-Isodon rugosus and PCP (yellow diamond): Pinus wallichiana-Cedrus deodara- Parrotiopsis jacquemontiana. Letters associated to numbers at the end of each branch of the dendrogram represent the stands evaluated.<br><br>L209: What was the intention behind considering temperature and altitude both as the predictor variables? Because along the mountain gradient these variables are highly correlated.<br>R#19 – In this specific analysis, we did not consider any kind of multicollinearity. We plot all variables to see the overall relationship between them and communities. However, if you check the partial CCA and partitioning analyses, you will see that we removed the colinear variables. Therefore, in the first analysis (NMDS, PCA) we show the overall relationship of all variables with each community, while in the second analysis, we show which are the variables that most explain the patterns found in our study.<br><br>Discussion: In the result, section author has emphasized their findings of four distinct communities and the environmental variables associated with each one of them.

Powered by Editorial Manager® and ProduXion Manager® from Aries Systems Corporation

	However, in the discussion, the authors have failed to discuss their unique findings in detail. I feel that the discussion section is too general and poorly explained. There is a lot of scopes to improve the discussion considering the amount of analysis carried out. By highlighting just temperature and altitude (which is definitely important from the climate change aspect), you may lose other valuable details of your study.<br>R#20 – We totally agree. In discussion, we provided more details about how these other variables can influence plant community. We believe that discussing each variable and its relationship to the community will extend a lot the manuscript, turning it tiring for readers. In this context, we believe that the general patterns and discussion of the main variables should be the best approach. We appreciate your comment, and we cordially ask to check the lines mentioned, where we added information about the influence of other variables in the community structure. Some important edaphic variables like pH were considered and discussed accordingly. (L. 327-329, 367-369, 376-382, 398-432)<br><br>Reviewer #3<br>Comments on the manuscript entitled 'Environmental variables drive plant species composition and distribution in the moist temperate forests of Northwestern Himalaya, Pakistan'.<br><br>The basic data collection on vegetation, soil, etc. and analysis are completely lacking. Overall, the environmental observation of the study is very less (4 years, 2015-2018) to draw proper conclusion on impact of plant diversity.<br>Relation to calculating the vegetation diversity along with environmental parameters, in location specific data needs clarification / rectification.<br>R#21 – We agreed, basic information regarding the data collection of soil and vegetation sampling are now mentioned in detail as per suggestion. (L. 98-110, 112-114, 121-125, 136-138)<br><br>Further, the following points / comments need clarification / improvement.<br>Line no. 24-31: The abstract needs little improvement.<br>R#22 – Improved.<br><br>Line no. 32 & 100-104: The location can be provided with district / provinces / state, etc. in Pakistan.<br>R#23 – Required details provided in both the sections. (L. 11, 90-91)<br><br>Line no. 106: What are the growing seasons targeted? The time period of the study also be specified.<br>R#24 – We now added the months that represent the growing season in the area, which is from March to October. (L. 97)<br><br>Line no. 116-118: What is the deepness of soil sampling? Whether the soil has been collected for each stands / transect, please clarify.<br>R#25 – "Soil samples of 200-400grams were collected from three randomly selected transects (0-30cm depth) within each sampling stand of the studied vegetation area." For more details, please see section: Environmental gradients. (L. 121-125)<br><br>Line no. 119-121: The duration, intervals, etc. of data obtained from the handheld weather station and position / place of the weather station for the study are not clear.<br>R#26 – We used a small remote weather station and recorded the data at transect level and then averaged it to stand level. (L. 136-138)<br><br>Line no. 177: Details of 244 species recorded may be supplemented.<br>R#27 – Two new supplementary tables have been with a summary of the environmental variables and the species composition based on the importance value index.<br><br>Line no. 182-186: The community may be specified as Trees, Shrubs, Grass, etc. and can be analysed properly. For example, the presence of Indigofera is shrub and Cynodon is grass and the landscape is shrubland or grassland. On the other hand, the presence of Pinus and Cedrus are clearly representing tree communities. Whether the landscape is having patches of vegetation of trees / shrub / grass composition or uniform vegetation dominated by these group, need clarification.

Powered by Editorial Manager® and ProduXion Manager® from Aries Systems Corporation

	R#28 – As per suggestion, the required details have been mentioned following the communities. For instance, the IHC community was primarily found in the lower mountainous ranges (1932.3-2338.4 m.a.s.l), where the dominating flora was a combination of shrub and herb species owing to the existence of a substantial herbaceous layer of Cynodon dactylon, which carpeted the landscape alongside Indigofera heterantha patches……shrubby associates. (L. 237-246)<br>Line no. 190-194: Any specific observation on Cedrus, Pinus, etc. Because, we could not find the all vegetation in all aspects, especially the studied tree species.<br>R#29 – Please see R#28 for the answer about this question. (L. 237-246, 264-266)<br><br>Discussion: Need revision in view of answering the above questions.<br>R#30 – We provided more details about how the variables can influence plant community. We cordially ask to check the lines (L. 327-329, 367-369, 376-382, 398-432), where we added information about the influence of other variables in the community structure.<br><br>The manuscript holds excellent statistical analysis. Although, basic collection data on vegetation assessment and related environmental parameters are missing, and can be addressed.<br>We cordially appreciate your positive words, however, we have considered and addressed each of your suggestion accordingly.
**Additional Information:**	
**Question**	**Response**
**Financial Disclosure**<br><br>Enter a financial disclosure statement that describes the sources of funding for the work included in this submission. Review the submission guidelines for detailed requirements. View published research articles from *PLOS ONE* for specific examples.<br><br>This statement is required for submission and **will appear in the published article** if the submission is accepted. Please make sure it is accurate.	The author(s) received no specific funding for this work

**Unfunded studies**<br>Enter: *The author(s) received no specific funding for this work.*<br><br>**Funded studies**<br>Enter a statement with the following details:<br>• Initials of the authors who received each award<br>• Grant numbers awarded to each author<br>• The full name of each funder<br>• URL of each funder website<br>• Did the sponsors or funders play any role in the study design, data collection and analysis, decision to publish, or preparation of the manuscript?<br>• **NO** - Include this sentence at the end of your statement: *The funders had no role in study design, data collection and analysis, decision to publish, or preparation of the manuscript.*<br>• **YES** - Specify the role(s) played.<br><br>* typeset	
**Competing Interests**<br><br>Use the instructions below to enter a competing interest statement for this submission. On behalf of all authors, disclose any competing interests that could be perceived to bias this work—acknowledging all financial support and any other relevant financial or non-financial competing interests.<br><br>This statement is required for submission and **will appear in the published article** if the submission is accepted. Please make sure it is accurate and that any funding sources listed in your Funding Information later in the submission form are also declared in your Financial Disclosure statement.<br><br>View published research articles from *PLOS ONE* for specific examples.	The authors have declared that no competing interests exist.

**NO authors have competing interests**<br><br>Enter: *The authors have declared that no competing interests exist.*<br><br>**Authors with competing interests**<br><br>Enter competing interest details beginning with this statement:<br><br>*I have read the journal's policy and the authors of this manuscript have the following competing interests: [insert competing interests here]*<br><br>* typeset	
**Ethics Statement**<br><br>Enter an ethics statement for this submission. This statement is required if the study involved:<br><br>• Human participants<br>• Human specimens or tissue<br>• Vertebrate animals or cephalopods<br>• Vertebrate embryos or tissues<br>• Field research<br><br>Write "N/A" if the submission does not require an ethics statement.<br><br>General guidance is provided below. Consult the submission guidelines for detailed instructions. **Make sure that all information entered here is included in the Methods section of the manuscript.**	This study was approved by the Board of Study (BOS), Committee, Department of Botany and Advanced Study Research Board (ASRB) of Hazara University, Mansehra 21300, KP, Pakistan.

**Format for specific study types**<br><br>**Human Subject Research (involving human participants and/or tissue)**<br>• Give the name of the institutional review board or ethics committee that approved the study<br>• Include the approval number and/or a statement indicating approval of this research<br>• Indicate the form of consent obtained (written/oral) or the reason that consent was not obtained (e.g. the data were analyzed anonymously)<br><br>**Animal Research (involving vertebrate animals, embryos or tissues)**<br>• Provide the name of the Institutional Animal Care and Use Committee (IACUC) or other relevant ethics board that reviewed the study protocol, and indicate whether they approved this research or granted a formal waiver of ethical approval<br>• Include an approval number if one was obtained<br>• If the study involved *non-human primates*, add *additional details* about animal welfare and steps taken to ameliorate suffering<br>• If anesthesia, euthanasia, or any kind of animal sacrifice is part of the study, include briefly which substances and/or methods were applied<br><br>**Field Research**<br><br>Include the following details if this study involves the collection of plant, animal, or other materials from a natural setting:<br>• Field permit number<br>• Name of the institution or relevant body that granted permission	
**Data Availability**<br><br>Authors are required to make all data underlying the findings described fully available, without restriction, and from the time of publication. PLOS allows rare exceptions to address legal and ethical concerns. See the PLOS Data Policy and FAQ for detailed information.	Yes - all data are fully available without restriction

A Data Availability Statement describing where the data can be found is required at submission. Your answers to this question constitute the Data Availability Statement and **will be published in the article**, if accepted.<br><br>**Important:** Stating 'data available on request from the author' is not sufficient. If your data are only available upon request, select 'No' for the first question and explain your exceptional situation in the text box.<br><br>Do the authors confirm that all data underlying the findings described in their manuscript are fully available without restriction?	
**Describe where the data may be found in full sentences. If you are copying our sample text, replace any instances of XXX with the appropriate details.**<br><br>• If the data are **held or will be held in a public repository**, include URLs, accession numbers or DOIs. If this information will only be available after acceptance, indicate this by ticking the box below. For example: *All XXX files are available from the XXX database (accession number(s) XXX, XXX.)*.<br>• If the data are all contained **within the manuscript and/or Supporting Information files**, enter the following: *All relevant data are within the manuscript and its Supporting Information files.*<br>• If neither of these applies but you are able to provide **details of access elsewhere**, with or without limitations, please do so. For example:<br><br>*Data cannot be shared publicly because of [XXX]. Data are available from the XXX Institutional Data Access / Ethics Committee (contact via XXX) for researchers who meet the criteria for access to confidential data.*<br><br>*The data underlying the results presented in the study are available from (include the name of the third party*	All relevant data are within the manuscript and its Supporting Information files.

and contact information or URL).<br>• This text is appropriate if the data are owned by a third party and authors do not have permission to share the data.<br><br>* typeset	
Additional data availability information:	

THE MISSOURI BOTANICAL GARDEN
ST. LOUIS, MISSOURI, USA

Dear **Editor-in-Chief/Associate Editor**,

The manuscript entitled **“Environmental variables drive plant species composition and distribution in the moist temperate forests of Northwestern Himalaya, Pakistan”** has been revised in the light of editor and reviewers’ comments/suggestions for its consideration as a publication in the esteemed journal *PLOS ONE*.

We thank you and Reviewers for their constructive criticism and are pleased to resubmit a revised and improved version of our manuscript (ID # PONE-D-21-11989) entitled “”. We carefully responded to each comment in the revised version. In the response letter below, we have provided a detailed explanation of the changes made following the line numbers (see line numbers in track version).

We are looking forward to hearing from you!

With best regards,

INAYAT UR RAHMAN, PhD
William L. Brown Center,
Missouri Botanical Garden,
St. Louis, MO 63166-0299,
USA. Email:irahman@mobot.org

Manuscript
Click here to access/download;Manuscript;Manuscript_Clean copy.doc

Environmental variables drive plant species composition and distribution in the moist temperate forests of Northwestern Himalaya, Pakistan

Short running title: Plant communities' distribution in moist temperate forests

Inayat Ur Rahman[1,2*], Robbie E. Hart[2], Farhana Ijaz[1], Aftab Afzal[1], Zafar Iqbal[1], Eduardo S. Calixto[3,4], Elsayed Fathi Abd_Allah[5], Abdulaziz A. Alqarawi[5], Abeer Hashem[6,7], Al-Bandari Fahad Al-Arjani[6], Rukhsana Kausar[8], Shiekh Marifatul Haq[9]

1 Department of Botany, Hazara University Mansehra-21300, Khyber Pakhtunkhwa, Pakistan, **2** William L. Brown Center, Missouri Botanical Garden, 4344 Shaw Blvd, P.O. Box 299, St. Louis, MO 63110, USA, **3** Entomology and Nematology Department, University of Florida, Gainesville, FL, USA, **4** Department of Biology, University of Missouri St. Louis (UMSL), Saint Louis, MO, USA, **5** Department of Plant Production, College of Food and Agriculture Science, King Saud University, Riyadh 11451, Saudi Arabia, **6** Botany and Microbiology Department, College of Science, King Saud University, Riyadh 11451, Saudi Arabia, **7** Mycology and Plant Disease Survey Department, Plant Pathology Research Institute, Agriculture Research Center, Giza 12619, Egypt, **8** Department of Environmental Sciences, International Islamic University, Islamabad, Pakistan, **9** Department of Botany, University of Kashmir, Jammu & Kashmir, India

*Correspondence should be addressed to: Inayat Ur Rahman, hajibotanist@outlook.com (ORCID: 0000-0003-3312-7975)

Environmental variables drive plant species composition and distribution in the moist temperate forests of Northwestern Himalaya, Pakistan

Abstract

Questions – By assessing plant species composition and distribution in biodiversity hotspots influenced by environmental gradients, we greatly advance our understanding of the local plant community and how environmental factors are affecting these communities, which is a proxy for assessing how impacts of climate change can affect plant communities located in mountainous regions ("space-for-time" substitution). We evaluated plant species composition and distribution, and how and which environmental variables drive the plant communities in moist temperate forest of Northwestern Himalaya, Pakistan.

Location – Moist temperate zone of Manoor valley (District Mansehra, Khyber Pakhtunkhwa), Northwestern Himalayan belt of Pakistan.

Methods – During four consecutive years (2015-2018), we sampled 30 sampling sites, measuring 21 environmental variables and recording all plant species present in an altitudinal gradient range of 1932-3168 m.a.s.l. We used different multivariate analyses to identify potential plant communities, and to determine the relative importance of each environmental variable in the species composition and distribution. Finally, we also evaluated diversity patterns, by comparing diversity indices and beta diversity processes.

Results – We found that (i) the moist temperate zone in this region can be divided in four different major plant communities; (ii) each plant community has a specific set of environmental drivers; (iii) there is a significant variation in plant species composition between communities, in which six species contributed most to the plant composition dissimilarity; (iv) there is a significant difference of the four diversity indices between communities; and (v) plant

community structure is twice more influenced by the spatial turnover of species than by the species loss.

Conclusions – Overall, we showed that altitudinal gradients offer an important range of different environmental variables, highlighting the existence of micro-climates that drive the structure and composition of plant species in each micro-region. Each plant community along the altitudinal gradient ~~has~~ a set of environmental drivers, which lead to the presence of indicator species in each micro-region.

Key words: Environmental variables, ~~plant~~ species composition, ~~plant~~ species distribution, moist temperate forest, environmental gradients, hotspots of biodiversity, climate change, indicator species, ~~multivariate approaches~~, Northwestern Himalaya, plant community, ~~environmental drivers~~.

Introduction

Mountains are the most remarkable land forms on the earth‘s surface with major vegetation zones based upon environmental variations [1]. They provide habitat heterogeneity based on micro-environmental variation along the altitudinal gradient [2,3], in which environmental variables (ranging from direct and indirect effects of abiotic and biotic effects) are important factors to determine the boundaries of altitudinal zones [4,5]. It is known that altitude represents a complex gradient along which many environmental factors [6] and species diversity [7,8] change concomitantly. Within this focus, plant biodiversity is strongly influenced by different environmental variables [9], in which some plant species can survive at the edge of life in high mountains all over the world [10–12].

Due to the high variation in the environmental variables along altitudinal gradients, many mountains worldwide are important hotspots of biodiversity [13–16], making up about 50% of all plant species growing in hotspots areas [17,18]. However, despite this high endemism of species greatly influenced by various environmental gradients, such as edaphic, climatic and physiographic variables [19], these areas suffer a major impact from climate change [20,21]. Plant species present in these gradients have great adaptive power [22,23]; however, the speed with which climate change is advancing might not be sufficiently achieved by plant species adaptation in these areas, leading to a strong impact on the biodiversity of these areas [24–26], ultimately leading to variations in the community structure [27].

In the face of the current climate change and considering the importance of phytosociological studies for the understanding of biodiversity and species distribution, the Himalayas represents a fundamental piece for these studies. This region is facing a marked increase in temperature [28], which is three times greater than the global average [29]. This unprecedented rise in temperature, and modification of various environmental variables as well, may lead to shifts in species composition [24–26] and variations in community structure [27]. Although there has been a considerable increase in the number of phytosociological studies in these altitudinal regions considered hotspots of biodiversity [7,19,22,23,30], there is still limited knowledge of how and which environmental variables drive the distribution and composition of plant species along altitudinal gradients in specific hotspots regions, such as the Northwestern Himalaya.

By assessing the patterns of composition and distribution of plant species in these biodiversity hotspots influenced by environmental gradients, we greatly advance our understanding of the local plant community and how environmental factors are affecting these

communities, which is a proxy for assessing how impacts of climate change can affect plant communities located in mountainous regions [31]. In this context, we evaluated plant species composition and how and which environmental variables drive the plant species distribution of moist temperate zone of the Northwestern Himalaya, Pakistan. Briefly, we assessed (i) the potential plant communities present in the moist temperate zone; (ii) which are the environmental variables that most determine plant community structure in the moist temperate zone; (iii) whether there is variation in plant species composition between plant communities and which are the species that most contributed for species composition dissimilarity; (iv) whether there is variation of diversity indices among communities; and finally (v) which is the beta diversity process that most influence plant community structure in the moist temperate zone.

Materials and methods

Study area

The present study was carried out in the moist temperate zone of Manoor valley (District Mansehra, Khyber Pakhtunkhwa), which is a mountainous valley (34.68165 N to 34.83869 N latitude, and 73.57520 E to 73.73182 E longitude Fig. 1) in the Northwestern Himalayan belt of Pakistan [32–36] along an altitudinal range of 1932-3168 m.a.s.l. Monsoon winds are the main source of precipitation as well as the primary force of controlling erosion, topography, climate and vegetation of the Northwestern Himalaya [1].

Ethics approval and consent to participate

This study was approved by the Board of Study (BOS), Committee, Department of Botany and Advanced Study Research Board (ASRB) of Hazara University, Mansehra 21300, KP, Pakistan.

Vegetation Sampling and plant identification

In different growing seasons (from March to October), we evaluated 30 stands per year, during four consecutive years, from 2015 to 2018. The line transect method (50 meters) we used for quantitative samplings [37–42], but we never repeated the same transects over years. The surveyed stands (study area) were subdivided into 30 stands and three points randomly selected were sampled within each stand along 50 meters transect (total = 90 transects). The interval distance kept between the stands was 200 meters and 100 meters between the transects. The individuals of plant species falling precisely on the line were recorded. The phytosociological attributes (*i.e.*, density, frequency, cover and their relative values, and importance value) were used for calculation of the data from each stand [43–45]. The IV was further used to rank each plant species and species with the highest IV were considered as the dominant species [43,44]. Similarly, the plant communities were named on the basis of three dominant species [46–49]. The slope angle, aspect and exposure were recorded using clinometer; and altitude, longitude and latitude were measured by geographical positioning system (GPS). Plants specimen collection, labeling, pressing and other herbarium work methodology was adopted following [50,51]. ~~Their~~ identification was done with the aid of Flora of Pakistan [52–54] and submitted to the Herbarium of Hazara University, Mansehra (Pakistan).

Environmental gradients

Soil samples of 200-400grams were collected from three randomly selected transects (0-30cm depth) within each sampling stand of the studied vegetation area [55,56]. The replicated samples collected from each stand were thoroughly mixed to make a composite sample [57], stored in a sterile polythene bag and labeled with a permanent marker. Raw materials like pieces of rocks

and stones were removed by sieving and then the samples were shade dried. Physicochemical analyses [57,58] like soil texture (*i.e.*, clay, sand, silt, loam), pH [59], electrical conductivity (EC) [60], organic matter (OM) [61], nitrogen (N), phosphorus (P), potassium (K) and calcium carbonate ($CaCO_3$) concentrations were determined for each soil sample [55,56,62]. Moreover, other climatic gradients (*i.e.*, barometric pressure, dew point, humidity, heat index, temperature, wet bulb and wind speed) were determined using a small remote weather station (Kestrel weather tracker 4000) to record the data at each transect and then average values were calculated at stand level [19].

Statistical analyses

All the collected data of plant species, edaphic and other environmental gradients ~~data~~ were prepared to find out the relationship among them [63,64]. Matrixes of IV data of all the recorded plant species (244 species) from 30 sampling sites were used in the analyses. Analyses were conducted on PC-ORD [65] and R software 4.0.0 [66]. Packages used in R software are mentioned in each analysis. A georeferenced map was prepared to show the distribution pattern of distinct plant communities (Fig. 1).

Sampling effort and Cluster analyses. Species area curves (SAC) were used to check the efficiency of the sampling effort, where plant abundance data with Sørensen distance values were used to create species area curves [67]. For classification of the recorded plant species (244) and 30 stands into different plant communities, we used three different cluster analyses: Two-way Indicator Species Analysis (TWINSPAN), Cluster Analysis (CA), and Two-Way Cluster Analysis (TWCA). We identified and classified plant species and stands into major plant communities [68], as well as assess the effects of various factors (such as environmental variables) on such communities by processing clustering method using TWINSPAN [19,69].

Plant communities and associated environmental variables. Both species and stands (sampling sites) were constrained in relation with the environmental gradients [70,71], which were divided into geographic, slope aspect, edaphic and climatic gradients. We used non-metric multidimensional scaling (NMDS) and Principal Component Analysis (PCA) ordination biplot to determine the relationship between vegetation communities and environmental gradients using the “vegan” package. In NMDS and PCA, arrows represent the environmental gradients, in which the length shows the strength of the gradient, and the direction represent the degree of correlation. The direction of gradients on the same axis reveals a positive correlation.

In addition, we performed canonical correspondence analysis (CCA) and variation partitioning tests (partial CCA) to observe how explanatory variables (climatic, edaphic, geographic, and slope) drive the plant species distribution. First we built the best model with the lowest number of variables (those that most explain variance), through the *step* function with permutation using the “stats” package [66]. Next, we also evaluated multicollinearity between variables of the final model using Variance Inflation Factor (VIF), and we removed any variable with VIF>10, one at a time. Finally, with the final model, we carried out CCA and partial CCA to check how much each group of variables (geographic, edaphic, climatic, slope) explain in our model [72]. For these analysis, we used the “vegan” package [73].

Variation of plant species composition among plant communities. To compare whether there is difference in species composition between plant communities, we also used NMDS followed by a Permutational Multivariate Analysis of Variance (PERMANOVA) with Euclidean distance and 999 permutations. After PERMANOVA, we conducted pairwise comparisons between communities with corrections for multiple testing also using Euclidean distance and

999 permutations. We used false discovery rate (FDR) as p-value adjustment method. PERMANOVA and pairwise comparisons were conducted with “RVAideMemoire” package [74]. To observe the contribution of each plant species to overall dissimilarities, we used a similarity percentage analysis based on the decomposition of Bray-Curtis dissimilarity using the package “vegan” [73].

Variation of environmental variables among plant communities. To compare the 21 environmental parameters evaluated among communities, we conducted a Generalized Linear Model (GLM) with Gaussian error distribution followed by Likelihood Ratio test using the ‘stats’ and ‘car’ [75] packages, respectively.

Variation of diversity indices among plant communities. To compare the diversity indices evaluated (species richness, Shannon, Simpson, and Pielou) among the four communities, we also conducted a GLM with Gaussian error distribution, except for species richness, in which we used Poisson distribution followed by Likelihood Ratio test. Pairwise comparisons were conducted with estimated marginal means using the package ‘emmeans’ [76].

Beta diversity. To evaluate which is the beta diversity process that most influence plant community structure in the moist temperate zone, we decomposed the Sørensen dissimilarity index (βsor), a measure of overall species replacement into two additive components: the spatial turnover (Simpson pairwise dissimilarity, βsim) and nestedness-resultant components (nestedness-fraction of Sorensen pairwise dissimilarity, βsne) [77–79]. Dissimilarity analysis was conducted in the package “betapart” [80].

Results

Sampling effort and Cluster analyses

A total of 244 plant species belonging to 194 genera and 74 families (Table S1) were recorded in moist temperate forest of Manoor valley, Northwestern Himalaya, Pakistan. The moist temperate forest ranged from 1932.3m to 3168m. SAC analysis showed that the maximum number of plant species appeared up to stand 26 and the species curve became parallel after it, as no new species were recorded further (Fig. 2). Based on the TWINSPAN analysis (high cluster heterogeneity value – Lambda = 0.4045), we identified four different major plant communities (Fig. 3), which were composed of different indicator species (IHC: *Indigofera heterantha-Heracleum candicans-Cynodon dactylon;* VIP: *Viburnum grandiflorum-Indigofera heterantha-Pinus wallichiana,* CPI: *Cedrus deodara-Pinus wallichiana-Isodon rugosus*, and PCP: *Pinus wallichiana-Cedrus deodara- Parrotiopsis jacquemontiana*).

The IHC community was primarily found in the lower mountainous ranges (1932.3-2338.4 m.a.s.l), where the dominating flora was a combination of shrub and herb species owing to the existence of a substantial herbaceous layer of *Cynodon dactylon*, which carpeted the landscape alongside *Indigofera heterantha* patches. The VIP community was recognized mainly in the foothills and adjacent plains (2390.5-2437.8 m.a.s.l), where the predominant vegetation was shrubland with abundant patches of *Viburnum grandiflorum* and *Indigofera heterantha*, accompanied by the co-dominant *Pinus wallichiana* (tree species). Nonetheless, the other two plant communities, i.e., PCP and CPI, were significantly dominated by the tree species layer at the middle (2292-2947 m.a.s.l) and higher (2048.2-3168 m.a.s.l) altitudinal ranges alongside shrubby associates.

Plant communities and associated environmental variables

Correlation between moist temperate forest plant communities and environmental gradients were illustrated through NMDS (Fig. 4a-d) and PCA (Fig. 4e). Communities were related to specific

environmental variables such as geographic, slope, edaphic, and climatic variables (Table S2). Altitude, slope angle and aspects (SE, NE, ES, WN), K, pH, OM, loam, silt, sand, clay, temperature, heat index, wind speed and barometric pressure were the most representative environmental variables which drive the plant community structure and diversity along the altitudinal gradient. In both ordinations, the environmental variables separate 30 sampling sites into four major plant communities, as also showed by the TWINSPAN method (Fig. 3). In constrained PCA ordination, the maximum explanatory variation was accounted on PC1 axis (20%) and lower variation on PC2 axis (14.2%). Maximum strength was recorded for the most important environmental gradient, *i.e.*, profound influence of the environmental gradients was revealed by dividing the vegetation of the moist temperate forest into four communities (Fig. 4e), as also showed by TWINSPAN and NMDS.

The PCP community showed positive and significant correlation with northern aspect, silty loamy soil texture, humidity and altitude (Fig. 4b-c). In contrast, CPI community showed positively significance with southern slope, wind speed, dew point and wet bulb. IHC community showed positively significance with silty soil texture, electric conductivity and pH. And finally, VPI community revealed positively significant correlation with north-western slope, $CaCO_3$ and pH. Thus, all the four communities were found separately in clumps with clear differences based on the environmental gradients (Fig. 4).

The CCA and variation partitioning tests showed that the total inertia results of CCA was 3.023, where our final variables (Altitude, Temperature, Humidity, Wind speed, Slope Angle, Slope N, Slope NW, Slope SW, pH, EC, OM, $CaCO_3$, K, P, Sand, and Loam) together explained 66.5% of variation (sum of canonical eigenvalues was 2.011). The first two canonical axes explained 37.1% of variation. CCA model was significant (χ^2=2.010; pseudo-F value=1.613;

p<0.001; df=16; permutations=999). For the 16 explanatory variables, we tested simple term effects. Simple term effects showed that Altitude, Slope SW, Slope NW, Slope N, Slope Angle, K, and Humidity (decreasing order of importance) were significant (p<0.05; Table 1). The 16 explanatory variables were grouped into four classes: Climatic (Humidity, Temperature, Wind speed); Edaphic (pH, EC, OM, $CaCO_3$, K, P, Sand, Loam); Geographic (Altitude); and Slope (Slope Angle, Slope N, Slope NW, Slope SW), and then, we performed variation partitioning tests (partial CCA) for all 15 possible classes (Table 2). Class [b] was the most explanatory variable (104.6%) followed by class [m] (7.2%) (Fig. 5).

Variation of plant species composition among plant communities

We found a significant variation in plant species composition between communities (Table 3; Fig. 6), in which all communities showed a significant difference in species composition between each other (Table 4). Out of 244 species, six species greatly contributed to the variation in plant species composition between communities, namely *Viburnum grandiflorum*, *Indigofera heterantha*, *Heracleum candicans*, *Cedrus deodara*, *Pinus wallichiana*, and *Parrotiopsis jacquemontiana* (Table 4). Overall, the three species that most contributed for the variation in species composition between communities showed 13.7-29.7 % of cumulative contribution (Table 4).

Variation of environmental variables among plant communities

GLM analyses of 21 studied variables distributed in different groups (geographic, slope, climatic and edaphic gradients) between four plant communities showed that most of the environmental variables were significantly different between communities (p-value<0.05; Fig. 7). Nonetheless, slope angle, heat index, temperature, wind speed, dew point, wet bulb, pH, organic matter, electric conductivity, potassium and phosphorous did not show significant difference.

Variation of diversity indices among plant communities

We found a significant difference of four diversity indices, species richness (GLM χ^2=73.113, df=3, p<0.001; Fig. 8a), Shannon (GLM χ^2=35.797, df=3, p<0.001; Fig. 8b), Simpson (GLM χ^2=46.465, df=3, p<0.001; Fig. 8c), and Pielou (GLM χ^2=44.093, df=3, p<0.001; Fig. 8d), between the four communities. PCP showed the highest average number of species (68.1±4.2; mean±SE) followed by VIP (53.3±7.5) and IHC (51.1±3.5), and finally by CPI, with the lowest number of species (40.2±4.5) (Fig. 8a). PCP showed a Shannon' value of 3.62±0.08 (mean±SE), followed by IHC (3.53±0.07), VIP (3.29±0.1), and CPI (2.9±0.1) respectively (Fig. 8b). IHC showed the highest Simpson' value (0.959±0.01; mean±SE), followed by PCP (0.954±0.01), VIP (0.930±0.01), and CPI (0.898±0.01) respectively (Fig. 8c). Finally, IHC showed the highest Pielou' value (0.901±0.01; mean±SE), followed by PCP (0.862±0.01), VIP (0.830±0.01), and CPI (0.797±0.01) respectively (Fig. 8d).

Beta diversity

The total beta diversity (βsor) showed a value of 54.7% dissimilarity, of which spatial turnover (βsim) made up 40.5% and nestedness-resultant components (βsne) made up 14.2%. In βsim cluster, we observed 47.8% dissimilarity between PCP-CPI cluster and VIP-IHC cluster (Fig. 9). PCP showed a dissimilarity of 9.4% with CPI, and VIP showed a dissimilarity of 21.5% with IHC (Fig. 9). In βsne cluster, we found 24.5% dissimilarity between PCP and VIP-CPI-IHC cluster (Fig. 9). VIP showed a dissimilarity of 11.3% with IHC-CPI, and IHC had 4.1% dissimilarity with CPI (Fig. 9). Thus, plant community structure is twice more influenced by the spatial turnover of species (βsim) than by the species loss (nestedness-resultant, βsne).

Discussion

Mountain ecosystems are characterized by dramatic changes in temperature and abiotic properties over short altitudinal and geographical distances, making them ideal natural laboratories for studying vegetation response to environmental changes [81]. In this study, we evaluated the plant species composition and distribution in a hotspot of biodiversity, the Northwestern Himalayan mountains, Pakistan, assessing how environmental gradients, source of habitat heterogeneity, influence plant community structure and diversity, which might be a proxy for assessing how climate change impacts on plant communities located in mountainous regions [31,82,83]. We found that (i) the moist temperate zone in this region can be divided in four different major plant communities; (ii) each plant community has a specific set of environmental drivers; (iii) there is a significant variation in plant species composition between communities, in which six species contributed most to the plant composition dissimilarity; (iv) there is a significant difference of the four diversity indices (species richness, Shannon, Simpson, Pielou) between communities; and finally (v) plant community structure is twice more influenced by the spatial turnover of species (βsim) than by the species loss (nestedness-resultant, βsne). Overall, we showed that altitudinal gradients offer an important range of different environmental variables, highlighting the existence of micro-climates that drive the structure and composition of plant species in each micro-region. In addition, each plant community along the altitudinal gradient has a set of environmental drivers, which lead to the presence of indicator species in each micro-region.

Mountain plant communities are thought to be sensitive to climate change and, thus, able to reveal its effects sooner than others [31,84]. The four communities found showed a wide range of environmental drivers; however, altitude and temperature showed great prominence, probably making up the main environmental drivers in mountainous plant communities. Similar pattern

was observed in the allied area (Nandiar catchment, Battagram) of Northwestern Himalaya by stating altitude and temperature as the governing gradient [69]. Such variables, which can be strongly correlated [85], modify the diversity and structure of plant communities by creating local micro-climates [86], directly influencing plant community composition and diversity [19,26,87,88].

Indeed, there is no order of importance of environmental variables, but studies are unanimous in showing that there is a consensus on the explanations for the variables' influences. For instance, in two recent studies we showed that the altitude-temperature relationship significantly influenced the physiological attributes of some plant species in the Northwestern Himalayan region [22,23], which can be a proxy for understanding plant adaptation to climate change. Any change in soil parameters has a significant effect on the growth of plant communities [19]. The studies on mountain forest habitats around the world have also revealed the role of soil structure on species zonation [67,89,90]. Furthermore, both chemical and physical attributes of the soil are related to natural soil characteristics, with an impact on plant species composition and distribution of higher vascular plants [91–93]. For instance, some soil variables can have great influence on plant composition and distribution, such as pH. Some studies have shown that pH level on soil can influence nutrient availability, ultimately influencing nutrient uptake for growth [94–96]. However, the availability of some nutrients as a result of pH levels can be detrimental for some plants, since some nutrients are toxic to some plants [94,97]. Considering that there is a great variability of pH levels and nutrient availability and concentration along altitudinal gradients, it is expected a great variability of plant species composition which can be more or less related to specific soil parameters. Since plants are sensitive to small variations of soil characteristics such as pH, minerals, organic matter, among

others, and these variables are constantly changing along altitudinal gradients directly and indirectly influencing the presence and availability of other organisms and resources, some plant species might have adapted to specific set of variables.

Variability in plant species diversity is an outcome of species interaction with particular set of environment variables either abiotic and biotic [98,99], which can occur in both space and time [100,101]. The concept of changing species composition and vegetation continuum along the ecological gradients emerged as an antithesis model for distinct units [102,103]. In our study, the moist temperate forest of the studied Northwestern Himalayan region is comprised in an altitudinal gradient of approximately 1500 m. This gradient is subject to strong micro-climatic variation, which results in a set of micro-regions (better discussed above). Each micro-region has certain characteristics, which will influence the set of species that will inhabit these spaces [24–26]. In this sense, it is expected that the plant community structure is more influenced by the spatial turnover of species (βsim) than by the species loss (nestedness-resultant, βsne), *i.e.*, that there are different plant communities along the altitudinal gradient, as shown by our results. The differentiation of species diversity was mainly a consequence of environmental variables which is due to soil factors [104]. Therefore, in addition to the influence of edaphic factors in space on species composition and vegetation continuum, as shown in our study, results from similar studies have shown that the altitude is also important in driving vegetation structure and diversity in plant communities.

We found that environmental heterogeneity among plant communities have significant effects on beta diversity, particularly the spatial turnover. These results indicate that there is not a significant loss of the number of species between the plant community, but a variation in the species composition. This variation may be closely linked to the environmental effects in the

area, which induces the appearance of species adapted to environmental variables. The local community composition replacement implied the simultaneous loss and gain of species due to immigration–extinction dynamics and trait- based environmental filtering [105,106]. This indicates the relationship among plant community types and among species based on multiple factors. Although we did not find a large variation in βsne (loss of species between plant communities), it is important to note that temporal analysis might be important to consider a notable variation in this component of beta diversity; and βsne variations will be better observed in long-term analysis in future studies. In this sense, it is expected that the plant community structure is more influenced by the spatial turnover of species (βsim) than by the species loss (nestedness-resultant, βsne), *i.e.*, that there are different plant communities along the altitudinal gradient, as shown by our results. Similarly, results were report by Haq et al. [107] from forest of Kashmir Himalaya, India.

We observed a significant variation in plant species composition between communities, in which all communities showed a significant difference in species composition between each other. The measure of Bray-Curtis dissimilarity shows that species composition change that is influenced mainly by abundant species, in our study six species *(Viburnum grandiflorum, Indigofera heterantha, Heracleum candicans, Cedrus deodara, Pinus wallichiana,* and *Parrotiopsis jacquemontiana)* contributed most to the plant composition dissimilarity. These results suggest that the richness and turnover patterns we observed were driven primarily by rare species, which comprise most of the local species pools at these forest communities [108]. These findings are consistent with the idea that less abundant species are more sensitive to climate variability than longer lived and more abundant species [109]. The high level of turnover is

common and is an important mechanism by which a large regional species pool buffers site level diversity from interannual variation in climate [110].

Current study provides the baseline and first insights of spatial distribution, vegetation pattern and species contribution in response to environmental gradients in a moist temperate forest, Northwestern Himalaya, Pakistan. Studies that evaluate the distribution and composition of the plant community are fundamental for a better understanding of the local plant community, the conservation status and protection of these communities, as well as providing support for mitigation measures. Especially in the case of Northwestern Himalaya, which represents a biodiversity hotspot, it is even more important that we conduct phytosociological studies in these areas in order to preserve the biodiversity there. In the face of current climate changes, these regions are being heavily impacted [28,29], where the probability of species extinction may be higher than elsewhere, as these regions are rich in endemic species. Finally, we need to consider that phytosociological studies consider a general profile of the first trophic chains level, *i.e.*, to evaluate the composition, distribution and diversity of plants is to indirectly assess the first level of trophic chains.

Supporting information

S1 Table. Species composition and IV according to each stand and community found along four years of collection in moist temperate forest of Manoor valley, Himalaya, Pakistan. IHC: *Indigofera heterantha- Heracleum candicans-Cynodon dactylon,* VIP: *Viburnum grandiflorum-Indigofera heterantha-Pinus wallichiana,* CPI: *Cedrus deodara-Pinus wallichiana-Isodon rugosus* and PCP: *Pinus wallichiana-Cedrus deodara- Parrotiopsis jacquemontiana*.

S2 Table. Mean (SD) of environmental variables and plant species richness per community found along four years of collection in moist temperate forest of Manoor valley, Northwestern Himalaya.

Competing Interests Statement

Authors declare that there is no conflict of interest.

Acknowledgments

We thank the Higher Education Commission (HEC), Pakistan, for granting a scholarship under the International Research Support Initiative Program (IRSIP) to conduct a research work at Missouri Botanical Garden, Saint Louis, MO, USA (IUR). The authors would like to extend their sincere appreciation to the Deanship of Scientific Research at King Saud University for its funding to the Research Group number (RG-1435-014).

Author Contributions

I.U.R: Conceptualization, Investigation, Methodology, Software, Data curation, Formal analysis, Writing- Original draft preparation. R.H: Supervision, Methodology, Writing – review & editing, Validation. A.A: Supervision, Methodology, Visualization, Validation. Z.I: Supervision, Methodology, Validation. E.S.C: Software, Writing – review & editing, Validation. E.F.A., A.A.A. A.H., and SMH.: Literature gathering, helped in discussion. F.I: Data curation, herbarium work.

References

1. Khan SM. Plant communities and vegetation ecosystem services in the Naran Valley, Western Himalaya. PhD Dissertation, University of Leicester, UK. 2012.
2. Luo Z, Tang S, Li C, Fang H, Hu H, Yang J, et al. Environmental effects on vertebrate species richness: testing the energy, environmental stability and habitat heterogeneity hypotheses. PLoS One. 2012/04/18. 2012;7: e35514–e35514. doi:10.1371/journal.pone.0035514

3. Yang Z, Liu X, Zhou M, Ai D, Wang G, Wang Y, et al. The effect of environmental heterogeneity on species richness depends on community position along the environmental gradient. Sci Rep. 2015;5: 15723. doi:10.1038/srep15723
4. Shipley B, Keddy PA. The individualistic and community-unit concepts as falsifiable hypotheses. Theory and models in vegetation science. Springer; 1987. pp. 47–55.
5. Frahm J-P, Gradstein SR. An altitudinal zonation of tropical rain forests using byrophytes. J Biogeogr. 1991; 669–678.
6. Givnish TJ. On the causes of gradients in tropical tree diversity. J Ecol. 1999;87: 193–210.
7. Iqbal Z, Zeb A, Abd_Allah EF, Rahman IU, Khan SM, Ali N, et al. Ecological assessment of plant communities along the edaphic and topographic gradients of biha valley, District Swat, Pakistan. Appl Ecol Environ Res. 2018;16. doi:10.15666/aeer/1605_56115631
8. Pauli H, Gottfried M, Dullinger S, Abdaladze O, Akhalkatsi M, Alonso JLB, et al. Recent Plant Diversity Changes on Europe’s Mountain Summits. Science (80-). 2012;336: 353 LP – 355. doi:10.1126/science.1219033
9. Bellard C, Bertelsmeier C, Leadley P, Thuiller W, Courchamp F. Impacts of climate change on the future of biodiversity. Ecol Lett. 2012;15: 365–377.
10. Kullman L. Alpine flora dynamics – a critical review of responses to climate change in the Swedish Scandes since the early 1950s. Nord J Bot. 2010;28: 398–408. doi:10.1111/j.1756-1051.2010.00812.x
11. Jabis MD. Climate Change Impacts in Alpine Plant Communities. UC Berkeley; 2018.
12. Malanson GP, Nelson EL, Zimmerman DL, Fagre DB. Alpine plant community diversity

in species–area relations at fine scale. Arctic, Antarct Alp Res. 2020;52: 41–46. doi:10.1080/15230430.2019.1698894

13. Cañadas EM, Fenu G, Peñas J, Lorite J, Mattana E, Bacchetta G. Hotspots within hotspots: Endemic plant richness, environmental drivers, and implications for conservation. Biol Conserv. 2014;170: 282–291. doi:https://doi.org/10.1016/j.biocon.2013.12.007

14. Noroozi J, Talebi A, Doostmohammadi M, Rumpf SB, Linder HP, Schneeweiss GM. Hotspots within a global biodiversity hotspot - areas of endemism are associated with high mountain ranges. Sci Rep. 2018;8: 10345. doi:10.1038/s41598-018-28504-9

15. Kidane YO, Steinbauer MJ, Beierkuhnlein C. Dead end for endemic plant species? A biodiversity hotspot under pressure. Glob Ecol Conserv. 2019;19: e00670. doi:https://doi.org/10.1016/j.gecco.2019.e00670

16. Noroozi J, Naqinezhad A, Talebi A, Doostmohammadi M, Plutzar C, Rumpf SB, et al. Hotspots of vascular plant endemism in a global biodiversity hotspot in Southwest Asia suffer from significant conservation gaps. Biol Conserv. 2019;237: 299–307. doi:https://doi.org/10.1016/j.biocon.2019.07.005

17. Motiekaitytė V. Conservation diversity of vascular plants and their communities in situ, applying the conception of ecosystem pool. Ekologija. 2006;2: 1–7.

18. Myers N, Mittermeier RA, Mittermeier CG, Fonseca GAB da, Kent J. Biodiversity hotspots for conservation priorities. Nature. 2000;403: 853–858. doi:doi.org/10.1038/35002501

19. Rahman IU, Afzal A, Iqbal Z, Bussmann RW, Alsamadany H, Calixto ES, et al. Ecological gradients hosting plant communities in Himalayan subalpine pastures:

Application of multivariate approaches to identify indicator species. Ecol Inform. 2020;60: 101162. doi:https://doi.org/10.1016/j.ecoinf.2020.101162

20. Baldwin AH, Jensen K, Schönfeldt M. Warming increases plant biomass and reduces diversity across continents, latitudes, and species migration scenarios in experimental wetland communities. Glob Chang Biol. 2014;20: 835–850.

21. Rudgers JA, Kivlin SN, Whitney KD, Price M V, Waser NM, Harte J. Responses of high- altitude graminoids and soil fungi to 20 years of experimental warming. Ecology. 2014;95: 1918–1928.

22. Rahman IU, Afzal A, Iqbal Z, Hart R, Abd_Allah EF, Alqarawi AA, et al. Response of plant physiological attributes to altitudinal gradient: Plant adaptation to temperature variation in the Himalayan region. Sci Total Environ. 2020;706: 135714. doi:10.1016/j.scitotenv.2019.135714

23. Rahman IU, Hart R, Afzal A, Iqbal Z, Alqarawi AA, Abd_Allah EF, et al. Ecophysiological plasticity and cold stress adaptation in Himalayan alpine herbs: Bistorta affnis and Sibbaldia procumbens. Plants. 2019;8: 378–386. doi:10.3390/plants8100378

24. Schuur EAG, Crummer KG, Vogel JG, Mack MC. Plant species composition and productivity following permafrost thaw and thermokarst in Alaskan tundra. Ecosystems. 2007;10: 280–292.

25. Weltzin JF, Bridgham SD, Pastor J, Chen J, Harth C. Potential effects of warming and drying on peatland plant community composition. Glob Chang Biol. 2003;9: 141–151.

26. Rahman IU, Hart R, Afzal A, Iqbal Z, Abdallah EF, Alqarawi AA, et al. Phenological plasticity in Berberis lycium Royle along temporal and altitudinal gradients. Appl Ecol Environ Res. 2019;17: 331–341. doi:10.15666/aeer/1701_331341

27. Walker MD, Wahren CH, Hollister RD, Henry GHR, Ahlquist LE, Alatalo JM, et al. Plant community responses to experimental warming across the tundra biome. Proc Natl Acad Sci. 2006;103: 1342–1346.

28. Shrestha UB, Gautam S, Bawa KS. Widespread climate change in the Himalayas and associated changes in local ecosystems. PLoS One. 2012;7: e36741. doi:10.1371/journal.pone.0036741

29. Xu J, Grumbine RE, Shrestha A, Eriksson M, Yang X, Wang Y, et al. The melting Himalayas: Cascading effects of climate change on water, biodiversity, and livelihoods. Conserv Biol. 2009;23: 520–530. doi:10.1111/j.1523-1739.2009.01237.x

30. Khan AM, Qureshi R, Saqib Z. Multivariate analyses of the vegetation of the western Himalayan forests of Muzaffarabad district, Azad Jammu and Kashmir, Pakistan. Ecol Indic. 2019;104: 723–736.

31. Niu Y, Yang S, Zhou J, Chu B, Ma S, Zhu H, et al. Vegetation distribution along mountain environmental gradient predicts shifts in plant community response to climate change in alpine meadow on the Tibetan Plateau. Sci Total Environ. 2019;650: 505–514.

32. Rahman IU, Ijaz F, Afzal A, Iqbal Z, Ali N, Khan SM. Contributions to the phytotherapies of digestive disorders: Traditional knowledge and cultural drivers of Manoor Valley, Northern Pakistan. J Ethnopharmacol. 2016;192: 30–52. doi:10.1016/j.jep.2016.06.049

33. Rahman IU, Ijaz F, Iqbal Z, Afzal A, Ali N, Afzal M, et al. A novel survey of the ethno medicinal knowledge of dental problems in Manoor Valley (Northern Himalaya), Pakistan. J Ethnopharmacol. 2016;194: 877–894. doi:10.1016/j.jep.2016.10.068

34. Rahman IU, Afzal A, Iqbal Z, Ijaz F, Ali N, Bussmann RW. Traditional and

ethnomedicinal dermatology practices in Pakistan. Clin Dermatol. 2018;36: 310–319. doi:10.1016/j.clindermatol.2018.03.016

35. Rahman IU, Afzal A, Iqbal Z, Hart R, Abd_Allah EF, Hashem A, et al. Herbal teas and drinks: Folk medicine of the Manoor valley, Lesser Himalaya, Pakistan. Plants. 2019;8: 1–18. doi:10.3390/plants8120581

36. Rahman IU, Afzal A, Iqbal Z, Ijaz F, Ali N, Asif M, et al. First insights into the floristic diversity, biological spectra and phenology of Manoor valley, Pakistan. Pakistan J Bot. 2018;50: 1113–1124.

37. Buckland ST, Anderson DR, Burnham KP, Laake JL, Borchers DL, Thomas L. Introduction to distance sampling: estimating abundance of biological populations. 2001.

38. Buckland ST, Anderson DR, Burnham KP, Laake JL, Borchers DL, Thomas L. Advanced distance sampling. Oxford University Press Oxford; 2004.

39. Buckland ST, Newman KB, Fernández C, Thomas L, Harwood J. Embedding population dynamics models in inference. Stat Sci. 2007; 44–58.

40. Anderson DR, Burnham KP, Laake JL. Distance sampling: estimating abundance of biological populations. Chapman &Hall, London, United Kingdom. 1993.

41. Sprent P. Distance Sampling-Estimating Abundance of Biological Populations. JSTOR; 1994.

42. Le Moullec M, Pedersen ÅØ, Yoccoz NG, Aanes R, Tufto J, Hansen BB. Ungulate population monitoring in an open tundra landscape: distance sampling versus total counts. Wildlife Biol. 2017;2017.

43. Curtis JT, McIntosh R. The interrelations of certain analytic and synthetic phytosociological characters. Ecology. 1950;31: 434–455.

44. Curtis JT. The vegetation of Wisconsin: an ordination of plant communities. University of Wisconsin Pres; 1959.

45. Jayasuriya AHM, Pemadasa MA. Factors affecting the distribution of tree species in a dry zone montane forest in Sri Lanka. J Ecol. 1983; 571–583.

46. Son H-J, Park S-H, Lee D-H, Song J-H, Park H-W, Park W-G. Growing environment characteristics and vegetation structure of Vaccinium Oldhamii Miq. native habitats in Korea. Forest Sci Technol. 2019;15: 33–43.

47. Tian Z, Li H, Li W, Gan X, Zhang X, Fan Z. Structural characteristics and niches of dominant tree populations in Tetracentron sinense communities: implications for conservation. Bot Sci. 2018;96: 157–167.

48. Lou Y, Pan Y, Gao C, Jiang M, Lu X, Xu YJ. Response of plant height, species richness and aboveground biomass to flooding gradient along vegetation zones in floodplain wetlands, Northeast China. PLoS One. 2016;11: e0153972.

49. Song JH, Cheong EJ, Kim H, Kim MS, Kim SH. Variation of morphological characteristics and anthocyanin contents from fruit of Vaccinium oldhamii in Korea. J Korean Soc For Sci. 2015;104: 193–197.

50. Ijaz F. Biodiversity and traditional uses of plants of Sarban Hills, Abbottabad. M.Phil Dissertation, Hazara University Manehra-21300, Khyber Pakhtunkhwa, Pakistan. 2014.

51. Ijaz F, Rahman I., Iqbal Z, Alam J, Ali N, Khan S. Ethno-ecology of the healing forests of Sarban Hills, Abbottabad, Pakistan: an economic and medicinal appraisal. 1st ed. In: M. Ozturk KRH, editor. Plant and Human Health. 1st ed. Springer International Publishing AG; 2018. pp. 675–706. doi:https://doi.org/10.1007/978-3-319-93997-1_19

52. Nasir E, Ali SI. Flora West of Pakistan. Nasir E, Ali SI, editors. Karachi: National

Herbarium; 1971.

53. Ali SI, Nasir YJ. Flora of Pakistan. Ali SI, Nasir. YJ, editors. Islamabad: Department of Botany, University of Karachi, Karachi and National Herbarium; 1989.

54. Ali SI, Qaiser M. Flora of Pakistan. Karachi: Department of Botany, University of Karachi; 1995.

55. Rahman IU, Ijaz F, Afzal A, Iqbal Z. Effect of foliar application of plant mineral nutrients on the growth and yield attributes of chickpea (Cicer arietinum L.) Under nutrient deficient soil conditions. Bangladesh J Bot. 2017;46: 111–118.

56. Rahman IU, Afzal A, Iqbal Z, Ijaz F, Khan SM, Khan SA, et al. Influence of different nutrients application in nutrient deficient soil on growth and yield of onion. Bangladesh J Bot. 2015;44: 613–619.

57. Ravindranath NH, Ostwald M. Carbon inventory methods: handbook for greenhouse gas inventory, carbon mitigation and roundwood production projects. Springer International Publishing AG; 2007.

58. Haq F, Ahmad H, Iqbal Z. Vegetation description and phytoclimatic gradients of subtropical forests of Nandiar Khuwar catchment District Battagram. Pakistan J Bot. 2015;47: 1399–1405.

59. Mc Lean EO. Soil pH and Lime requirement. 2nd ed. In: Page AL, Miller RH, Keeney DR, editors. Methods of soil analysis Part 2 Chemical and Microbiological Properties. 2nd ed. Madison, Wisconsin, USA: American Society of Agronomy, Inc. Soil Science Society of America, Inc.; 1982. pp. 199–223.

60. Wilson MJ, Bayley SE. Use of single versus multiple biotic communities as indicators of biological integrity in northern prairie wetlands. Ecol Indic. 2012;20: 187–195.

61. Nelson DW, Sommers LE. Total carbon, organic carbon, and organic matter. 5th ed. In: Sparks DL, Page AL, Helmke PA, Loeppert RH, Soltanpour PN, Tabatabai MA, et al., editors. Methods ofSoil Analysis Part 3 Chemical Method. 5th ed. Madison, WI 53711, USA: Soil Science Society of America and American Society of Agronomy; 1996. pp. 961–1010. doi:https://doi.org/10.2136/sssabookser5.3.c34
62. Soltanpour PN. Determination of nutrient availability and elemental toxicity by AB-DTPA soil test and ICPS. 16th ed. Advances in soil science. 16th ed. NY: Springer, New York; 1991. pp. 165–190. doi:https://doi.org/10.1007/978-1-4612-3144-8_3
63. Šmilauer P, Jan L. Multivariate analysis of ecological data using CANOCO. 2nd ed. Cambridge CB2 8BS, United Kingdom: Cambridge University Press; 2014. doi:https://doi.org/10.1017/CBO9781139627061
64. Mayor JR, Sanders NJ, Classen AT, Bardgett RD, Clément JC, Fajardo A, et al. Elevation alters ecosystem properties across temperate treelines globally. Nature. 2017;542: 91–95. doi:10.1038/nature21027
65. McCune B, Mefford M. PC-ORD. Multivariate Analysis of Ecological Data. Version 6. Gleneden Beach, Oregon, USA: MjM Software Design; 2011.
66. R Core Team. R: A language and environment for statistical computing. R Foundation for Statistical Computing, Vienna, Austria. 2020.
67. Rahman IU. Ecophysiological plasticity and Ethnobotanical studies in Manoor Area, Kaghan Valley, Pakistan. PhD Dissertation, Hazara University, Mansehra 21300, Khyber Pakhtunkhwa, Pakistan. 2020.
68. Hill MO. TWINSPAN: a FORTRAN program for arranging multivariate data in an ordered two-way table by classification of the individuals and attributes. Cornell Univ.

1979.

69. Haq F, Ahmad H, Iqbal Z, Alam M, Aksoy A. Multivariate approach to the classification and ordination of the forest ecosystem of Nandiar valley western Himalayas. Ecol Indic. 2017;80: 232–241. doi:10.1016/j.ecolind.2017.05.047

70. Knapp AK, Fay PA, Blair JM, Collins SL, Smith MD, Carlisle JD, et al. Rainfall variability, carbon cycling, and plant species diversity in a mesic grassland. Science (80-). 2002;298: 2202–2205. doi:10.1126/science.1076347

71. McCune B. PC-ORD: an integrated system for multivariate analysis of ecological data. Abstr Bot. 1986;10: 221–225.

72. Legendre P. Studying beta diversity: ecological variation partitioning by multiple regression and canonical analysis. J Plant Ecol. 2008;1: 3–8. doi:10.1093/jpe/rtm001

73. Oksanen J, Blanchet F, Friendly M, Kindt R, Legendre P, Minchin D, et al. vegan: Community Ecology Package (R package Version 2.5-5). Accessed; 2019.

74. Hervé M. RVAideMemoire: Testing and Plotting Procedures for Biostatistics. 2020.

75. Fox J, Weisberg S. An R companion to applied regression. California: Sage Publications; 2018.

76. Lenth R. emmeans: Estimated Marginal Means, aka Least-Squares Means. 2020.

77. Baselga A, Orme CDL. betapart: an R package for the study of beta diversity. Methods Ecol Evol. 2012;3: 808–812.

78. Baselga MA. Package ‘ betapart .’ 2013.

79. Baselga A. Partitioning the turnover and nestedness components of beta diversity. Glob Ecol Biogeogr. 2010;19: 134–143. doi:10.1111/j.1466-8238.2009.00490.x

80. Baselga A, Orme D, Villeger S, De Bortoli J, Leprieur F, Logez M, et al. Partitioning

Beta Diversity into Turnover and Nestedness Components. Cran. 2018; 1–42.

81. Anslan S, Azizi Rad M, Buckel J, Echeverria Galindo P, Kai J, Kang W, et al. Reviews and syntheses: How do abiotic and biotic processes respond to climatic variations in the Nam Co catchment (Tibetan Plateau)? Biogeosciences. 2020;17: 1261–1279.

82. McCarty JP. Ecological consequences of recent climate change. Conserv Biol. 2001;15: 320–331.

83. Hansen AJ, Neilson RP, Dale VH, Flather CH, Iverson LR, Currie DJ, et al. Global change in forests: responses of species, communities, and biomes: interactions between climate change and land use are projected to cause large shifts in biodiversity. Bioscience. 2001;51: 765–779.

84. Malanson GP, Resler LM, Butler DR, Fagre DB. Mountain plant communities: Uncertain sentinels? Prog Phys Geogr Earth Environ. 2019;43: 521–543. doi:10.1177/0309133319843873

85. Cantonati M, Gerecke R, Bertuzzi E. Springs of the Alps-sensitive ecosystems to environmental change: from biodiversity assessments to long-term studies. Hydrobiologia. 2006;562: 59–96.

86. Adair KL, Lindgreen S, Poole AM, Young LM, Bernard-Verdier M, Wardle DA, et al. Above and belowground community strategies respond to different global change drivers. Sci Rep. 2019;9: 1–11. doi:10.1038/s41598-019-39033-4

87. Conti L, de Bello F, Lepš J, Acosta ATR, Carboni M. Environmental gradients and micro-heterogeneity shape fine-scale plant community assembly on coastal dunes. J Veg Sci. 2017;28: 762–773. doi:10.1111/jvs.12533

88. Rahman I-U, Afzal A, Abd_Allah EF, Iqbal Z, Alqarawi AA, Hashem A, et al.

Composition of plant communities driven by environmental gradients in alpine pastures and cold desert of northwestern Himalaya, Pakistan. Pakistan J Bot. 2021;53: 655–664. doi:10.30848/pjb2021-2(35)

89. Rawat M, Arunachalam K, Arunachalam A, Alatalo JM, Kumar U, Simon B, et al. Relative contribution of plant traits and soil properties to the functioning of a temperate forest ecosystem in the Indian Himalayas. Catena. 2020;194: 104671.

90. Hegazy AK, El-Demerdash MA, Hosni HA. Vegetation, species diversity and floristic relations along an altitudinal gradient in south-west Saudi Arabia. J Arid Environ. 1998;38: 3–13.

91. Marini L, Scotton M, Klimek S, Isselstein J, Pecile A. Effects of local factors on plant species speciesrichness and composition of Alpine meadows. Agric Ecosyst Environ. 2007;119.

92. Oduor C, Karanja N, Onwonga R, Mureithi S, Pelster D, Nyberg G. Enhancing soil organic carbon, particulate organic carbon and microbial biomass in semi-arid rangeland using pasture enclosures. BMC Ecol. 2018;18.

93. Yang Y, et al. Soil physicochemical properties and vegetation structure along an elevation gradient and implications for the response of alpine plant development to climate change on the northern slopes of the Qilian Mountains. J Mt Res. 2018;15: 1006–1019.

94. Vicherová E, Hájek M, Hájek T. Calcium intolerance of fen mosses: physiological evidence, effects of nutrient availability and successional drivers. Perspect Plant Ecol Evol Syst. 2015;17: 347–359.

95. Audorff V, Kapfer J, Beierkuhnlein C. The role of hydrological and spatial factors for the vegetation of Central European springs. J Limnol. 2011;70: 9–22.

96. Strohbach M, Audorff V, Beierkuhnlein C. Drivers of plant species composition in siliceous spring ecosystems: groundwater chemistry, catchment traits or spatial factors? J Limnol. 2009;68: 375–384.

97. Tyler T, Olsson PA. Substrate pH ranges of south Swedish bryophytes—Identifying critical pH values and richness patterns. Flora. 2016;223: 74–82.

98. Ricklefs RE. Evolutionary diversification and the origin of the diversity–environment relationship. Ecology. 2006;87: S3–S13. doi:10.1890/0012-9658(2006)87[3:EDATOO]2.0.CO;2

99. Gauthray-Guyénet V, Schneider R, Paré D, Achim A, Loi C, Sirois L. Influence of shifts over an 80-year period in forest composition on soil properties. Plant Soil. 2018;433: 111–125.

100. Suding KN, Lavorel S, Chapin FS, Cornelissen JHC, Díaz S, Garnier E, et al. Scaling environmental change through the community-level: A trait-based response-and-effect framework for plants. Glob Chang Biol. 2008;14: 1125–1140. doi:10.1111/j.1365-2486.2008.01557.x

101. Nicotra AB, Atkin OK, Bonser SP, Davidson AM, Finnegan EJ, Mathesius U, et al. Plant phenotypic plasticity in a changing climate. Trends Plant Sci. 2010;15: 684–692. doi:10.1016/j.tplants.2010.09.008

102. Vleminckx J, Schimann H, Decaëns T, Fichaux M, Vedel V, Jaouen G, et al. Coordinated community structure among trees, fungi and invertebrate groups in Amazonian rainforests. Sci Rep. 2019;9: 1–10. doi:10.1038/s41598-019-47595-6

103. Hu A, Wang J, Lu X, Zhou J. Mountain biodiversity and ecosystem functions : interplay between geology and contemporary environments. ISME J. 2019. doi:10.1038/s41396-

019-0574-x

104. Kang S, Niu J, Zhang Q, Zhang X, Han G, Zhao M. Niche differentiation is the underlying mechanism maintaining the relationship between community diversity and stability under grazing pressure. Glob Ecol Conserv. 2020;24: e01246.

105. Matthews TJ, Whittaker RJ. Neutral theory and the species abundance distribution: recent developments and prospects for unifying niche and neutral perspectives. Ecol Evol. 2014;4: 2263–2277.

106. Filgueiras BKC, Peres CA, Melo FPL, Leal IR, Tabarelli M. Winner–Loser Species Replacements in Human-Modified Landscapes. Trends Ecol Evol. 2021.

107. Haq SM, Calixto ES, Kumar M. Assessing Biodiversity and Productivity over a Small-scale Gradient in the Protected Forests of Indian Western Himalayas. J Sustain For. 2021;40: 675–694. doi:10.1080/10549811.2020.1803918

108. Cleland EE, Collins SL, Dickson TL, Farrer EC, Gross KL, Gherardi LA, et al. Sensitivity of grassland plant community composition to spatial vs. temporal variation in precipitation. Ecology. 2013;94: 1687–1696.

109. Bisbing SM, Urza AK, Buma BJ, Cooper DJ, Matocq M, Angert AL. Can long- lived species keep pace with climate change? Evidence of local persistence potential in a widespread conifer. Divers Distrib. 2021;27: 296–312.

110. Burrows MT, Hawkins SJ, Moore JJ, Adams L, Sugden H, Firth L, et al. Global- scale species distributions predict temperature- related changes in species composition of rocky shore communities in Britain. Glob Chang Biol. 2020;26: 2093–2105.

Table 1

The contribution and ranking of the studied variables in our partial CCA model. Significant variables are displayed in bold.

Variables	Df	ChiSquare	F	p-value
Altitude	1	0.251	3.229	**0.001**
Slope.SW	1	0.245	3.146	**0.001**
Slope.NW	1	0.178	2.297	**0.001**
Slope.N	1	0.214	2.748	**0.002**
Slope.Angle	1	0.163	2.097	**0.009**
K	1	0.140	1.798	**0.018**
Humidity	1	0.119	1.538	**0.039**
Wind.speed	1	0.114	1.472	0.069
CaCO3	1	0.098	1.270	0.151
Temp	1	0.081	1.049	0.376
OM	1	0.079	1.017	0.401
EC	1	0.076	0.976	0.474
P	1	0.070	0.907	0.578
Sand	1	0.069	0.895	0.590
Loam	1	0.056	0.724	0.864
pH	1	0.050	0.647	0.922
P	1	0.088	0.7295	0.820
K	1	0.075	0.6191	0.922

Table 2

Results of variation partitioning (partial CCA) of four environmental variable groups studied (see Fig. 5 for individual fraction letters code).

Individual Fraction	Adjusted R^2	Variation explained (%)	% of all	Df
[a]	0.020	5.5	0.1	1
[b]	0.370	104.6	2.3	4
[c]	0.004	1.2	0.0	8
[d]	0.015	4.3	0.1	3
[e]	0.020	5.7	0.1	0
[f]	-0.091	-25.6	-0.6	0
[g]	-0.005	-1.5	0.0	0
[h]	-0.001	-0.3	0.0	0
[i]	-0.045	-12.9	-0.3	0
[j]	-0.002	-0.6	0.0	0
[k]	0.011	3.1	0.1	0
[l]	0.019	5.3	0.1	0
[m]	0.026	7.2	0.2	0
[n]	0.006	1.7	0.0	0
[o]	0.008	2.3	0.1	0
Total explained	0.354	100.0	2.2	18
All variation	15.835	/	100	

Table 3

PERMANOVA results comparing species composition between the four communities found in Moist temperate forest. This analysis was made with Euclidean distance and 999 permutations. Pairwise comparisons between communities are depicted in Table 4.

	Df	Sums of Sqs	Mean Sqs	F	R^2	Pr(>F)
Communities	3	9961.1	3320.4	13.324	0.6059	0.001
Residuals	26	6479.1	249.2		0.3941	
Total	29	16440.2			1	

Table 4

Pairwise comparisons with FDR p-value adjustment method of species composition and contrast results of the contribution of individual species to the overall Bray-Curtis dissimilarity of species composition between the four communities found in moist temperate forest. We displayed only the three species that most contributed. Av. dis. – Average dissimilarity; SD – Standard deviation; Av Com1 – Average Community 1; Av Com2 – Average community 2; Cum. – Cumulative; Cont. – Contribution. Vib.gra**:** *Viburnum grandiflorum,* Ind.het**:** *Indigofera heterantha,* Her.can**:** *Heracleum candicans,* Ced.deo**:** *Cedrus deodara*, Pin.wal**:** *Pinus wallichiana*, Par.Jac**:** *Parrotiopsis jacquemontiana.*

**Communities**	***P-value***	**Species**	**Av dis**	**SD**	**Ratio**	**Av Com1**	**Av Com 2**	**Cum**	**Cum %**	**Cont %**
**IHC-VIP**	0.011	Vib.gra	0.1	0	5	0	24.4	0.1	12.2	12.2
		Ind.het	0.1	0	1.9	8.8	23.5	0.2	20.1	7.9
		Her.can	0	0	1.3	6.9	1.8	0.2	22.8	2.7
**IHC-CPI**	0.002	Ced.deo	0.1	0	3.8	0	22.5	0.1	10.9	10.9
		Pin.wal	0.1	0	5.3	0	20.3	0.2	20.8	9.9
		Ind.het	0	0	0.9	8.8	2.3	0.2	24.2	3.5
**IHC-PCP**	0.002	Pin.wal	0.1	0	2.7	0	16.2	0.1	6.9	6.9
		Ced.deo	0.1	0	3	0	16.1	0.1	13.8	6.9
		Par.jac	0	0	5.8	0.4	10.3	0.2	18	4.2
**VIP-CPI**	0.011	Vib.gra	0.1	0	5.1	24.4	0	0.1	11.6	11.6
		Ind.het	0.1	0	5.3	23.5	2.3	0.2	21.5	9.9
		Ced.deo	0.1	0	1.9	5.2	22.5	0.3	29.7	8.3
**VIP-PCP**	0.006	Vib.gra	0.1	0	3.3	24.4	1.4	0.1	9.5	9.5
		Ind.het	0.1	0	6.6	23.5	2.8	0.2	17.9	8.4
		Pin.wal	0	0	2.6	5.3	16.2	0.2	22.8	4.9
**CPI-PCP**	0.002	Ced.deo	0	0	1.2	22.5	16.1	0.1	5.4	5.4
		Par.jac	0	0	1.8	4.7	10.3	0.1	9.6	4.2
		Pin.wal	0	0	1.1	20.3	16.2	0.1	13.7	4

Figure Click here to access/download;Figure;Figures.docx

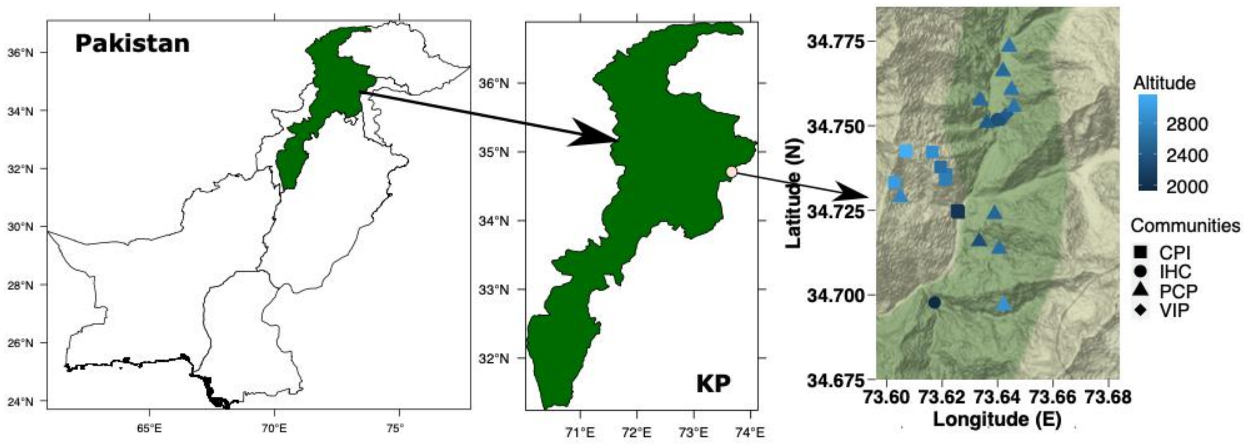

Figure 1. Map of the study area showing Pakistan country, Khyber Pakhtunkhwa (KP) province and stands of data collection. Points in the right figure represent stands of the four communities identified in the moist temperate zone, Himalaya region, Pakistan. **CPI:** *Cedrus deodara-Pinus wallichiana-Isodon rugosus,* **IHC**: *Indigofera heterantha- Heracleum candicans-Cynodon dactylon,* **PCP:** *Pinus wallichiana-Cedrus deodara- Parrotiopsis jacquemontiana***,** and **VIP:** *Viburnum grandiflorum-Indigofera heterantha-Pinus wallichiana.*

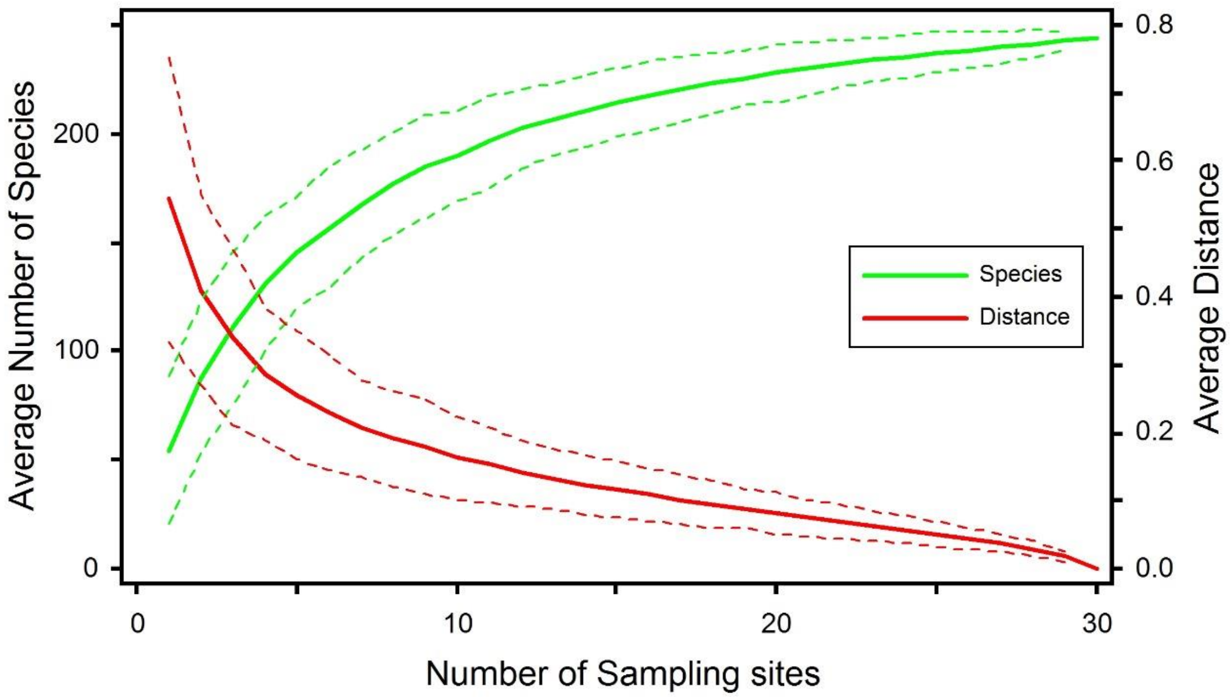

Figure 2. The species area curves of 244 plant species distributed among 30 sampling sites.

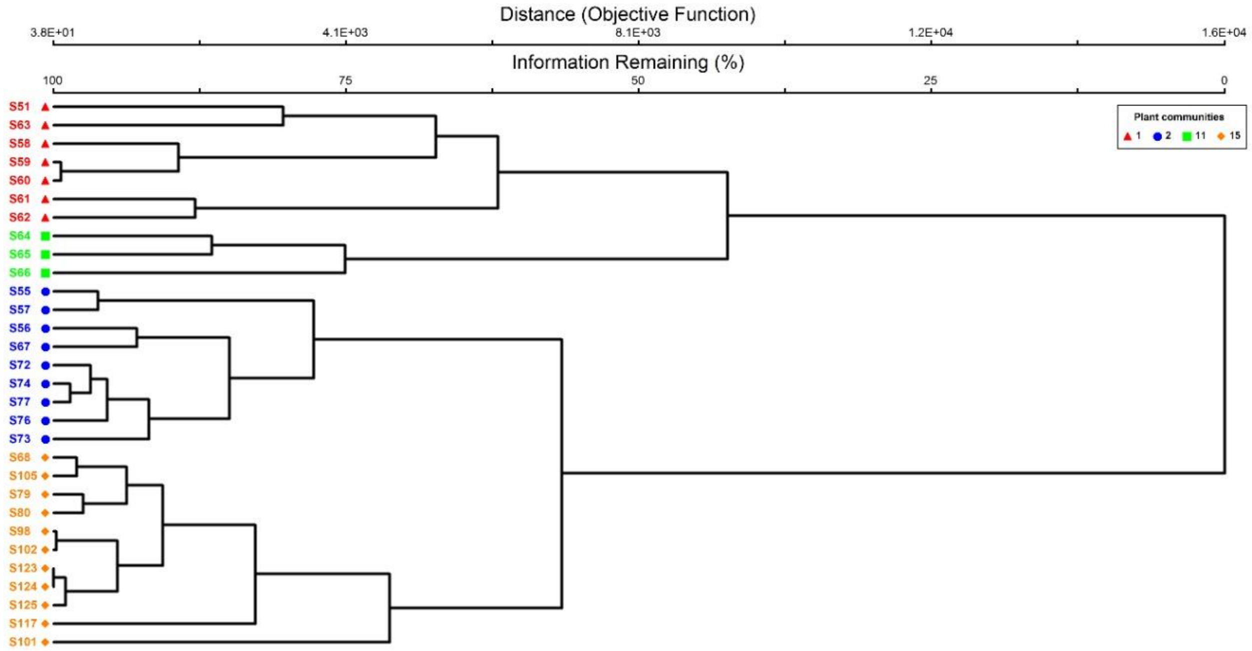

Figure 3. Clustering method using two-way indicator species analysis (TWINSPAN) indicating four different plant communities. IHC (red triangle): *Indigofera heterantha-Heracleum candicans-Cynodon dactylon*, VIP (blue circle): *Viburnum grandiflorum-Indigofera heterantha-Pinus wallichiana*, CPI (green square): *Cedrus deodara-Pinus wallichiana-Isodon rugosus* and PCP (yellow diamond): *Pinus wallichiana-Cedrus deodara- Parrotiopsis jacquemontiana*. Letters associated to numbers at the end of each branch of the dendrogram represent the stands evaluated. Letters associated to numbers at the end of each branch of the dendrogram represent the stands evaluated.

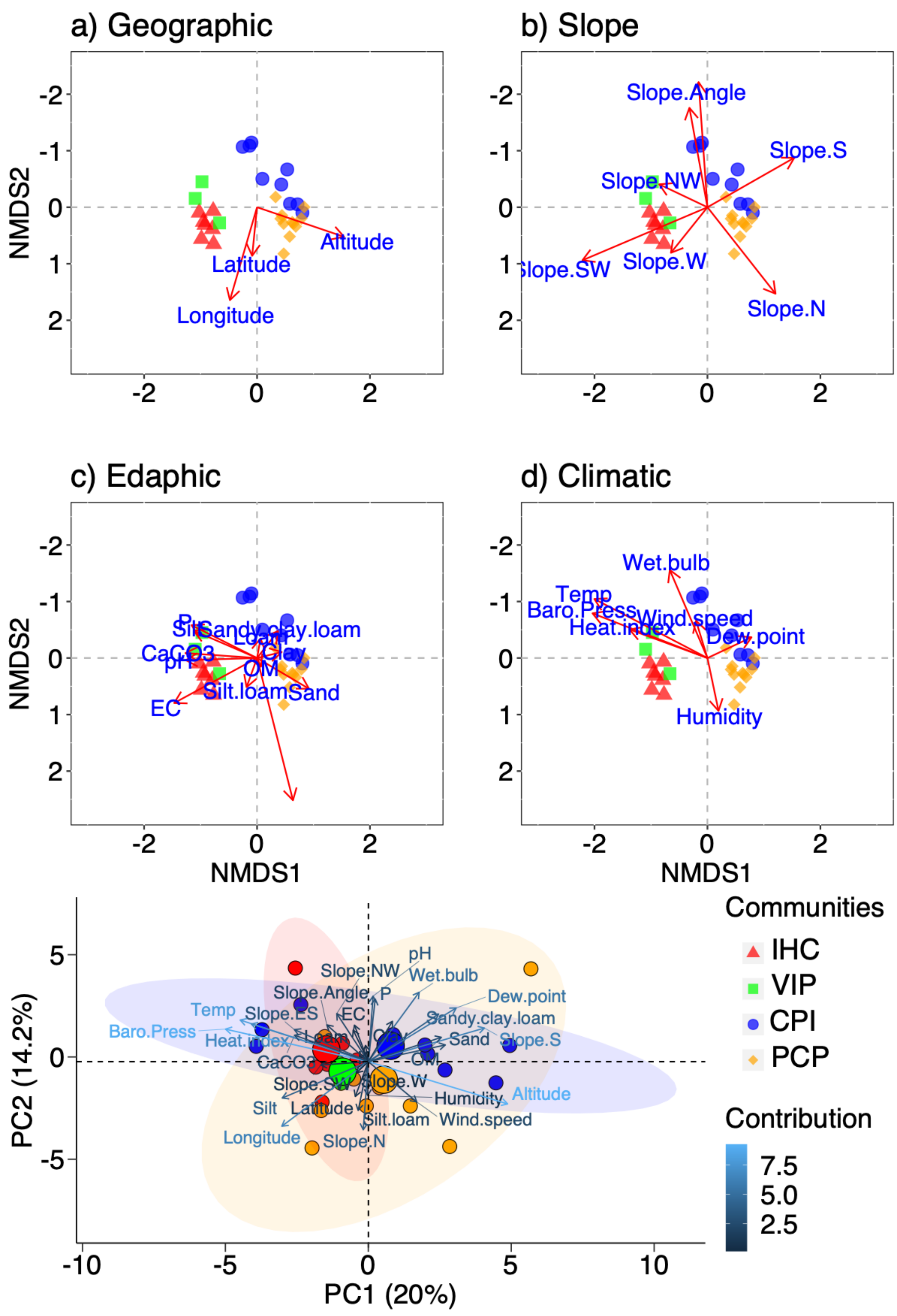
a) Geographic
b) Slope
c) Edaphic
d) Climatic
NMDS1
NMDS2
Latitude
Longitude
Altitude
Slope.Angle
Slope.S
Slope.NW
Slope.SW
Slope.W
Slope.N
Sandy.clay.loam
Loam
Clay
CaCO3
pH
OM
Silt.loam
Sand
EC
Wet.bulb
Temp
Baro.Press
Wind.speed
Heat.index
Dew.point
Humidity
PC1 (20%)
PC2 (14.2%)
Slope.ES
Silt
P
Communities
IHC
VIP
CPI
PCP
Contribution
7.5
5.0
2.5

Figure 4. Non-Multidimensional Scaling (NMDS) among plant communities of moist temperate forest and environmental gradients: a). geographic, b). slope, c). edaphic and d). climatic. e). Principle Component Analysis (PCA) illustrating the relationship of various measured environmental variables with communities indicated in coloured circles. Large coloured circles show the centroid of each community. NMDS-PCA: Species contribution analysis for community ordination in NMDS is depicted in Table 2. **IHC**: *Indigofera heterantha-Heracleum candicans-Cynodon dactylon,* **VIP:** *Viburnum grandiflorum-Indigofera heterantha-Pinus wallichiana,* **CPI:** *Cedrus deodara-Pinus wallichiana-Isodon rugosus* and **PCP:** *Pinus wallichiana-Cedrus deodara- Parrotiopsis jacquemontiana.*

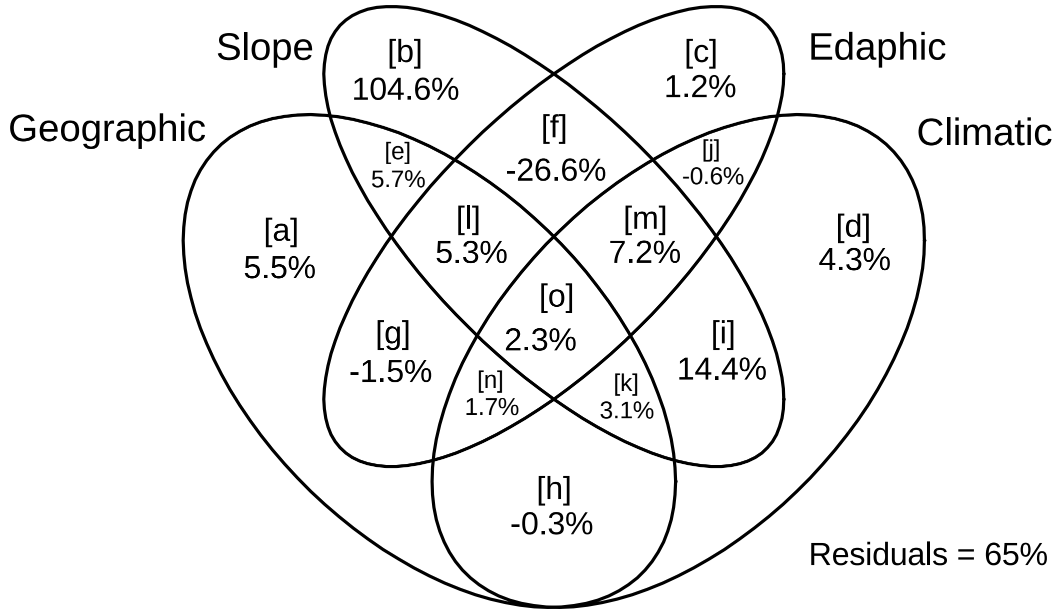

Figure 5. Venn diagram showing variation partitioning results (partial CCA) and contribution (Legendre 2008) of the four environmental variable groups studied.

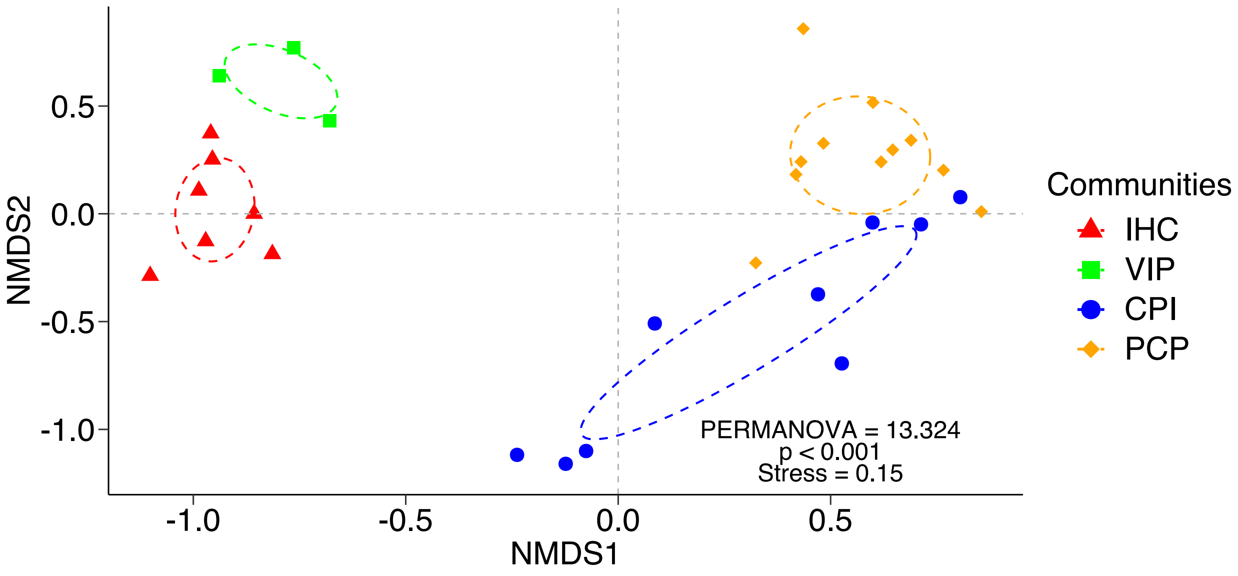

Figure 6. NMDS with PERMANOVA analysis to compare species composition between communities of moist temperate forest. **IHC**: *Indigofera heterantha- Heracleum candicans-Cynodon dactylon*, **VIP:** *Viburnum grandiflorum-Indigofera heterantha-Pinus wallichiana*, **CPI:** *Cedrus deodara-Pinus wallichiana-Isodon rugosus* and **PCP:** *Pinus wallichiana-Cedrus deodara- Parrotiopsis jacquemontiana.*

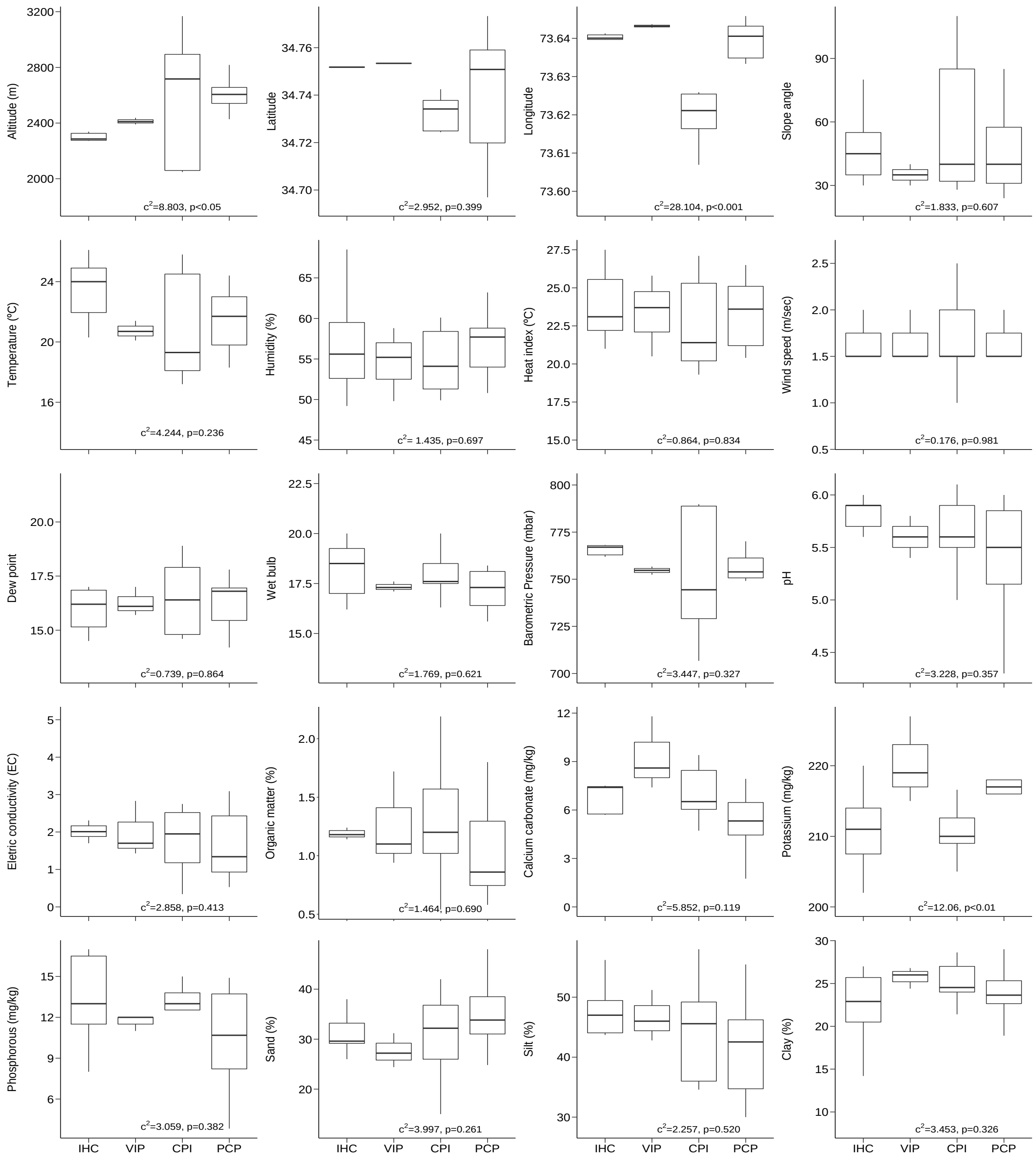

Figure 7. Variation of environmental parameters between the four plant communities of moist temperate forest, Himalaya, Pakistan. IHC: *Indigofera heterantha- Heracleum candicans-Cynodon dactylon*, **VIP:** *Viburnum grandiflorum-Indigofera heterantha-Pinus wallichiana*, **CPI:** *Cedrus deodara-Pinus wallichiana-Isodon rugosus* and **PCP:** *Pinus wallichiana-Cedrus deodara- Parrotiopsis jacquemontiana.*

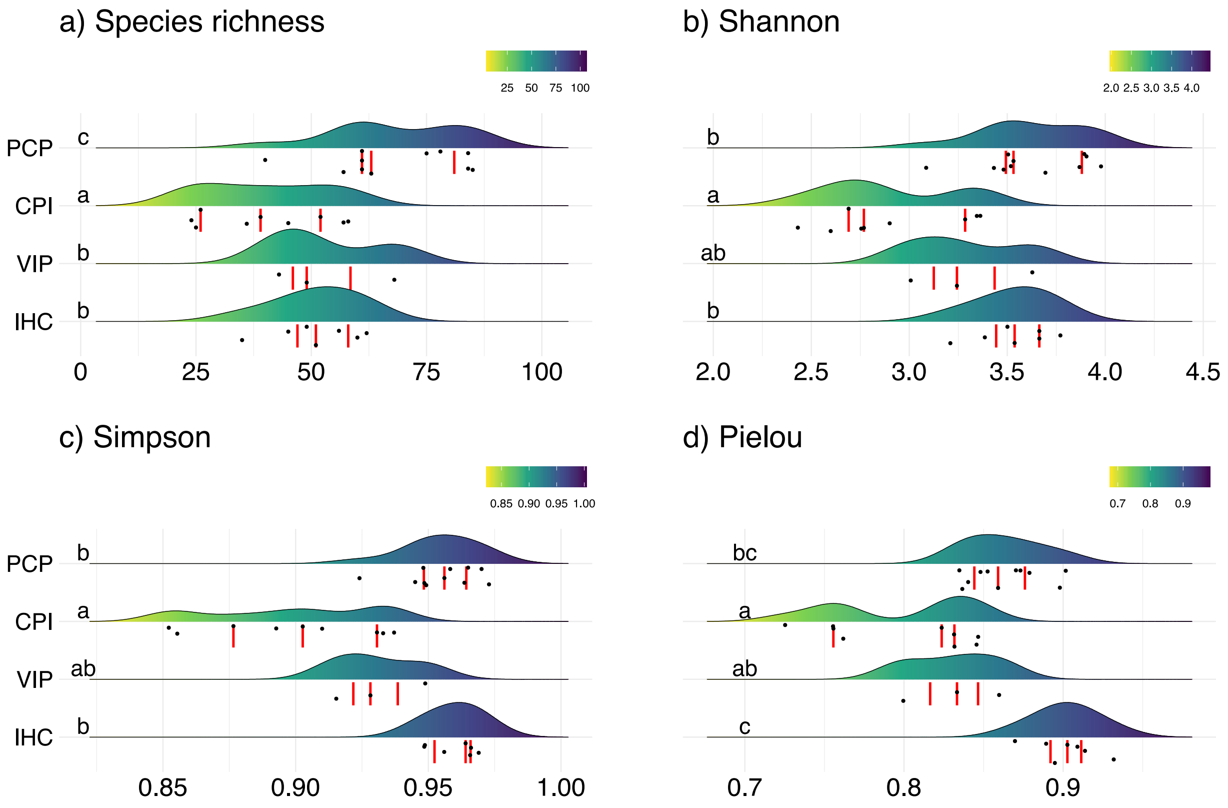

Figure 8. Variation of diversity indices between the four plant communities of moist temperate forest, Himalaya, Pakistan. Figures represent ridgeline plots with raw data (black dots below each density distributions) and first, second and third quartile (vertical red lines). Lowercase letters on the left differ from each other by estimated marginal means. Y-axis is displayed in ascendant altitudinal gradient. **IHC**: *Indigofera heterantha- Heracleum candicans-Cynodon dactylon,* **VIP:** *Viburnum grandiflorum-Indigofera heterantha-Pinus wallichiana,* **CPI:** *Cedrus deodara-Pinus wallichiana-Isodon rugosus* and **PCP:** *Pinus wallichiana-Cedrus deodara- Parrotiopsis jacquemontiana.*

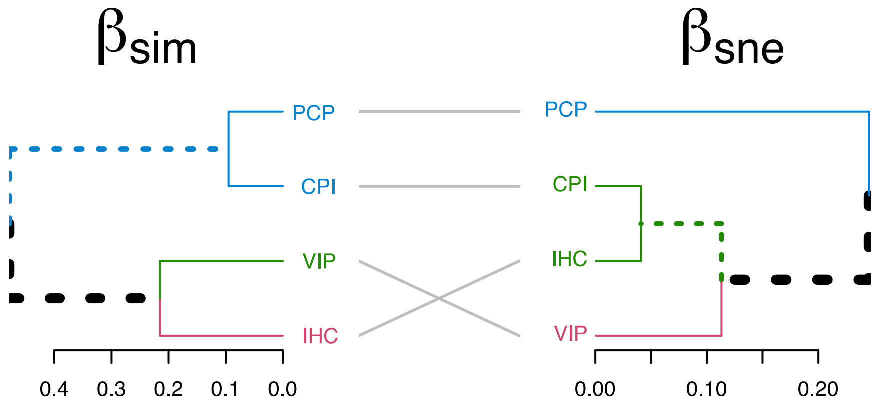

Figure 9. Dissimilarity cluster based on spatial turnover (βsim) and nestedness-resultant components (βsne) of beta diversity components of species dissimilarity between four plant communities of moist temperate forest. IHC: *Indigofera heterantha-Heracleum candicans-Cynodon dactylon*, VIP: *Viburnum grandiflorum-Indigofera heterantha-Pinus wallichiana*, CPI: *Cedrus deodara-Pinus wallichiana-Isodon rugosus*, and PCP: *Pinus wallichiana-Cedrus deodara-Parrotiopsis jacquemontiana*.

Click here to access/download
Supporting Information
Supplementary data Tables.docx

Click here to access/download
Other
Highlights.doc

Click here to access/download
Other
plos-one-change-to-authorship-form_1.docx

Click here to access/download
Other
plos-one-change-to-authorship-form_2.docx

Environmental variables drive plant species composition and distribution in the moist temperate forests of Northwestern Himalaya, Pakistan

Abstract

Questions – By assessing plant species composition and distribution in biodiversity hotspots influenced by environmental gradients, we greatly advance our understanding of the local plant community and how environmental factors are affecting these communities, which is a proxy for assessing how impacts of climate change can affect plant communities located in mountainous regions ("space-for-time" substitution). We evaluated plant species composition and distribution, and how and which environmental variables drive the plant communities in moist temperate forest of Northwestern Himalaya, Pakistan.

Location – Moist temperate zone of Manoor valley (District Mansehra, Khyber Pakhtunkhwa), Northwestern Himalayan belt of Pakistan.

Methods – During four consecutive years (2015-2018), we sampled 30 sampling sites, measuring 21 environmental variables and recording all plant species present in an altitudinal gradient range of 1932-3168 m.a.s.l. We used different multivariate analyses to identify potential plant communities, and to determine the relative importance of each environmental variable in the species composition and distribution. Finally, we also evaluated diversity patterns, by comparing diversity indices and beta diversity processes.

Results – We found that (i) the moist temperate zone in this region can be divided in four different major plant communities; (ii) each plant community has a specific set of environmental drivers; (iii) there is a significant variation in plant species composition between communities, in which six species contributed most to the plant composition dissimilarity; (iv) there is a significant difference of the four diversity indices between communities; and (v) plant

Deleted: of
Deleted: ,
Formatted: Highlight
Formatted: Highlight
Formatted: Highlight
Deleted: analyses
Deleted: analyzes
Deleted:) most of the environmental variables were significantly different between communities; (v…
Deleted: i

community structure is twice more influenced by the spatial turnover of species than by the species loss.

Conclusions – Overall, we showed that altitudinal gradients offer an important range of different environmental variables, highlighting the existence of micro-climates that drive the structure and composition of plant species in each micro-region. Each plant community along the altitudinal gradient has a set of environmental drivers, which lead to the presence of indicator species in each micro-region.

Key words: Environmental variables, plant species composition, plant species distribution, moist temperate forest, environmental gradients, hotspots of biodiversity, climate change, indicator species, multivariate approaches, Northwestern Himalaya, plant community, environmental drivers.

Formatted: Space Before: 6 pt

Formatted: Highlight

Introduction

Formatted: Font: 14 pt

Deleted: →

Mountains are the most remarkable land forms on the earth's surface with major vegetation zones based upon environmental variations [1]. They provide habitat heterogeneity based on micro-environmental variation along the altitudinal gradient [2,3], in which environmental variables (ranging from direct and indirect effects of abiotic and biotic effects) are important factors to determine the boundaries of altitudinal zones [4,5]. It is known that altitude represents a complex gradient along which many environmental factors [6] and species diversity [7,8] change concomitantly. Within this focus, plant biodiversity is strongly influenced by different environmental variables [9], in which some plant species can survive at the edge of life in high mountains all over the world [10–12].

Due to the high variation in the environmental variables along altitudinal gradients, many mountains worldwide are important hotspots of biodiversity [13–16], making up about 50% of all plant species growing in hotspots areas [17,18]. However, despite this high endemism of species greatly influenced by various environmental gradients, such as edaphic, climatic and physiographic variables [19], these areas suffer a major impact from climate change [20,21]. Plant species present in these gradients have great adaptive power [22,23]; however, the speed with which climate change is advancing might not be sufficiently achieved by plant species adaptation in these areas, leading to a strong impact on the biodiversity of these areas [24–26], ultimately leading to variations in the community structure [27].

In the face of the current climate change and considering the importance of phytosociological studies for the understanding of biodiversity and species distribution, the Himalayas represents a fundamental piece for these studies. This region is facing a marked increase in temperature [28], which is three times greater than the global average [29]. This unprecedented rise in temperature, and modification of various environmental variables as well, may lead to shifts in species composition [24–26] and variations in community structure [27]. Although there has been a considerable increase in the number of phytosociological studies in these altitudinal regions considered hotspots of biodiversity [7,19,22,23,30], there is still limited knowledge of how and which environmental variables drive the distribution and composition of plant species along altitudinal gradients in specific hotspots regions, such as the Northwestern Himalaya.

By assessing the patterns of composition and distribution of plant species in these biodiversity hotspots influenced by environmental gradients, we greatly advance our understanding of the local plant community and how environmental factors are affecting these

Deleted: ns

Formatted: Tab stops: Not at 0.59"

communities, which is a proxy for assessing how impacts of climate change can affect plant communities located in mountainous regions [31]. In this context, we evaluated plant species composition and how and which environmental variables drive the plant species distribution of moist temperate zone of the Northwestern Himalaya, Pakistan. Briefly, we assessed (i) the potential plant communities present in the moist temperate zone; (ii) which are the environmental variables that most determine plant community structure in the moist temperate zone; (iii) whether there is variation in plant species composition between plant communities and which are the species that most contributed for species composition dissimilarity; (iv) whether there is variation of diversity indices among communities; and finally (v) which is the beta diversity process that most influence plant community structure in the moist temperate zone.

Materials and methods

Study area

The present study was carried out in the moist temperate zone of Manoor valley (District Mansehra, Khyber Pakhtunkhwa), which is a mountainous valley (34.68165 N to 34.83869 N latitude, and 73.57520 E to 73.73182 E longitude Fig. 1) in the Northwestern Himalayan belt of Pakistan [32–36] along an altitudinal range of 1932-3168 m.a.s.l. Monsoon winds are the main source of precipitation as well as the primary force of controlling erosion, topography, climate and vegetation of the Northwestern Himalaya [1].

Ethics approval and consent to participate

This study was approved by the Board of Study (BOS), Committee, Department of Botany and Advanced Study Research Board (ASRB) of Hazara University, Mansehra 21300, KP, Pakistan.

Deleted: n mountains

Deleted:) the variation of environmental variables among plant communities; (v…

Deleted: i

Deleted: ¶

Formatted: Font: 14 pt

Formatted: Space Before: 6 pt

Formatted: Font: Bold, Not Italic

Formatted: Highlight

Formatted: Highlight

Formatted: Highlight

Deleted: s

Vegetation Sampling and plant identification

In different growing seasons (from March to October), we evaluated 30 stands per year, during four consecutive years, from 2015 to 2018. The line transect method (50 meters) we used for quantitative samplings [37–42], but we never repeated the same transects over years. The surveyed stands (study area) were subdivided into 30 stands and three points randomly selected were sampled within each stand along 50 meters transect (total = 90 transects). The interval distance kept between the stands was 200 meters and 100 meters between the transects. The individuals of plant species falling precisely on the line were recorded. The phytosociological attributes (*i.e.,* density, frequency, cover and their relative values, and importance value) were used for calculation of the data from each stand [43–45]. The IV was further used to rank each plant species and species with the highest IV were considered as the dominant species [43,44]. Similarly, the plant communities were named on the basis of three dominant species [46–49]. The slope angle, aspect and exposure were recorded using clinometer; and altitude, longitude and latitude were measured by geographical positioning system (GPS). Plants specimen collection, labeling, pressing and other herbarium work methodology was adopted following [50,51]. Their identification was done with the aid of Flora of Pakistan [52–54] and submitted to the Herbarium of Hazara University, Mansehra (Pakistan).

Environmental gradients

Soil samples of 200-400grams were collected from three randomly selected transects (0-30cm depth) within each sampling stand of the studied vegetation area [55,56]. The replicated samples collected from each stand were thoroughly mixed to make a composite sample [57], stored in a sterile polythene bag and labeled with a permanent marker. Raw materials like pieces of rocks

Formatted: Font: Bold, Not Italic

Formatted: Highlight

Deleted: In each stand we used t

Formatted: Highlight

Formatted: Highlight

Formatted: Highlight

Formatted: Highlight

Formatted: Highlight

Formatted: Highlight

Deleted: index

Formatted: Font: Times New Roman, Highlight

Formatted: Font: Times New Roman, Highlight

Formatted: Font: Times New Roman, Highlight

Formatted: Font: Times New Roman

Formatted: Font: Times New Roman, Highlight

Formatted: Font: Times New Roman, Highlight

Formatted: Font: Times New Roman, Highlight

Formatted: Font: Times New Roman

Formatted: Font: Bold, Not Italic

Deleted: Physicochemical analysesanalyzes [51,52] like soil texture (*i.e.,* clay, sand, silt, loam), pH [53], electrical conductivity (EC) [54], organic matter (OM) [55], nitrogen (N), phosphorus (P), potassium (K) and calcium carbonate ($CaCO_3$) concentrations were determined for each soil sample [56–58]. …

Formatted: Highlight

Formatted: Highlight

Formatted: Highlight

Formatted: Highlight

Formatted: Highlight

and stones were removed by sieving and then the samples were shade dried. Physicochemical analyses [57,58] like soil texture (*i.e.,* clay, sand, silt, loam), pH [59], electrical conductivity (EC) [60], organic matter (OM) [61], nitrogen (N), phosphorus (P), potassium (K) and calcium carbonate ($CaCO_3$) concentrations were determined for each soil sample [55,56,62]. Moreover, other climatic gradients (*i.e.,* barometric pressure, dew point, humidity, heat index, temperature, wet bulb and wind speed) were determined using a small remote weather station (Kestrel weather tracker 4000) to record the data at each transect and then average values were calculated at stand level [19].

Statistical analyses

All the collected data of plant species, edaphic and other environmental gradients data were prepared to find out the relationship among them [63,64]. Matrixes of IV data of all the recorded plant species (244 species) from 30 sampling sites were used in the analyses. Analyses were conducted on PC-ORD [65] and R software 4.0.0 [66]. Packages used in R software are mentioned in each analysis. A georeferenced map was prepared to show the distribution pattern of distinct plant communities (Fig. 1).

Sampling effort and Cluster analyses. Species area curves (SAC) were used to check the efficiency of the sampling effort, where plant abundance data with Sørensen distance values were used to create species area curves [67]. For classification of the recorded plant species (244) and 30 stands into different plant communities, we used three different cluster analyses: Two-way Indicator Species Analysis (TWINSPAN), Cluster Analysis (CA), and Two-Way Cluster Analysis (TWCA). We identified and classified plant species and stands into major plant communities [68], as well as assess the effects of various factors (such as environmental variables) on such communities by processing clustering method using TWINSPAN [19,69].

Deleted: *i.e.*
Formatted: Highlight
Deleted: with aid of handheld weather station
Formatted: Font: Bold, Not Italic
Deleted: analyses
Deleted: analyzes
Formatted: Font: Bold, Not Italic
Deleted: →
Formatted: Font: Times New Roman, Highlight
Formatted: Font: Times New Roman, Highlight
Formatted: Font: Times New Roman, Highlight
Formatted: Font: Times New Roman, Highlight
Formatted: Font: Times New Roman, Highlight
Deleted: Analyzes
Deleted: in
Formatted: Highlight
Formatted: Font: Bold, Not Italic
Formatted: Indent: First line: 0.25"
Deleted: analyses
Deleted: analyzes
Deleted: ¶
Formatted: Font: Not Italic, Highlight
Formatted: Font: Not Italic, Highlight
Formatted: Font: Not Italic, Highlight
Formatted: Font: Not Italic, Highlight
Formatted: Font: Not Italic, Highlight
Deleted: potential major plant communities
Deleted: two way indicator species analysis (
Deleted:)
Deleted: With TWINSPAN we are able to identify and separate species and stands in specific plant communities, and evaluate the impacts of different factors (e.g., environmental variables) among communities.…

Plant communities and associated environmental variables. Both species and stands (sampling sites) were constrained in relation with the environmental gradients [70,71], which were divided into geographic, slope aspect, edaphic and climatic gradients. We used non-metric multidimensional scaling (NMDS) and Principal Component Analysis (PCA) ordination biplot to determine the relationship between vegetation communities and environmental gradients using the "vegan" package. In NMDS and PCA, arrows represent the environmental gradients, in which the length shows the strength of the gradient, and the direction represent the degree of correlation. The direction of gradients on the same axis reveals a positive correlation.

In addition, we performed canonical correspondence analysis (CCA) and variation partitioning tests (partial CCA) to observe how explanatory variables (climatic, edaphic, geographic, and slope) drive the plant species distribution. First we built the best model with the lowest number of variables (those that most explain variance), through the *step* function with permutation using the "stats" package [66]. Next, we also evaluated multicollinearity between variables of the final model using Variance Inflation Factor (VIF), and we removed any variable with VIF>10, one at a time. Finally, with the final model, we carried out CCA and partial CCA to check how much each group of variables (geographic, edaphic, climatic, slope) explain in our model [72]. For these analysis, we used the "vegan" package [73].

Variation of plant species composition among plant communities. To compare whether there is difference in species composition between plant communities, we also used NMDS followed by a Permutational Multivariate Analysis of Variance (PERMANOVA) with Euclidean distance and 999 permutations. After PERMANOVA, we conducted pairwise comparisons between communities with corrections for multiple testing also using Euclidean distance and

Formatted: Font: Not Italic, Font color: Auto
Formatted: Heading 2, Indent: First line: 0.25", Adjust space between Latin and Asian text, Adjust space between Asian text and numbers
Deleted: ¶
Formatted: Font: Times New Roman, Not Bold, Highlight
Formatted: Font: Not Bold
Deleted: "vegan"
Formatted: Font: Not Bold, Highlight
Formatted: Font: Not Bold
Formatted: Highlight
Deleted: "vegan"
Formatted: Font: Bold, Not Italic
Formatted: Indent: First line: 0.25"
Deleted: between
Formatted: Font: Bold, Not Italic
Deleted: ¶

999 permutations. We used false discovery rate (FDR) as p-value adjustment method. PERMANOVA and pairwise comparisons were conducted with “RVAideMemoire” package [74]. To observe the contribution of each plant species to overall dissimilarities, we used a similarity percentage analysis based on the decomposition of Bray-Curtis dissimilarity using the package “vegan” [73].

Variation of environmental variables among plant communities. To compare the 21 environmental parameters evaluated among communities, we conducted a Generalized Linear Model (GLM) with Gaussian error distribution followed by Likelihood Ratio test using the ‘stats’ and ‘car’ [75] packages, respectively.

Formatted: Font: Bold, Not Italic

Deleted: ¶

Variation of diversity indices among plant communities. To compare the diversity indices evaluated (species richness, Shannon, Simpson, and Pielou) among the four communities, we also conducted a GLM with Gaussian error distribution, except for species richness, in which we used Poisson distribution followed by Likelihood Ratio test. Pairwise comparisons were conducted with estimated marginal means using the package ‘emmeans’ [76].

Deleted: ¶

Formatted: Font: Bold, Not Italic

Beta diversity. To evaluate which is the beta diversity process that most influence plant community structure in the moist temperate zone, we decomposed the Sørensen dissimilarity index (βsor), a measure of overall species replacement into two additive components: the spatial turnover (Simpson pairwise dissimilarity, βsim) and nestedness-resultant components (nestedness-fraction of Sorensen pairwise dissimilarity, βsne) [77–79]. Dissimilarity analysis was conducted in the package “betapart” [80].

Formatted: Font: Bold, Not Italic

Deleted: ¶

Results

Sampling effort and Cluster analyses

Deleted: analyses

Deleted: analyzes

Formatted: Font: Bold, Not Italic

Formatted: Font: Bold, Not Italic

A total of 244 plant species belonging to 194 genera and 74 families (Table S1) were recorded in moist temperate forest of Manoor valley, Northwestern Himalaya, Pakistan. The moist temperate forest ranged from 1932.3m to 3168m. SAC analysis showed that the maximum number of plant species appeared up to stand 26 and the species curve became parallel after it, as no new species were recorded further (Fig. 2). Based on the TWINSPAN analysis (high cluster heterogeneity value – Lambda = 0.4045), we identified four different major plant communities (Fig. 3), which were composed of different indicator species (IHC: *Indigofera heterantha-Heracleum candicans-Cynodon dactylon;* VIP: *Viburnum grandiflorum-Indigofera heterantha-Pinus wallichiana,* CPI: *Cedrus deodara-Pinus wallichiana-Isodon rugosus*, and PCP: *Pinus wallichiana-Cedrus deodara- Parrotiopsis jacquemontiana*).

The IHC community was primarily found in the lower mountainous ranges (1932.3-2338.4 m.a.s.l), where the dominating flora was a combination of shrub and herb species owing to the existence of a substantial herbaceous layer of *Cynodon dactylon*, which carpeted the landscape alongside *Indigofera heterantha* patches. The VIP community was recognized mainly in the foothills and adjacent plains (2390.5-2437.8 m.a.s.l), where the predominant vegetation was shrubland with abundant patches of *Viburnum grandiflorum* and *Indigofera heterantha*, accompanied by the co-dominant *Pinus wallichiana* (tree species). Nonetheless, the other two plant communities, i.e., PCP and CPI, were significantly dominated by the tree species layer at the middle (2292-2947 m.a.s.l) and higher (2048.2-3168 m.a.s.l) altitudinal ranges alongside shrubby associates.

Plant communities and associated environmental variables

Correlation between moist temperate forest plant communities and environmental gradients were illustrated through NMDS (Fig. 4a-d) and PCA (Fig. 4e). Communities were related to specific

Deleted: →
Formatted: Highlight
Formatted: Highlight
Formatted: Highlight
Deleted: our
Formatted: Font: Times New Roman, 12 pt, Highlight
Formatted: Font: Times New Roman, 12 pt, Highlight
Formatted: Font: Times New Roman, 12 pt, Highlight
Formatted: Indent: First line: 0.5", Line spacing: Double
Formatted: Font: Times New Roman, 12 pt, Highlight
Formatted: Font: Times New Roman, 12 pt, Italic, Highlight
Formatted: Font: Times New Roman, 12 pt, Highlight
Formatted: Font: Times New Roman, 12 pt, Italic, Highlight
Formatted: Font: Times New Roman, 12 pt, Highlight
Formatted: Highlight
Formatted: Highlight
Formatted: Highlight
Formatted: Font: Italic, Highlight
Formatted: Highlight
Formatted: Font: Italic, Highlight
Formatted: Highlight
Formatted: Highlight
Formatted: Font: Italic, Highlight
Formatted: Highlight
Formatted: Highlight
Formatted: Highlight
Formatted: Highlight
Formatted: Highlight
Formatted: Highlight
Formatted: Highlight
Formatted: Highlight
Deleted: ¶
Formatted: Font: Bold, Not Italic
Deleted: →

environmental variables such as geographic, slope, edaphic, and climatic variables (Table S2). Altitude, slope angle and aspects (SE, NE, ES, WN), K, pH, OM, loam, silt, sand, clay, temperature, heat index, wind speed and barometric pressure were the most representative environmental variables which drive the plant community structure and diversity along the altitudinal gradient. In both ordinations, the environmental variables separate 30 sampling sites into four major plant communities, as also showed by the TWINSPAN method (Fig. 3). In constrained PCA ordination, the maximum explanatory variation was accounted on PC1 axis (20%) and lower variation on PC2 axis (14.2%). Maximum strength was recorded for the most important environmental gradient, *i.e.,* profound influence of the environmental gradients was revealed by dividing the vegetation of the moist temperate forest into four communities (Fig. 4e), as also showed by TWINSPAN and NMDS.

The PCP community showed positive and significant correlation with northern aspect, silty loamy soil texture, humidity and altitude (Fig. 4b-c). In contrast, CPI community showed positively significance with southern slope, wind speed, dew point and wet bulb. IHC community showed positively significance with silty soil texture, electric conductivity and pH. And finally, VPI community revealed positively significant correlation with north-western slope, $CaCO_3$ and pH. Thus, all the four communities were found separately in clumps with clear differences based on the environmental gradients (Fig. 4).

The CCA and variation partitioning tests showed that the total inertia results of CCA was 3.023, where our final variables (Altitude, Temperature, Humidity, Wind speed, Slope Angle, Slope N, Slope NW, Slope SW, pH, EC, OM, $CaCO_3$, K, P, Sand, and Loam) together explained 66.5% of variation (sum of canonical eigenvalues was 2.011). The first two canonical axes explained 37.1% of variation. CCA model was significant (χ^2=2.010; pseudo-F value=1.613;

p<0.001; df=16; permutations=999). For the 16 explanatory variables, we tested simple term effects. Simple term effects showed that Altitude, Slope SW, Slope NW, Slope N, Slope Angle, K, and Humidity (decreasing order of importance) were significant (p<0.05; Table 1). The 16 explanatory variables were grouped into four classes: Climatic (Humidity, Temperature, Wind speed); Edaphic (pH, EC, OM, $CaCO_3$, K, P, Sand, Loam); Geographic (Altitude); and Slope (Slope Angle, Slope N, Slope NW, Slope SW), and then, we performed variation partitioning tests (partial CCA) for all 15 possible classes (Table 2). Class [b] was the most explanatory variable (104.6%) followed by class [m] (7.2%) (Fig. 5).

Variation of plant species composition among plant communities

We found a significant variation in plant species composition between communities (Table 3; Fig. 6), in which all communities showed a significant difference in species composition between each other (Table 4). Out of 244 species, six species greatly contributed to the variation in plant species composition between communities, namely *Viburnum grandiflorum*, *Indigofera heterantha*, *Heracleum candicans*, *Cedrus deodara*, *Pinus wallichiana*, and *Parrotiopsis jacquemontiana* (Table 4). Overall, the three species that most contributed for the variation in species composition between communities showed 13.7-29.7 % of cumulative contribution (Table 4).

Variation of environmental variables among plant communities

GLM analyses of 21 studied variables distributed in different groups (geographic, slope, climatic and edaphic gradients) between four plant communities showed that most of the environmental variables were significantly different between communities (p-value<0.05; Fig. 7). Nonetheless, slope angle, heat index, temperature, wind speed, dew point, wet bulb, pH, organic matter, electric conductivity, potassium and phosphorous did not show significant difference.

Deleted: between
Formatted: Font: Bold, Not Italic
Deleted: →
Formatted: Font: Bold, Not Italic
Deleted: →
Deleted: analyses
Deleted: analyzes

Variation of diversity indices among plant communities

Formatted: Font: Bold, Not Italic

We found a significant difference of four diversity indices, species richness (GLM χ^2=73.113, df=3, p<0.001; Fig. 8a), Shannon (GLM χ^2=35.797, df=3, p<0.001; Fig. 8b), Simpson (GLM χ^2=46.465, df=3, p<0.001; Fig. 8c), and Pielou (GLM χ^2=44.093, df=3, p<0.001; Fig. 8d), between the four communities. PCP showed the highest average number of species (68.1±4.2; mean±SE) followed by VIP (53.3±7.5) and IHC (51.1±3.5), and finally by CPI, with the lowest number of species (40.2±4.5) (Fig. 8a). PCP showed a Shannon' value of 3.62±0.08 (mean±SE), followed by IHC (3.53±0.07), VIP (3.29±0.1), and CPI (2.9±0.1) respectively (Fig. 8b). IHC showed the highest Simpson' value (0.959±0.01; mean±SE), followed by PCP (0.954±0.01), VIP (0.930±0.01), and CPI (0.898±0.01) respectively (Fig. 8c). Finally, IHC showed the highest Pielou' value (0.901±0.01; mean±SE), followed by PCP (0.862±0.01), VIP (0.830±0.01), and CPI (0.797±0.01) respectively (Fig. 8d).

Deleted: →

Beta diversity

Formatted: Font: Bold, Not Italic

The total beta diversity (βsor) showed a value of 54.7% dissimilarity, of which spatial turnover (βsim) made up 40.5% and nestedness-resultant components (βsne) made up 14.2%. In βsim cluster, we observed 47.8% dissimilarity between PCP-CPI cluster and VIP-IHC cluster (Fig. 9). PCP showed a dissimilarity of 9.4% with CPI, and VIP showed a dissimilarity of 21.5% with IHC (Fig. 9). In βsne cluster, we found 24.5% dissimilarity between PCP and VIP-CPI-IHC cluster (Fig. 9). VIP showed a dissimilarity of 11.3% with IHC-CPI, and IHC had 4.1% dissimilarity with CPI (Fig. 9). Thus, plant community structure is twice more influenced by the spatial turnover of species (βsim) than by the species loss (nestedness-resultant, βsne).

Deleted: →

Discussion

Formatted: Font: 14 pt

Mountain ecosystems are characterized by dramatic changes in temperature and abiotic properties over short altitudinal and geographical distances, making them ideal natural laboratories for studying vegetation response to environmental changes [81]. In this study, we evaluated the plant species composition and distribution in a hotspot of biodiversity, the Northwestern Himalayan mountains, Pakistan, assessing how environmental gradients, source of habitat heterogeneity, influence plant community structure and diversity, which might be a proxy for assessing how climate change impacts on plant communities located in mountainous regions [31,82,83]. We found that (i) the moist temperate zone in this region can be divided in four different major plant communities; (ii) each plant community has a specific set of environmental drivers; (iii) there is a significant variation in plant species composition between communities, in which six species contributed most to the plant composition dissimilarity; (iv) there is a significant difference of the four diversity indices (species richness, Shannon, Simpson, Pielou) between communities; and finally (v) plant community structure is twice more influenced by the spatial turnover of species (βsim) than by the species loss (nestedness-resultant, βsne). Overall, we showed that altitudinal gradients offer an important range of different environmental variables, highlighting the existence of micro-climates that drive the structure and composition of plant species in each micro-region. In addition, each plant community along the altitudinal gradient has a set of environmental drivers, which lead to the presence of indicator species in each micro-region.

Mountain plant communities are thought to be sensitive to climate change and, thus, able to reveal its effects sooner than others [31,84]. The four communities found showed a wide range of environmental drivers; however, altitude and temperature showed great prominence, probably making up the main environmental drivers in mountainous plant communities. Similar pattern

Deleted: →

Formatted: Highlight

Deleted: v) most of the environmental variables were significantly different between communities; (…

Deleted: i

was observed in the allied area (Nandiar catchment, Battagram) of Northwestern Himalaya by stating altitude and temperature as the governing gradient [69]. Such variables, which can be strongly correlated [85], modify the diversity and structure of plant communities by creating local micro-climates [86], directly influencing plant community composition and diversity [19,26,87,88].

Indeed, there is no order of importance of environmental variables, but studies are unanimous in showing that there is a consensus on the explanations for the variables' influences. For instance, in two recent studies we showed that the altitude-temperature relationship significantly influenced the physiological attributes of some plant species in the Northwestern Himalayan region [22,23], which can be a proxy for understanding plant adaptation to climate change. Any change in soil parameters has a significant effect on the growth of plant communities [19]. The studies on mountain forest habitats around the world have also revealed the role of soil structure on species zonation [67,89,90]. Furthermore, both chemical and physical attributes of the soil are related to natural soil characteristics, with an impact on plant species composition and distribution of higher vascular plants [91–93]. For instance, some soil variables can have great influence on plant composition and distribution, such as pH. Some studies have shown that pH level on soil can influence nutrient availability, ultimately influencing nutrient uptake for growth [94–96]. However, the availability of some nutrients as a result of pH levels can be detrimental for some plants, since some nutrients are toxic to some plants [94,97]. Considering that there is a great variability of pH levels and nutrient availability and concentration along altitudinal gradients, it is expected a great variability of plant species composition which can be more or less related to specific soil parameters. Since plants are sensitive to small variations of soil characteristics such as pH, minerals, organic matter, among

Deleted: n

Deleted: region

Deleted: ¶

Deleted:

Formatted: Highlight

others, and these variables are constantly changing along altitudinal gradients directly and indirectly influencing the presence and availability of other organisms and resources, some plant species might have adapted to specific set of variables.

Variability in plant species diversity is an outcome of species interaction with particular set of environment variables either abiotic and biotic [98,99], which can occur in both space and time [100,101]. The concept of changing species composition and vegetation continuum along the ecological gradients emerged as an antithesis model for distinct units [102,103]. In our study, the moist temperate forest of the studied Northwestern Himalayan region is comprised in an altitudinal gradient of approximately 1500 m. This gradient is subject to strong micro-climatic variation, which results in a set of micro-regions (better discussed above). Each micro-region has certain characteristics, which will influence the set of species that will inhabit these spaces [24–26]. In this sense, it is expected that the plant community structure is more influenced by the spatial turnover of species (βsim) than by the species loss (nestedness-resultant, βsne), *i.e.*, that there are different plant communities along the altitudinal gradient, as shown by our results. The differentiation of species diversity was mainly a consequence of environmental variables which is due to soil factors [104]. Therefore, in addition to the influence of edaphic factors in space on species composition and vegetation continuum, as shown in our study, results from similar studies have shown that the altitude is also important in driving vegetation structure and diversity in plant communities.

We found that environmental heterogeneity among plant communities have significant effects on beta diversity, particularly the spatial turnover. These results indicate that there is not a significant loss of the number of species between the plant community, but a variation in the species composition. This variation may be closely linked to the environmental effects in the

Deleted: availaility

Deleted: migh

Formatted: Font color: Auto

Deleted: ¶

Deleted: →

area, which induces the appearance of species adapted to environmental variables. The local community composition replacement implied the simultaneous loss and gain of species due to immigration–extinction dynamics and trait-based environmental filtering [105,106]. This indicates the relationship among plant community types and among species based on multiple factors. Although we did not find a large variation in βsne (loss of species between plant communities), it is important to note that temporal analysis might be important to consider a notable variation in this component of beta diversity; and βsne variations will be better observed in long-term analysis in future studies. In this sense, it is expected that the plant community structure is more influenced by the spatial turnover of species (βsim) than by the species loss (nestedness-resultant, βsne), *i.e.*, that there are different plant communities along the altitudinal gradient, as shown by our results. Similarly, results were report by Haq et al. [107] from forest of Kashmir Himalaya, India.

We observed a significant variation in plant species composition between communities, in which all communities showed a significant difference in species composition between each other. The measure of Bray-Curtis dissimilarity shows that species composition change that is influenced mainly by abundant species, in our study six species *(Viburnum grandiflorum*, *Indigofera heterantha*, *Heracleum candicans*, *Cedrus deodara*, *Pinus wallichiana*, and *Parrotiopsis jacquemontiana)* contributed most to the plant composition dissimilarity. These results suggest that the richness and turnover patterns we observed were driven primarily by rare species, which comprise most of the local species pools at these forest communities [108]. These findings are consistent with the idea that less abundant species are more sensitive to climate variability than longer lived and more abundant species [109]. The high level of turnover is

Formatted: Justified, Indent: First line: 0.44", Tab stops: Not at 0.59"

common and is an important mechanism by which a large regional species pool buffers site level diversity from interannual variation in climate [110].

Current study provides the baseline and first insights of spatial distribution, vegetation pattern and species contribution in response to environmental gradients in a moist temperate forest, Northwestern Himalaya, Pakistan. Studies that evaluate the distribution and composition of the plant community are fundamental for a better understanding of the local plant community, the conservation status and protection of these communities, as well as providing support for mitigation measures. Especially in the case of Northwestern Himalaya, which represents a biodiversity hotspot, it is even more important that we conduct phytosociological studies in these areas in order to preserve the biodiversity there. In the face of current climate changes, these regions are being heavily impacted [28,29], where the probability of species extinction may be higher than elsewhere, as these regions are rich in endemic species. Finally, we need to consider that phytosociological studies consider a general profile of the first trophic chains level, *i.e.*, to evaluate the composition, distribution and diversity of plants is to indirectly assess the first level of trophic chains.

Supporting information

S1 Table. Species composition and IV according to each stand and community found along four years of collection in moist temperate forest of Manoor valley, Himalaya, Pakistan. IHC: *Indigofera heterantha- Heracleum candicans-Cynodon dactylon*, VIP: *Viburnum grandiflorum-Indigofera heterantha-Pinus wallichiana*, CPI: *Cedrus deodara-Pinus wallichiana-Isodon rugosus* and PCP: *Pinus wallichiana-Cedrus deodara- Parrotiopsis jacquemontiana*.

S2 Table. Mean (SD) of environmental variables and plant species richness per community found along four years of collection in moist temperate forest of Manoor valley, Northwestern Himalaya.

Deleted: Himalaya

Deleted:

Deleted: take into account

Formatted: Highlight

Formatted: Line spacing: Double

Formatted: Font: Bold, Highlight

Formatted: Highlight

Formatted: Font: Not Bold, Highlight

Formatted: Highlight

Formatted: Font: Not Bold, Highlight

Formatted: Highlight

Formatted: Font: Not Bold, Highlight

Formatted: Highlight

Formatted: Font: Not Bold, Highlight

Formatted: Highlight

Formatted: Font: Not Bold, Highlight

Formatted: Highlight

Formatted: Right: 0.01", Space Before: 12 pt, Line spacing: Double

Formatted: Font: Bold, Highlight

Formatted: Highlight

Competing Interests Statement

Authors declare that there is no conflict of interest.

Acknowledgments

We thank the Higher Education Commission (HEC), Pakistan, for granting a scholarship under the International Research Support Initiative Program (IRSIP) to conduct a research work at Missouri Botanical Garden, Saint Louis, MO, USA (IUR). The authors would like to extend their sincere appreciation to the Deanship of Scientific Research at King Saud University for its funding to the Research Group number (RG-1435-014).

Deleted: Ethics approval and consent to participate:
This study was approved by the Board of Study (BOS), Committee, Department of Botany and Advanced Study Research Board (ASRB) of Hazara University, Mansehra 21300, KP, Pakistan.

Author Contributions

I.U.R: Conceptualization, Investigation, Methodology, Software, Data curation, Formal analysis, Writing- Original draft preparation. R.H: Supervision, Methodology, Writing – review & editing, Validation. A.A: Supervision, Methodology, Visualization, Validation. Z.I: Supervision, Methodology, Validation. E.S.C: Software, Writing – review & editing, Validation. E.F.A., A.A.A. A.H., and SMH.: Literature gathering, helped in discussion. F.I: Data curation, herbarium work.

References

Formatted: Font: 14 pt

1. Khan SM. Plant communities and vegetation ecosystem services in the Naran Valley, Western Himalaya. PhD Dissertation, University of Leicester, UK. 2012.
2. Luo Z, Tang S, Li C, Fang H, Hu H, Yang J, et al. Environmental effects on vertebrate species richness: testing the energy, environmental stability and habitat heterogeneity hypotheses. PLoS One. 2012/04/18. 2012;7: e35514–e35514. doi:10.1371/journal.pone.0035514

3. Yang Z, Liu X, Zhou M, Ai D, Wang G, Wang Y, et al. The effect of environmental heterogeneity on species richness depends on community position along the environmental gradient. Sci Rep. 2015;5: 15723. doi:10.1038/srep15723
4. Shipley B, Keddy PA. The individualistic and community-unit concepts as falsifiable hypotheses. Theory and models in vegetation science. Springer; 1987. pp. 47–55.
5. Frahm J-P, Gradstein SR. An altitudinal zonation of tropical rain forests using byrophytes. J Biogeogr. 1991; 669–678.
6. Givnish TJ. On the causes of gradients in tropical tree diversity. J Ecol. 1999;87: 193–210.
7. Iqbal Z, Zeb A, Abd_Allah EF, Rahman IU, Khan SM, Ali N, et al. Ecological assessment of plant communities along the edaphic and topographic gradients of biha valley, District Swat, Pakistan. Appl Ecol Environ Res. 2018;16. doi:10.15666/aeer/1605_56115631
8. Pauli H, Gottfried M, Dullinger S, Abdaladze O, Akhalkatsi M, Alonso JLB, et al. Recent Plant Diversity Changes on Europe's Mountain Summits. Science (80-). 2012;336: 353 LP – 355. doi:10.1126/science.1219033
9. Bellard C, Bertelsmeier C, Leadley P, Thuiller W, Courchamp F. Impacts of climate change on the future of biodiversity. Ecol Lett. 2012;15: 365–377.
10. Kullman L. Alpine flora dynamics – a critical review of responses to climate change in the Swedish Scandes since the early 1950s. Nord J Bot. 2010;28: 398–408. doi:10.1111/j.1756-1051.2010.00812.x
11. Jabis MD. Climate Change Impacts in Alpine Plant Communities. UC Berkeley; 2018.
12. Malanson GP, Nelson EL, Zimmerman DL, Fagre DB. Alpine plant community diversity

in species–area relations at fine scale. Arctic, Antarct Alp Res. 2020;52: 41–46. doi:10.1080/15230430.2019.1698894

13. Cañadas EM, Fenu G, Peñas J, Lorite J, Mattana E, Bacchetta G. Hotspots within hotspots: Endemic plant richness, environmental drivers, and implications for conservation. Biol Conserv. 2014;170: 282–291. doi:https://doi.org/10.1016/j.biocon.2013.12.007

14. Noroozi J, Talebi A, Doostmohammadi M, Rumpf SB, Linder HP, Schneeweiss GM. Hotspots within a global biodiversity hotspot - areas of endemism are associated with high mountain ranges. Sci Rep. 2018;8: 10345. doi:10.1038/s41598-018-28504-9

15. Kidane YO, Steinbauer MJ, Beierkuhnlein C. Dead end for endemic plant species? A biodiversity hotspot under pressure. Glob Ecol Conserv. 2019;19: e00670. doi:https://doi.org/10.1016/j.gecco.2019.e00670

16. Noroozi J, Naqinezhad A, Talebi A, Doostmohammadi M, Plutzar C, Rumpf SB, et al. Hotspots of vascular plant endemism in a global biodiversity hotspot in Southwest Asia suffer from significant conservation gaps. Biol Conserv. 2019;237: 299–307. doi:https://doi.org/10.1016/j.biocon.2019.07.005

17. Motiekaityté V. Conservation diversity of vascular plants and their communities in situ, applying the conception of ecosystem pool. Ekologija. 2006;2: 1–7.

18. Myers N, Mittermeier RA, Mittermeier CG, Fonseca GAB da, Kent J. Biodiversity hotspots for conservation priorities. Nature. 2000;403: 853–858. doi:doi.org/10.1038/35002501

19. Rahman IU, Afzal A, Iqbal Z, Bussmann RW, Alsamadany H, Calixto ES, et al. Ecological gradients hosting plant communities in Himalayan subalpine pastures:

Application of multivariate approaches to identify indicator species. Ecol Inform. 2020;60: 101162. doi:https://doi.org/10.1016/j.ecoinf.2020.101162

20. Baldwin AH, Jensen K, Schönfeldt M. Warming increases plant biomass and reduces diversity across continents, latitudes, and species migration scenarios in experimental wetland communities. Glob Chang Biol. 2014;20: 835–850.

21. Rudgers JA, Kivlin SN, Whitney KD, Price M V, Waser NM, Harte J. Responses of high-altitude graminoids and soil fungi to 20 years of experimental warming. Ecology. 2014;95: 1918–1928.

22. Rahman IU, Afzal A, Iqbal Z, Hart R, Abd_Allah EF, Alqarawi AA, et al. Response of plant physiological attributes to altitudinal gradient: Plant adaptation to temperature variation in the Himalayan region. Sci Total Environ. 2020;706: 135714. doi:10.1016/j.scitotenv.2019.135714

23. Rahman IU, Hart R, Afzal A, Iqbal Z, Alqarawi AA, Abd_Allah EF, et al. Ecophysiological plasticity and cold stress adaptation in Himalayan alpine herbs: Bistorta affnis and Sibbaldia procumbens. Plants. 2019;8: 378–386. doi:10.3390/plants8100378

24. Schuur EAG, Crummer KG, Vogel JG, Mack MC. Plant species composition and productivity following permafrost thaw and thermokarst in Alaskan tundra. Ecosystems. 2007;10: 280–292.

25. Weltzin JF, Bridgham SD, Pastor J, Chen J, Harth C. Potential effects of warming and drying on peatland plant community composition. Glob Chang Biol. 2003;9: 141–151.

26. Rahman IU, Hart R, Afzal A, Iqbal Z, Abdallah EF, Alqarawi AA, et al. Phenological plasticity in Berberis lycium Royle along temporal and altitudinal gradients. Appl Ecol Environ Res. 2019;17: 331–341. doi:10.15666/aeer/1701_331341

27. Walker MD, Wahren CH, Hollister RD, Henry GHR, Ahlquist LE, Alatalo JM, et al. Plant community responses to experimental warming across the tundra biome. Proc Natl Acad Sci. 2006;103: 1342–1346.

28. Shrestha UB, Gautam S, Bawa KS. Widespread climate change in the Himalayas and associated changes in local ecosystems. PLoS One. 2012;7: e36741. doi:10.1371/journal.pone.0036741

29. Xu J, Grumbine RE, Shrestha A, Eriksson M, Yang X, Wang Y, et al. The melting Himalayas: Cascading effects of climate change on water, biodiversity, and livelihoods. Conserv Biol. 2009;23: 520–530. doi:10.1111/j.1523-1739.2009.01237.x

30. Khan AM, Qureshi R, Saqib Z. Multivariate analyses of the vegetation of the western Himalayan forests of Muzaffarabad district, Azad Jammu and Kashmir, Pakistan. Ecol Indic. 2019;104: 723–736.

31. Niu Y, Yang S, Zhou J, Chu B, Ma S, Zhu H, et al. Vegetation distribution along mountain environmental gradient predicts shifts in plant community response to climate change in alpine meadow on the Tibetan Plateau. Sci Total Environ. 2019;650: 505–514.

32. Rahman IU, Ijaz F, Afzal A, Iqbal Z, Ali N, Khan SM. Contributions to the phytotherapies of digestive disorders: Traditional knowledge and cultural drivers of Manoor Valley, Northern Pakistan. J Ethnopharmacol. 2016;192: 30–52. doi:10.1016/j.jep.2016.06.049

33. Rahman IU, Ijaz F, Iqbal Z, Afzal A, Ali N, Afzal M, et al. A novel survey of the ethno medicinal knowledge of dental problems in Manoor Valley (Northern Himalaya), Pakistan. J Ethnopharmacol. 2016;194: 877–894. doi:10.1016/j.jep.2016.10.068

34. Rahman IU, Afzal A, Iqbal Z, Ijaz F, Ali N, Bussmann RW. Traditional and

ethnomedicinal dermatology practices in Pakistan. Clin Dermatol. 2018;36: 310–319. doi:10.1016/j.clindermatol.2018.03.016

35. Rahman IU, Afzal A, Iqbal Z, Hart R, Abd_Allah EF, Hashem A, et al. Herbal teas and drinks: Folk medicine of the Manoor valley, Lesser Himalaya, Pakistan. Plants. 2019;8: 1–18. doi:10.3390/plants8120581

36. Rahman IU, Afzal A, Iqbal Z, Ijaz F, Ali N, Asif M, et al. First insights into the floristic diversity, biological spectra and phenology of Manoor valley, Pakistan. Pakistan J Bot. 2018;50: 1113–1124.

37. Buckland ST, Anderson DR, Burnham KP, Laake JL, Borchers DL, Thomas L. Introduction to distance sampling: estimating abundance of biological populations. 2001.

38. Buckland ST, Anderson DR, Burnham KP, Laake JL, Borchers DL, Thomas L. Advanced distance sampling. Oxford University Press Oxford; 2004.

39. Buckland ST, Newman KB, Fernández C, Thomas L, Harwood J. Embedding population dynamics models in inference. Stat Sci. 2007; 44–58.

40. Anderson DR, Burnham KP, Laake JL. Distance sampling: estimating abundance of biological populations. Chapman &Hall, London, United Kingdom. 1993.

41. Sprent P. Distance Sampling-Estimating Abundance of Biological Populations. JSTOR; 1994.

42. Le Moullec M, Pedersen ÅØ, Yoccoz NG, Aanes R, Tufto J, Hansen BB. Ungulate population monitoring in an open tundra landscape: distance sampling versus total counts. Wildlife Biol. 2017;2017.

43. Curtis JT, McIntosh R. The interrelations of certain analytic and synthetic phytosociological characters. Ecology. 1950;31: 434–455.

44. Curtis JT. The vegetation of Wisconsin: an ordination of plant communities. University of Wisconsin Pres; 1959.

45. Jayasuriya AHM, Pemadasa MA. Factors affecting the distribution of tree species in a dry zone montane forest in Sri Lanka. J Ecol. 1983; 571–583.

46. Son H-J, Park S-H, Lee D-H, Song J-H, Park H-W, Park W-G. Growing environment characteristics and vegetation structure of Vaccinium Oldhamii Miq. native habitats in Korea. Forest Sci Technol. 2019;15: 33–43.

47. Tian Z, Li H, Li W, Gan X, Zhang X, Fan Z. Structural characteristics and niches of dominant tree populations in Tetracentron sinense communities: implications for conservation. Bot Sci. 2018;96: 157–167.

48. Lou Y, Pan Y, Gao C, Jiang M, Lu X, Xu YJ. Response of plant height, species richness and aboveground biomass to flooding gradient along vegetation zones in floodplain wetlands, Northeast China. PLoS One. 2016;11: e0153972.

49. Song JH, Cheong EJ, Kim H, Kim MS, Kim SH. Variation of morphological characteristics and anthocyanin contents from fruit of Vaccinium oldhamii in Korea. J Korean Soc For Sci. 2015;104: 193–197.

50. Ijaz F. Biodiversity and traditional uses of plants of Sarban Hills, Abbottabad. M.Phil Dissertation, Hazara University Manehra-21300, Khyber Pakhtunkhwa, Pakistan. 2014.

51. Ijaz F, Rahman I., Iqbal Z, Alam J, Ali N, Khan S. Ethno-ecology of the healing forests of Sarban Hills, Abbottabad, Pakistan: an economic and medicinal appraisal. 1st ed. In: M. Ozturk KRH, editor. Plant and Human Health. 1st ed. Springer International Publishing AG; 2018. pp. 675–706. doi:https://doi.org/10.1007/978-3-319-93997-1_19

52. Nasir E, Ali SI. Flora West of Pakistan. Nasir E, Ali SI, editors. Karachi: National

Herbarium; 1971.

53. Ali SI, Nasir YJ. Flora of Pakistan. Ali SI, Nasir. YJ, editors. Islamabad: Department of Botany, University of Karachi, Karachi and National Herbarium; 1989.

54. Ali SI, Qaiser M. Flora of Pakistan. Karachi: Department of Botany, University of Karachi; 1995.

55. Rahman IU, Ijaz F, Afzal A, Iqbal Z. Effect of foliar application of plant mineral nutrients on the growth and yield attributes of chickpea (Cicer arietinum L.) Under nutrient deficient soil conditions. Bangladesh J Bot. 2017;46: 111–118.

56. Rahman IU, Afzal A, Iqbal Z, Ijaz F, Khan SM, Khan SA, et al. Influence of different nutrients application in nutrient deficient soil on growth and yield of onion. Bangladesh J Bot. 2015;44: 613–619.

57. Ravindranath NH, Ostwald M. Carbon inventory methods: handbook for greenhouse gas inventory, carbon mitigation and roundwood production projects. Springer International Publishing AG; 2007.

58. Haq F, Ahmad H, Iqbal Z. Vegetation description and phytoclimatic gradients of subtropical forests of Nandiar Khuwar catchment District Battagram. Pakistan J Bot. 2015;47: 1399–1405.

59. Mc Lean EO. Soil pH and Lime requirement. 2nd ed. In: Page AL, Miller RH, Keeney DR, editors. Methods of soil analysis Part 2 Chemical and Microbiological Properties. 2nd ed. Madison, Wisconsin, USA: American Society of Agronomy, Inc. Soil Science Society of America, Inc.; 1982. pp. 199–223.

60. Wilson MJ, Bayley SE. Use of single versus multiple biotic communities as indicators of biological integrity in northern prairie wetlands. Ecol Indic. 2012;20: 187–195.

61. Nelson DW, Sommers LE. Total carbon, organic carbon, and organic matter. 5th ed. In: Sparks DL, Page AL, Helmke PA, Loeppert RH, Soltanpour PN, Tabatabai MA, et al., editors. Methods ofSoil Analysis Part 3 Chemical Method. 5th ed. Madison, WI 53711, USA: Soil Science Society of America and American Society of Agronomy; 1996. pp. 961–1010. doi:https://doi.org/10.2136/sssabookser5.3.c34

62. Soltanpour PN. Determination of nutrient availability and elemental toxicity by AB-DTPA soil test and ICPS. 16th ed. Advances in soil science. 16th ed. NY: Springer, New York; 1991. pp. 165–190. doi:https://doi.org/10.1007/978-1-4612-3144-8_3

63. Šmilauer P, Jan L. Multivariate analysis of ecological data using CANOCO. 2nd ed. Cambridge CB2 8BS, United Kingdom: Cambridge University Press; 2014. doi:https://doi.org/10.1017/CBO9781139627061

64. Mayor JR, Sanders NJ, Classen AT, Bardgett RD, Clément JC, Fajardo A, et al. Elevation alters ecosystem properties across temperate treelines globally. Nature. 2017;542: 91–95. doi:10.1038/nature21027

65. McCune B, Mefford M. PC-ORD. Multivariate Analysis of Ecological Data. Version 6. Gleneden Beach, Oregon, USA: MjM Software Design; 2011.

66. R Core Team. R: A language and environment for statistical computing. R Foundation for Statistical Computing, Vienna, Austria. 2020.

67. Rahman IU. Ecophysiological plasticity and Ethnobotanical studies in Manoor Area, Kaghan Valley, Pakistan. PhD Dissertation, Hazara University, Mansehra 21300, Khyber Pakhtunkhwa, Pakistan. 2020.

68. Hill MO. TWINSPAN: a FORTRAN program for arranging multivariate data in an ordered two-way table by classification of the individuals and attributes. Cornell Univ.

1979.

69. Haq F, Ahmad H, Iqbal Z, Alam M, Aksoy A. Multivariate approach to the classification and ordination of the forest ecosystem of Nandiar valley western Himalayas. Ecol Indic. 2017;80: 232–241. doi:10.1016/j.ecolind.2017.05.047

70. Knapp AK, Fay PA, Blair JM, Collins SL, Smith MD, Carlisle JD, et al. Rainfall variability, carbon cycling, and plant species diversity in a mesic grassland. Science (80-). 2002;298: 2202–2205. doi:10.1126/science.1076347

71. McCune B. PC-ORD: an integrated system for multivariate analysis of ecological data. Abstr Bot. 1986;10: 221–225.

72. Legendre P. Studying beta diversity: ecological variation partitioning by multiple regression and canonical analysis. J Plant Ecol. 2008;1: 3–8. doi:10.1093/jpe/rtm001

73. Oksanen J, Blanchet F, Friendly M, Kindt R, Legendre P, Minchin D, et al. vegan: Community Ecology Package (R package Version 2.5-5). Accessed; 2019.

74. Hervé M. RVAideMemoire: Testing and Plotting Procedures for Biostatistics. 2020.

75. Fox J, Weisberg S. An R companion to applied regression. California: Sage Publications; 2018.

76. Lenth R. emmeans: Estimated Marginal Means, aka Least-Squares Means. 2020.

77. Baselga A, Orme CDL. betapart: an R package for the study of beta diversity. Methods Ecol Evol. 2012;3: 808–812.

78. Baselga MA. Package ‘ betapart .’ 2013.

79. Baselga A. Partitioning the turnover and nestedness components of beta diversity. Glob Ecol Biogeogr. 2010;19: 134–143. doi:10.1111/j.1466-8238.2009.00490.x

80. Baselga A, Orme D, Villeger S, De Bortoli J, Leprieur F, Logez M, et al. Partitioning

Beta Diversity into Turnover and Nestedness Components. Cran. 2018; 1–42.

81. Anslan S, Azizi Rad M, Buckel J, Echeverria Galindo P, Kai J, Kang W, et al. Reviews and syntheses: How do abiotic and biotic processes respond to climatic variations in the Nam Co catchment (Tibetan Plateau)? Biogeosciences. 2020;17: 1261–1279.

82. McCarty JP. Ecological consequences of recent climate change. Conserv Biol. 2001;15: 320–331.

83. Hansen AJ, Neilson RP, Dale VH, Flather CH, Iverson LR, Currie DJ, et al. Global change in forests: responses of species, communities, and biomes: interactions between climate change and land use are projected to cause large shifts in biodiversity. Bioscience. 2001;51: 765–779.

84. Malanson GP, Resler LM, Butler DR, Fagre DB. Mountain plant communities: Uncertain sentinels? Prog Phys Geogr Earth Environ. 2019;43: 521–543. doi:10.1177/0309133319843873

85. Cantonati M, Gerecke R, Bertuzzi E. Springs of the Alps-sensitive ecosystems to environmental change: from biodiversity assessments to long-term studies. Hydrobiologia. 2006;562: 59–96.

86. Adair KL, Lindgreen S, Poole AM, Young LM, Bernard-Verdier M, Wardle DA, et al. Above and belowground community strategies respond to different global change drivers. Sci Rep. 2019;9: 1–11. doi:10.1038/s41598-019-39033-4

87. Conti L, de Bello F, Lepš J, Acosta ATR, Carboni M. Environmental gradients and micro-heterogeneity shape fine-scale plant community assembly on coastal dunes. J Veg Sci. 2017;28: 762–773. doi:10.1111/jvs.12533

88. Rahman I-U, Afzal A, Abd_Allah EF, Iqbal Z, Alqarawi AA, Hashem A, et al.

Composition of plant communities driven by environmental gradients in alpine pastures and cold desert of northwestern Himalaya, Pakistan. Pakistan J Bot. 2021;53: 655–664. doi:10.30848/pjb2021-2(35)

89. Rawat M, Arunachalam K, Arunachalam A, Alatalo JM, Kumar U, Simon B, et al. Relative contribution of plant traits and soil properties to the functioning of a temperate forest ecosystem in the Indian Himalayas. Catena. 2020;194: 104671.

90. Hegazy AK, El-Demerdash MA, Hosni HA. Vegetation, species diversity and floristic relations along an altitudinal gradient in south-west Saudi Arabia. J Arid Environ. 1998;38: 3–13.

91. Marini L, Scotton M, Klimek S, Isselstein J, Pecile A. Effects of local factors on plant species speciesrichness and composition of Alpine meadows. Agric Ecosyst Environ. 2007;119.

92. Oduor C, Karanja N, Onwonga R, Mureithi S, Pelster D, Nyberg G. Enhancing soil organic carbon, particulate organic carbon and microbial biomass in semi-arid rangeland using pasture enclosures. BMC Ecol. 2018;18.

93. Yang Y, et al. Soil physicochemical properties and vegetation structure along an elevation gradient and implications for the response of alpine plant development to climate change on the northern slopes of the Qilian Mountains. J Mt Res. 2018;15: 1006–1019.

94. Vicherová E, Hájek M, Hájek T. Calcium intolerance of fen mosses: physiological evidence, effects of nutrient availability and successional drivers. Perspect Plant Ecol Evol Syst. 2015;17: 347–359.

95. Audorff V, Kapfer J, Beierkuhnlein C. The role of hydrological and spatial factors for the vegetation of Central European springs. J Limnol. 2011;70: 9–22.

96. Strohbach M, Audorff V, Beierkuhnlein C. Drivers of plant species composition in siliceous spring ecosystems: groundwater chemistry, catchment traits or spatial factors? J Limnol. 2009;68: 375–384.

97. Tyler T, Olsson PA. Substrate pH ranges of south Swedish bryophytes—Identifying critical pH values and richness patterns. Flora. 2016;223: 74–82.

98. Ricklefs RE. Evolutionary diversification and the origin of the diversity–environment relationship. Ecology. 2006;87: S3–S13. doi:10.1890/0012-9658(2006)87[3:EDATOO]2.0.CO;2

99. Gauthray-Guyénet V, Schneider R, Paré D, Achim A, Loi C, Sirois L. Influence of shifts over an 80-year period in forest composition on soil properties. Plant Soil. 2018;433: 111–125.

100. Suding KN, Lavorel S, Chapin FS, Cornelissen JHC, Díaz S, Garnier E, et al. Scaling environmental change through the community-level: A trait-based response-and-effect framework for plants. Glob Chang Biol. 2008;14: 1125–1140. doi:10.1111/j.1365-2486.2008.01557.x

101. Nicotra AB, Atkin OK, Bonser SP, Davidson AM, Finnegan EJ, Mathesius U, et al. Plant phenotypic plasticity in a changing climate. Trends Plant Sci. 2010;15: 684–692. doi:10.1016/j.tplants.2010.09.008

102. Vleminckx J, Schimann H, Decaëns T, Fichaux M, Vedel V, Jaouen G, et al. Coordinated community structure among trees, fungi and invertebrate groups in Amazonian rainforests. Sci Rep. 2019;9: 1–10. doi:10.1038/s41598-019-47595-6

103. Hu A, Wang J, Lu X, Zhou J. Mountain biodiversity and ecosystem functions : interplay between geology and contemporary environments. ISME J. 2019. doi:10.1038/s41396-

019-0574-x

104. Kang S, Niu J, Zhang Q, Zhang X, Han G, Zhao M. Niche differentiation is the underlying mechanism maintaining the relationship between community diversity and stability under grazing pressure. Glob Ecol Conserv. 2020;24: e01246.

105. Matthews TJ, Whittaker RJ. Neutral theory and the species abundance distribution: recent developments and prospects for unifying niche and neutral perspectives. Ecol Evol. 2014;4: 2263–2277.

106. Filgueiras BKC, Peres CA, Melo FPL, Leal IR, Tabarelli M. Winner–Loser Species Replacements in Human-Modified Landscapes. Trends Ecol Evol. 2021.

107. Haq SM, Calixto ES, Kumar M. Assessing Biodiversity and Productivity over a Small-scale Gradient in the Protected Forests of Indian Western Himalayas. J Sustain For. 2021;40: 675–694. doi:10.1080/10549811.2020.1803918

108. Cleland EE, Collins SL, Dickson TL, Farrer EC, Gross KL, Gherardi LA, et al. Sensitivity of grassland plant community composition to spatial vs. temporal variation in precipitation. Ecology. 2013;94: 1687–1696.

109. Bisbing SM, Urza AK, Buma BJ, Cooper DJ, Matocq M, Angert AL. Can long-lived species keep pace with climate change? Evidence of local persistence potential in a widespread conifer. Divers Distrib. 2021;27: 296–312.

110. Burrows MT, Hawkins SJ, Moore JJ, Adams L, Sugden H, Firth L, et al. Global-scale species distributions predict temperature-related changes in species composition of rocky shore communities in Britain. Glob Chang Biol. 2020;26: 2093–2105.

Table 1

The contribution and ranking of the studied variables in our partial CCA model. Significant variables are displayed in bold.

Variables	Df	ChiSquare	F	p-value
Altitude	1	0.251	3.229	**0.001**
Slope.SW	1	0.245	3.146	**0.001**
Slope.NW	1	0.178	2.297	**0.001**
Slope.N	1	0.214	2.748	**0.002**
Slope.Angle	1	0.163	2.097	**0.009**
K	1	0.140	1.798	**0.018**
Humidity	1	0.119	1.538	**0.039**
Wind.speed	1	0.114	1.472	0.069
CaCO3	1	0.098	1.270	0.151
Temp	1	0.081	1.049	0.376
OM	1	0.079	1.017	0.401
EC	1	0.076	0.976	0.474
P	1	0.070	0.907	0.578
Sand	1	0.069	0.895	0.590
Loam	1	0.056	0.724	0.864
pH	1	0.050	0.647	0.922
P	1	0.088	0.7295	0.820
K	1	0.075	0.6191	0.922

Deleted: Page Break

Table 2

Results of variation partitioning (partial CCA) of four environmental variable groups studied (see Fig. 5 for individual fraction letters code).

**Individual Fraction**	**Adjusted R^2**	**Variation explained (%)**	**% of all**	**Df**
[a]	0.020	5.5	0.1	1
[b]	0.370	104.6	2.3	4
[c]	0.004	1.2	0.0	8
[d]	0.015	4.3	0.1	3
[e]	0.020	5.7	0.1	0
[f]	-0.091	-25.6	-0.6	0
[g]	-0.005	-1.5	0.0	0
[h]	-0.001	-0.3	0.0	0
[i]	-0.045	-12.9	-0.3	0
[j]	-0.002	-0.6	0.0	0
[k]	0.011	3.1	0.1	0
[l]	0.019	5.3	0.1	0
[m]	0.026	7.2	0.2	0
[n]	0.006	1.7	0.0	0
[o]	0.008	2.3	0.1	0
Total explained	0.354	100.0	2.2	18
All variation	15.835	/	100	

Table 3

PERMANOVA results comparing species composition between the four communities found in Moist temperate forest. This analysis was made with Euclidean distance and 999 permutations. Pairwise comparisons between communities are depicted in Table 4.

	Df	Sums of Sqs	Mean Sqs	F	R^2	Pr(>F)
Communities	3	9961.1	3320.4	13.324	0.6059	0.001
Residuals	26	6479.1	249.2		0.3941	
Total	29	16440.2			1	

Table 4

Pairwise comparisons with FDR p-value adjustment method of species composition and contrast results of the contribution of individual species to the overall Bray-Curtis dissimilarity of species composition between the four communities found in moist temperate forest. We displayed only the three species that most contributed. Av. dis. – Average dissimilarity; SD – Standard deviation; Av Com1 – Average Community 1; Av Com2 – Average community 2; Cum. – Cumulative; Cont. – Contribution. Vib.gra**:** *Viburnum grandiflorum*, Ind.het**:** *Indigofera heterantha*, Her.can**:** *Heracleum candicans*, Ced.deo**:** *Cedrus deodara*, Pin.wal**:** *Pinus wallichiana*, Par.Jac**:** *Parrotiopsis jacquemontiana*.

**Communities**	***P-value***	**Species**	**Av dis**	**SD**	**Ratio**	**Av Com1**	**Av Com 2**	**Cum**	**Cum %**	**Cont %**
**IHC-VIP**	0.011	Vib.gra	0.1	0	5	0	24.4	0.1	12.2	12.2
		Ind.het	0.1	0	1.9	8.8	23.5	0.2	20.1	7.9
		Her.can	0	0	1.3	6.9	1.8	0.2	22.8	2.7
**IHC-CPI**	0.002	Ced.deo	0.1	0	3.8	0	22.5	0.1	10.9	10.9
		Pin.wal	0.1	0	5.3	0	20.3	0.2	20.8	9.9
		Ind.het	0	0	0.9	8.8	2.3	0.2	24.2	3.5
**IHC-PCP**	0.002	Pin.wal	0.1	0	2.7	0	16.2	0.1	6.9	6.9
		Ced.deo	0.1	0	3	0	16.1	0.1	13.8	6.9
		Par.jac	0	0	5.8	0.4	10.3	0.2	18	4.2
**VIP-CPI**	0.011	Vib.gra	0.1	0	5.1	24.4	0	0.1	11.6	11.6
		Ind.het	0.1	0	5.3	23.5	2.3	0.2	21.5	9.9
		Ced.deo	0.1	0	1.9	5.2	22.5	0.3	29.7	8.3
**VIP-PCP**	0.006	Vib.gra	0.1	0	3.3	24.4	1.4	0.1	9.5	9.5
		Ind.het	0.1	0	6.6	23.5	2.8	0.2	17.9	8.4
		Pin.wal	0	0	2.6	5.3	16.2	0.2	22.8	4.9
**CPI-PCP**	0.002	Ced.deo	0	0	1.2	22.5	16.1	0.1	5.4	5.4
		Par.jac	0	0	1.8	4.7	10.3	0.1	9.6	4.2
		Pin.wal	0	0	1.1	20.3	16.2	0.1	13.7	4

Formatted Table

Deleted: ¶

Manuscript ID # PONE-D-21-11989

Dear Editor,

We thank you and Reviewers for their constructive criticism and are pleased to resubmit a revised and improved version of our manuscript (ID # PONE-D-21-11989) entitled “”. We carefully responded to each comment in the revised version. In the response letter below, we have provided a detailed explanation of the changes made following the line numbers (see line numbers in track version).

Sincerely yours,

Inayat Ur Rahman, PhD

Revision notes

Reviewer's comments black font; author answers in blue font; responses are numbered (R#1, R#2, etc. for reference)

Reviewer(s)' Comments to Author:

Reviewer #1

General comments:
In this research paper, the authors studied influence of environmental variables in shaping plant species composition and their distribution in the moist temperate forests of Northwestern Himalaya, Pakistan. Covering 30 sampling sites, they measured 21 environmental variables for four consecutive years. Generated data is analysed using different multivariate analyses in order to identify potential plant communities and influence of environmental variable in species composition, distribution and diversity patterns. Overall the present research is well-designed and presents interesting results of multivariate analysis in order to understand how the existence of micro-climates drive the structure and composition of plant species in studied area.
R#1 – We really appreciate the positive words.

Major issue:

1. In Conclusions (line 46-48) author stated that overall, we showed that altitudinal gradients offer an important range of different environmental variables, highlighting the existence of micro-climates that drive the structure and composition of plant species in each micro-region. However there is no mention of elevation range of study area across the manuscript.
R#2 – We now added this information in the manuscript (L.14-15,93,256-265). The altitudinal range of our data sampling ranged from 1932 to 3168 m.a.s.l.

2. Kindly add some detailed information on sampling method such as: if quadrat method is opted during the study what was the size of these used quadrats. Furthermore it will be good for readers to understand the study if information regarding the number of sampling quadrates in each transect will be mentioned in the methodology section.

   R#3 – Required information regarding the vegetation sampling has been added. "The surveyed stands (study area) were subdivided into 30 stands and three points randomly selected within each stand were sampled along 50 meters transect (total = 90 transects). The interval distance kept between the stands was 200 meters and 100 meters between transects." (L. 106-112)

3. A detailed table describing four communities (**IHC; VIP; CPI; PCP)** needs to be added highlighting species composition, environmental variables, elevation range, dominant families etc.
R#4 – We now added two new supplementary tables (Table S1 and S2) describing the communities. One table describes environmental variables and the other table the species composition for each plant community.

4. Page 9 line 177 author mentioned "A total of 244 plant species were recorded 177 in moist temperate forest of Manoor valley, Himalaya, Pakistan". Kindly add details of 244 plant species belonging to ……………genus and …….families respectively.
   R#5 – As per suggestion, the required details has been added i.e., 194 genera and 74 families. (L. 246)

Commented [EC1]: Dear, could you just mention in the text the total number of genus and families?

Other comments:

1. Kindly follow uniformity across the manuscript text to avoid confusion, such as spelling variations (in line 122 analyses and 123 analyzes) and such omissions needs to be checked throughout the manuscript.

   R#6 – Done. Thank you. (L. 15, 138, 145, 148, 152, 239, 322)

2. Author has used the term Northwestern Himalaya (line 23); Himalaya (line 31): Northwestern Himalaya (line 102); Himalayan mountains (line 260) etc. thus it is suggested to use term Northwestern Himalaya to indicate study site throughout the manuscript.

   R#7 – We changed to Northwestern Himalaya in the places that we are talking about the specific region of Himalayas. Other parts are related to the Himalayas in a general perspective and then we can change it. We appreciate your understanding. (L. 10, 12, 40, 71, 80, 92, 95 and onwards)

3. For wider acceptability of the manuscript it is suggested to discuss similar studies carried out in other parts of Himalaya in discussion section.
   R#8 – We added more citations about studies conducted in the Himalayan mountains. We have now discussed it in detail. We now also added more citations about plant community studies in the Himalayas. (L. 356-358, 393-395, 404-412, 424-460)

Reviewer #2

The topic is timely, and such a kind of study, especially from the underrepresented areas, is essential and requires our attention. The authors have come up with clear questions and have analyzed them well.
R#9 – We really appreciate your positive words.

My main concern is with the methodology and discussion section; I see much basic information missing, especially concerning the data collection and study site; please look at each of the comments provided below. Discussion lacks proper explanation, key findings of different communities and predictor variables associated with them are interesting, but these points are not discussed well. Currently, the discussion is too general, but there is much potential to improve it. I also felt that your questions 2 and 4 explain the same points, so instead of keeping them as standalone objectives, it will be nice to combine them. In objective two results, all the four communities were found separately in clumps with apparent differences based on the environmental gradients, so I dint understand the need to again look into the variation of environmental variables among plant communities (iv). Lastly, instead of just reporting six species names, it will be good to have a table (primary or supplementary table), including all details of plant species in each community, their abundance, elevation range, etc.

R#10 – We now improved our manuscript and cordially ask you to check our responses for each question raised. Overall, we added more information in methods (L. 93, 106-112, 114-116, 123-137, 142-144, 146-148, 150-151, 154-159, 191-193) and discussion (L. 356-358, 393-395, 404-412, 424-460) sections. Following reviewer's very important suggestion, two new supplementary tables with a summary of the environmental variables and the species composition based on the importance value were also added.

L39-40: ii) each plant community has a specific set of environmental drivers & L41-42 iv) most of the environmental variables were significantly different between communities. I feel both of these sentences highlight the same points; my advice would be to combine both and write them as a single point.
R#11 – Thank you for your suggestion. We agreed and combined accordingly. (L. 22, 84, 366)

L90-91- and L94: I am highlighting this point again; there is an overlap in your questions 2 and 4
R#12 – Please see R#11 for the answer about this question. (L. 22, 84, 366)

L106 What were the growing seasons? Please mention the seasons name
R#13 – We now added the months that represent the growing seasons in the area, which were from March to October. (L. 106)

L106 Was the same stand sampled for four years, meaning four times? Please note that many important information on sampling design is missing; for example, how were the individuals counted, those falling precisely on the line? Furthermore, was the line horizontal or vertical? Was it stratified random sampling? Also, what was the distance between the two lines transect?
R#14 – Yes. We repeated the stands over years, but not the same transects inside each stand. We now added more information about it the text. The surveyed stands (study area) were subdivided into 30 stands and three points randomly selected within each stand were sampled along 50 meters transect (total = 90 transects). The interval distance kept between the stands was 200 meters and 100 meters between transects. The individuals of plant species falling precisely on the line were noted. (L. 106-112)

L116 There is no clarity on how soil samples, Ph values were collected. Was it collected at a single location per stand, and what depth, slope, well-exposed sunlit areas or not?
Ph varies a lot, even at a short distance between north and south facing slopes, so it is better to provide all the sampling details.
R#15 – Basic details regarding the data collection of vegetation and soil sampling are now mentioned in Vegetation sampling (L. 108-112, 114-116), and Environmental gradients (L. 123-137, 142-144) respectively. A supplementary table S2 has also been included to show the variability of environmental gradients among the four plant communities.

L121 At what distance was the weather station located from those 30 stands? The environmental variable varies across the elevation, and authors have also mentioned the importance of microclimates, so I am curious to know how many weather stations were installed.
The overall sampling design will become more apparent if the authors explain how the line transects and weather stations are distributed along the elevation gradient.

R#16 – We used a small remote weather station and recorded the data at transect level and then averaged it to stand level. Required details regarding vegetation sampling using line transect are incorporated into the Vegetation sampling section (M & M).

Results:
I feel that apart from the given figures, it will be good to have a table where the species names, elevation range, abundance in each community are mentioned. A comprehensive table with all these details may give a clearer picture of your findings. You may decide how much information you would want to share in the main manuscript and supplementary information.
R#17 – Thank you for your comment. We now added two supplementary tables about plant species composition and environmental variables values in each community. Since we already have many tables and figures in the manuscript, and these other tables will be so long, we are adding them as supplementary tables (S1 and S2).

Fig 3: Legend need to be rewritten, the author has mentioned four communities' names and four colors corresponding to each community, but the link between each color and community is missing. Also, what do the alphabet and various numbers indicate on the left side of the graph?
R#18 – We now updated our legend. Numbers and letter are the stands.
"Figure 3. Clustering method using two way indicator species analysis (TWINSPAN) indicating four different plant communities. IHC (red triangle): *Indigofera heterantha-Heracleum candicans-Cynodon dactylon,* VIP (blue circle): *Viburnum grandiflorum-Indigofera heterantha-Pinus wallichiana,* CPI (green square): *Cedrus deodara-Pinus wallichiana-Isodon rugosus* and PCP (yellow diamond): *Pinus wallichiana-Cedrus deodara-Parrotiopsis jacquemontiana.* Letters associated to numbers at the end of each branch of the dendrogram represent the stands evaluated.

L209: What was the intention behind considering temperature and altitude both as the predictor variables? Because along the mountain gradient these variables are highly correlated.
R#19 – In this specific analysis, we did not consider any kind of multicollinearity. We plot all variables to see the overall relationship between them and communities. However, if you check the partial CCA and partitioning analyses, you will see that we removed the colinear variables. Therefore, in the first analysis (NMDS, PCA) we show the overall relationship of all variables with each community, while in the second analysis, we show which are the variables that most explain the patterns found in our study.

Discussion: In the result, section author has emphasized their findings of four distinct communities and the environmental variables associated with each one of them. However, in the discussion, the authors have failed to discuss their unique findings in detail. I feel that the discussion section is too general and poorly explained. There is a lot of scopes to improve the discussion considering the amount of analysis carried out. By highlighting just temperature and altitude (which is definitely important from the climate change aspect), you may lose other valuable details of your study.
R#20 – We totally agree. In discussion, we provided more details about how these other variables can influence plant community. We believe that discussing each variable and its relationship to the community will extend a lot the manuscript, turning it tiring for readers. In this context, we believe that the general patterns and discussion of the main variables should be the best approach. We appreciate your comment, and we cordially ask to check the lines mentioned, where we added information about the influence of other variables in the

community structure. Some important edaphic variables like pH were considered and discussed accordingly. (L. 356-358, 393-395, 404-412, 424-460)

Reviewer #3

Comments on the manuscript entitled ‘Environmental variables drive plant species composition and distribution in the moist temperate forests of Northwestern Himalaya, Pakistan’.

The basic data collection on vegetation, soil, etc. and analysis are completely lacking. Overall, the environmental observation of the study is very less (4 years, 2015-2018) to draw proper conclusion on impact of plant diversity.
Relation to calculating the vegetation diversity along with environmental parameters, in location specific data needs clarification / rectification.
R#21 – We agreed, basic information regarding the data collection of soil and vegetation sampling are now mentioned in detail as per suggestion. (L. 106-112, 114-116, 123-137, 142-148, 154-159, 191-193)

Further, the following points / comments need clarification / improvement.
Line no. 24-31: The abstract needs little improvement.
R#22 – Improved.

Line no. 32 & 100-104: The location can be provided with district / provinces / state, etc. in Pakistan.
R#23 – Required details provided in both the sections. (L. 11, 90-91)

Line no. 106: What are the growing seasons targeted? The time period of the study also be specified.
R#24 – We now added the months that represent the growing season in the area, which is from March to October. (L. 106)

Line no. 116-118: What is the deepness of soil sampling? Whether the soil has been collected for each stands / transect, please clarify.
R#25 – “Soil samples of 200-400grams were collected from three randomly selected transects (0-30cm depth) within each sampling stand of the studied vegetation area.” For more details, please see section: Environmental gradients. (L. 123-137)

Line no. 119-121: The duration, intervals, etc. of data obtained from the handheld weather station and position / place of the weather station for the study are not clear.
R#26 – We used a small remote weather station and recorded the data at transect level and then averaged it to stand level. (L. 142-144)

Line no. 177: Details of 244 species recorded may be supplemented.
R#27 – Two new supplementary tables have been with a summary of the environmental variables and the species composition based on the importance value index.

Line no. 182-186: The community may be specified as Trees, Shrubs, Grass, etc. and can be analysed properly. For example, the presence of *Indigofera* is shrub and *Cynodon* is grass and the landscape is shrubland or grassland. On the other hand, the presence of *Pinus* and *Cedrus* are clearly representing tree communities. Whether the landscape is having patches of

vegetation of trees / shrub / grass composition or uniform vegetation dominated by these group, need clarification.

R#28 – As per suggestion, the required details have been mentioned following the communities. For instance, the IHC community was primarily found in the lower mountainous ranges (1932.3-2338.4 m.a.s.l), where the dominating flora was a combination of shrub and herb species owing to the existence of a substantial herbaceous layer of *Cynodon dactylon*, which carpeted the landscape alongside *Indigofera heterantha* patches……shrubby associates. (L. 256-265)

Line no. 190-194: Any specific observation on *Cedrus, Pinus,* etc. Because, we could not find the all vegetation in all aspects, especially the studied tree species.

R#29 – Please see R#28 for the answer about this question. (L. 237-246, 264-266)

Discussion: Need revision in view of answering the above questions.

R#30 – We provided more details about how the variables can influence plant community. We cordially ask to check the lines (L. 356-358, 393-395, 404-412, 424-460), where we added information about the influence of other variables in the community structure.

The manuscript holds excellent statistical analysis. Although, basic collection data on vegetation assessment and related environmental parameters are missing, and can be addressed.

We cordially appreciate your positive words, however, we have considered and addressed each of your suggestion accordingly.
