# Supplementary material for: Environmental variables drive plant species composition and distribution in the moist temperate forests of Northwestern Himalaya, Pakistan
Source: PLoS One. 2022 Feb 24;17(2):e0260687. doi: 10.1371/journal.pone.0260687 (PMC8870539; doi:10.1371/journal.pone.0260687)
Supplement: Attachment — Submitted filename: Comments on the manuscript entitled.docx [file pone.0260687.s001.docx]

Comments on the manuscript entitled ‘Environmental variables drive plant species composition and distribution in the moist temperate forests of Northwestern Himalaya, Pakistan’.

The basic data collection on vegetation, soil, etc. and analysis are completely lacking.

Overall, the environmental observation of the study is very less (4 years, 2015-2018) to draw proper conclusion on impact of plant diversity.

Relation to calculating the vegetation diversity along with environmental parameters, in location specific data needs clarification / rectification.

Further, the following points / comments need clarification / improvement.

Line no. 24-31: The abstract needs little improvement.

Line no. 32 & 100-104: The location can be provided with district / provinces / state, etc. in Pakistan.

Line no. 106: What are the growing seasons targeted? The time period of the study also be specified.

Line no. 116-118: What is the deepness of soil sampling? Whether the soil has been collected for each stands / transect, please clarify.

Line no. 119-121: The duration, intervals, etc. of data obtained from the handheld weather station and position / place of the weather station for the study are not clear.

Line no. 177: Details of 244 species recorded may be supplemented.

Line no. 182-186: The community may be specified as Trees, Shrubs, Grass, etc. and can be analysed properly. For example, the presence of *Indigofera* is shrub and *Cynodon* is grass and the landscape is shrubland or grassland. On the other hand, the presence of *Pinus* and *Cedrus* are clearly representing tree communities. Whether the landscape is having patches of vegetation of trees / shrub / grass composition or uniform vegetation dominated by these group, need clarification.

Line no. 190-194: Any specific observation on *Cedrus, Pinus,* etc. Because, we could not find the all vegetation in all aspects, especially the studied tree species.

Discussion: Need revision in view of answering the above questions.

**The manuscript holds excellent statistical analysis. Although, basic collection data on vegetation assessment and related environmental parameters are missing, and can be addressed.**

**In view of above, the manuscript can be proposed as ‘Major Revision’.**
