# Supplementary material for: Environmental variables drive plant species composition and distribution in the moist temperate forests of Northwestern Himalaya, Pakistan
Source: PLoS One. 2022 Feb 24;17(2):e0260687. doi: 10.1371/journal.pone.0260687 (PMC8870539; doi:10.1371/journal.pone.0260687)
Supplement: Attachment — Submitted filename: Reviewers comment.doc [file pone.0260687.s002.doc]

The authors evaluate plant species composition and distribution furthermore, how and which environmental variables drive the plant communities of moist temperate forest, Himalaya, Pakistan. They sampled 30 sites and measured 21 environmental variables. Different multivariate analyses were used to identify potential plant communities and determine the relative importance of each environmental variable in the species composition and distribution. The findings have divided the temperate zone into four different plant communities, and a specific environmental variable explained each community. Plant species compositions varied significantly across plant communities and mainly six species contributed most to plant species composition dissimilarity. They found a significant difference in the four diversity indices between communities; plant community structure is twice more influenced by the spatial turnover of species than by the species loss.

The topic is timely, and such a kind of study, especially from the underrepresented areas, is essential and requires our attention. The authors have come up with clear questions and have analyzed them well. My main concern is with the methodology and discussion section; I see much basic information missing, especially concerning the data collection and study site; please look at each of the comments provided below. Discussion lacks proper explanation, key findings of different communities and predictor variables associated with them are interesting, but these points are not discussed well. Currently, the discussion is too general, but there is much potential to improve it. I also felt that your questions 2 and 4 explain the same points, so instead of keeping them as standalone objectives, it will be nice to combine them. In objective two results, all the four communities were found separately in clumps with apparent differences based on the environmental gradients, so I dint understand the need to again look into the variation of environmental variables among plant communities (iv). Lastly, instead of just reporting six species names, it will be good to have a table (primary or supplementary table), including all details of plant species in each community, their abundance, elevation range, etc.

**Here are my comments**

**L39-40:**  ii) each plant community has a specific set of environmental drivers & L41-42 iv) most of the environmental variables were significantly different between communities

I feel both of these sentences highlight the same points; my advice would be to combine both and write them as a single point.

**L90-91- and L94:** I am highlighting this point again; there is an overlap in your questions 2 and 4

**L106** What were the growing seasons? Please mention the seasons name

**L106** Was the same stand sampled for four years, meaning four times?

Please note that many important information on sampling design is missing; for example, how were the individuals counted, those falling precisely on the line? Furthermore, was the line horizontal or vertical? Was it stratified random sampling? Also, what was the distance between the two lines transect?

**L116** There is no clarity on how soil samples, Ph values were collected. Was it collected at a single location per stand, and what depth, slope, well-exposed sunlit areas or not?

Ph varies a lot, even at a short distance between north and south facing slopes, so it is better to provide all the sampling details.

**L121** At what distance was the weather station located from those 30 stands? The environmental variable varies across the elevation, and authors have also mentioned the importance of microclimates, so I am curious to know how many weather stations were installed.

The overall sampling design will become more apparent if the authors explain how the line transects and weather stations are distributed along the elevation gradient.

**Results:**

I feel that apart from the given figures, it will be good to have a table where the species names, elevation range, abundance in each community are mentioned. A comprehensive table with all these details may give a clearer picture of your findings. You may decide how much information you would want to share in the main manuscript and supplementary information.

**Fig 3:** Legend need to be rewritten, the author has mentioned four communities' names and four colors corresponding to each community, but the link between each color and community is missing. Also, what do the alphabet and various numbers indicate on the left side of the graph?

**L209:** What was the intention behind considering temperature and altitude both as the predictor variables? Because along the mountain gradient these variables are highly correlated.

**Discussion:** In the result, section author has emphasized their findings of four distinct communities and the environmental variables associated with each one of them. However, in the discussion, the authors have failed to discuss their unique findings in detail. I feel that the discussion section is too general and poorly explained. There is a lot of scopes to improve the discussion considering the amount of analysis carried out. By highlighting just temperature and altitude (which is definitely important from the climate change aspect), you may lose other valuable details of your study.
