# Supplementary material for: Environmental variables drive plant species composition and distribution in the moist temperate forests of Northwestern Himalaya, Pakistan
Source: PLoS One. 2022 Feb 24;17(2):e0260687. doi: 10.1371/journal.pone.0260687 (PMC8870539; doi:10.1371/journal.pone.0260687)
Supplement: Attachment — Submitted filename: Comments-PONE-D-21-11989.docx [file pone.0260687.s003.docx]

*General comments:*

In this research paper, the authors studied influence of environmental variables in shaping plant species composition and their distribution in the moist temperate forests of Northwestern Himalaya, Pakistan. Covering 30 sampling sites, they measured 21 environmental variables for four consecutive years. Generated data is analysed using different multivariate analyses in order to identify potential plant communities and influence of environmental variable in species composition, distribution and diversity patterns. Overall the present research is well-designed and presents interesting results of multivariate analysis in order to understand how the existence of micro-climates drive the structure and composition of plant species in studied area.

*Major issue:*

1. In Conclusions (line 46-48) author stated that overall, we showed that altitudinal gradients offer an important range of different environmental variables, highlighting the existence of micro-climates that drive the structure and composition of plant species in each micro-region. However there is no mention of elevation range of study area across the manuscript.
2. Kindly add some detailed information on sampling method such as: if quadrat method is opted during the study what was the size of these used quadrats. Furthermore it will be good for readers to understand the study if information regarding the number of sampling quadrates in each transect will be mentioned in the methodology section.
3. A detailed table describing four communities (**IHC; VIP; CPI; PCP)** needs to be added highlighting species composition, environmental variables, elevation range, dominant families etc.
4. Page 9 line 177 author mentioned “A total of 244 plant species were recorded 177 in moist temperate forest of Manoor valley, Himalaya, Pakistan”. Kindly add details of 244 plant species belonging to ……………genus and …….families respectively.

*Other comments:*

1. Kindly follow uniformity across the manuscript text to avoid confusion, such as spelling variations (in line 122 analyses and 123 analyzes) and such omissions needs to be checked throughout the manuscript.
2. Author has used the term Northwestern Himalaya (line 23); Himalaya (line 31): Northwestern Himalaya (line 102); Himalayan mountains (line 260) etc. thus it is suggested to use term Northwestern Himalaya to indicate study site throughout the manuscript.
3. For wider acceptability of the manuscript it is suggested to discuss similar studies carried out in other parts of Himalaya in discussion section.
