# Supplementary material for: Environmental variables drive plant species composition and distribution in the moist temperate forests of Northwestern Himalaya, Pakistan
Source: PLoS One. 2022 Feb 24;17(2):e0260687. doi: 10.1371/journal.pone.0260687 (PMC8870539; doi:10.1371/journal.pone.0260687)
Supplement: Attachment — Submitted filename: Response to reviewers.docx [file pone.0260687.s004.docx]

Manuscript ID # PONE-D-21-11989

Dear Editor,

We thank you and Reviewers for their constructive criticism and are pleased to resubmit a revised and improved version of our manuscript (ID # PONE-D-21-11989) entitled “”. We carefully responded to each comment in the revised version. In the response letter below, we have provided a detailed explanation of the changes made following the line numbers (see line numbers in track version).

Sincerely yours,

Inayat Ur Rahman, PhD

**Revision notes**

Reviewer’s comments black font; author answers in blue font; responses are numbered (R#1, R#2, etc. for reference)

**Reviewer(s)’ Comments to Author:**

**Reviewer #1**

*General comments:*

In this research paper, the authors studied influence of environmental variables in shaping plant species composition and their distribution in the moist temperate forests of Northwestern Himalaya, Pakistan. Covering 30 sampling sites, they measured 21 environmental variables for four consecutive years. Generated data is analysed using different multivariate analyses in order to identify potential plant communities and influence of environmental variable in species composition, distribution and diversity patterns. Overall the present research is well-designed and presents interesting results of multivariate analysis in order to understand how the existence of micro-climates drive the structure and composition of plant species in studied area.

R#1 – We really appreciate the positive words.

*Major issue:*

1. In Conclusions (line 46-48) author stated that overall, we showed that altitudinal gradients offer an important range of different environmental variables, highlighting the existence of micro-climates that drive the structure and composition of plant species in each micro-region. However there is no mention of elevation range of study area across the manuscript.

R#2 – We now added this information in the manuscript (L.14-15,93,256-265). The altitudinal range of our data sampling ranged from 1932 to 3168 m.a.s.l.

2. Kindly add some detailed information on sampling method such as: if quadrat method is opted during the study what was the size of these used quadrats. Furthermore it will be good for readers to understand the study if information regarding the number of sampling quadrates in each transect will be mentioned in the methodology section.

R#3 – Required information regarding the vegetation sampling has been added. “The surveyed stands (study area) were subdivided into 30 stands and three points randomly selected within each stand were sampled along 50 meters transect (total = 90 transects). The interval distance kept between the stands was 200 meters and 100 meters between transects.” (L. 106-112)

3. A detailed table describing four communities (**IHC; VIP; CPI; PCP)** needs to be added highlighting species composition, environmental variables, elevation range, dominant families etc.

R#4 – We now added two new supplementary tables (Table S1 and S2) describing the communities. One table describes environmental variables and the other table the species composition for each plant community.

4. Page 9 line 177 author mentioned “A total of 244 plant species were recorded 177 in moist temperate forest of Manoor valley, Himalaya, Pakistan”. Kindly add details of 244 plant species belonging to ……………genus and …….families respectively.

R#5 – As per suggestion, the required details has been added i.e., 194 genera and 74 families. (L. 246)

*Other comments:*

1. Kindly follow uniformity across the manuscript text to avoid confusion, such as spelling variations (in line 122 analyses and 123 analyzes) and such omissions needs to be checked throughout the manuscript.

R#6 – Done. Thank you. (L. 15, 138, 145, 148, 152, 239, 322)

2. Author has used the term Northwestern Himalaya (line 23); Himalaya (line 31): Northwestern Himalaya (line 102); Himalayan mountains (line 260) etc. thus it is suggested to use term Northwestern Himalaya to indicate study site throughout the manuscript.

R#7 – We changed to Northwestern Himalaya in the places that we are talking about the specific region of Himalayas. Other parts are related to the Himalayas in a general perspective and then we can change it. We appreciate your understanding. (L. 10, 12, 40, 71, 80, 92, 95 and onwards)

3. For wider acceptability of the manuscript it is suggested to discuss similar studies carried out in other parts of Himalaya in discussion section.

R#8 – We added more citations about studies conducted in the Himalayan mountains. We have now discussed it in detail. We now also added more citations about plant community studies in the Himalayas. (L. 356-358, 393-395, 404-412, 424-460)

**Reviewer #2**

The topic is timely, and such a kind of study, especially from the underrepresented areas, is essential and requires our attention. The authors have come up with clear questions and have analyzed them well.

R#9 – We really appreciate your positive words.

My main concern is with the methodology and discussion section; I see much basic information missing, especially concerning the data collection and study site; please look at each of the comments provided below. Discussion lacks proper explanation, key findings of different communities and predictor variables associated with them are interesting, but these points are not discussed well. Currently, the discussion is too general, but there is much potential to improve it. I also felt that your questions 2 and 4 explain the same points, so instead of keeping them as standalone objectives, it will be nice to combine them. In objective two results, all the four communities were found separately in clumps with apparent differences based on the environmental gradients, so I dint understand the need to again look into the variation of environmental variables among plant communities (iv). Lastly, instead of just reporting six species names, it will be good to have a table (primary or supplementary table), including all details of plant species in each community, their abundance, elevation range, etc.

R#10 – We now improved our manuscript and cordially ask you to check our responses for each question raised. Overall, we added more information in methods (L. 93, 106-112, 114-116, 123-137, 142-144, 146-148, 150-151, 154-159, 191-193) and discussion (L. 356-358, 393-395, 404-412, 424-460) sections. Following reviewer’s very important suggestion, two new supplementary tables with a summary of the environmental variables and the species composition based on the importance value were also added.

**L39-40:**  ii) each plant community has a specific set of environmental drivers & L41-42 iv) most of the environmental variables were significantly different between communities. I feel both of these sentences highlight the same points; my advice would be to combine both and write them as a single point.

R#11 – Thank you for your suggestion. We agreed and combined accordingly. (L. 22, 84, 366)

**L90-91- and L94:** I am highlighting this point again; there is an overlap in your questions 2 and 4

R#12 – Please see R#11 for the answer about this question. (L. 22, 84, 366)

**L106** What were the growing seasons? Please mention the seasons name

R#13 – We now added the months that represent the growing seasons in the area, which were from March to October. (L. 106)

**L106** Was the same stand sampled for four years, meaning four times? Please note that many important information on sampling design is missing; for example, how were the individuals counted, those falling precisely on the line? Furthermore, was the line horizontal or vertical? Was it stratified random sampling? Also, what was the distance between the two lines transect?

R#14 – Yes. We repeated the stands over years, but not the same transects inside each stand. We now added more information about it the text. The surveyed stands (study area) were subdivided into 30 stands and three points randomly selected within each stand were sampled along 50 meters transect (total = 90 transects). The interval distance kept between the stands was 200 meters and 100 meters between transects. The individuals of plant species falling precisely on the line were noted. (L. 106-112)

**L116** There is no clarity on how soil samples, Ph values were collected. Was it collected at a single location per stand, and what depth, slope, well-exposed sunlit areas or not?

Ph varies a lot, even at a short distance between north and south facing slopes, so it is better to provide all the sampling details.

R#15 – Basic details regarding the data collection of vegetation and soil sampling are now mentioned in Vegetation sampling (L. 108-112, 114-116), and Environmental gradients (L. 123-137, 142-144) respectively. A supplementary table S2 has also been included to show the variability of environmental gradients among the four plant communities.

**L121** At what distance was the weather station located from those 30 stands? The environmental variable varies across the elevation, and authors have also mentioned the importance of microclimates, so I am curious to know how many weather stations were installed.

The overall sampling design will become more apparent if the authors explain how the line transects and weather stations are distributed along the elevation gradient.

R#16 – We used a small remote weather station and recorded the data at transect level and then averaged it to stand level. Required details regarding vegetation sampling using line transect are incorporated into the Vegetation sampling section (M & M).

**Results:**

I feel that apart from the given figures, it will be good to have a table where the species names, elevation range, abundance in each community are mentioned. A comprehensive table with all these details may give a clearer picture of your findings. You may decide how much information you would want to share in the main manuscript and supplementary information.

R#17 – Thank you for your comment. We now added two supplementary tables about plant species composition and environmental variables values in each community. Since we already have many tables and figures in the manuscript, and these other tables will be so long, we are adding them as supplementary tables (S1 and S2).

**Fig 3:** Legend need to be rewritten, the author has mentioned four communities' names and four colors corresponding to each community, but the link between each color and community is missing. Also, what do the alphabet and various numbers indicate on the left side of the graph?

R#18 – We now updated our legend. Numbers and letter are the stands.

“Figure 3. Clustering method using two way indicator species analysis (TWINSPAN) indicating four different plant communities. IHC (red triangle): *Indigofera heterantha-Heracleum candicans-Cynodon dactylon,* VIP (blue circle): *Viburnum grandiflorum-Indigofera heterantha-Pinus wallichiana,* CPI (green square): *Cedrus deodara-Pinus wallichiana-Isodon rugosus* and PCP (yellow diamond): *Pinus wallichiana-Cedrus deodara- Parrotiopsis jacquemontiana.* Letters associated to numbers at the end of each branch of the dendrogram represent the stands evaluated.

**L209:** What was the intention behind considering temperature and altitude both as the predictor variables? Because along the mountain gradient these variables are highly correlated.

R#19 – In this specific analysis, we did not consider any kind of multicollinearity. We plot all variables to see the overall relationship between them and communities. However, if you check the partial CCA and partitioning analyses, you will see that we removed the colinear variables. Therefore, in the first analysis (NMDS, PCA) we show the overall relationship of all variables with each community, while in the second analysis, we show which are the variables that most explain the patterns found in our study.

**Discussion:** In the result, section author has emphasized their findings of four distinct communities and the environmental variables associated with each one of them. However, in the discussion, the authors have failed to discuss their unique findings in detail. I feel that the discussion section is too general and poorly explained. There is a lot of scopes to improve the discussion considering the amount of analysis carried out. By highlighting just temperature and altitude (which is definitely important from the climate change aspect), you may lose other valuable details of your study.

R#20 – We totally agree. In discussion, we provided more details about how these other variables can influence plant community. We believe that discussing each variable and its relationship to the community will extend a lot the manuscript, turning it tiring for readers. In this context, we believe that the general patterns and discussion of the main variables should be the best approach. We appreciate your comment, and we cordially ask to check the lines mentioned, where we added information about the influence of other variables in the community structure. Some important edaphic variables like pH were considered and discussed accordingly. (L. 356-358, 393-395, 404-412, 424-460)

**Reviewer #3**

Comments on the manuscript entitled ‘Environmental variables drive plant species composition and distribution in the moist temperate forests of Northwestern Himalaya, Pakistan’.

The basic data collection on vegetation, soil, etc. and analysis are completely lacking.

Overall, the environmental observation of the study is very less (4 years, 2015-2018) to draw proper conclusion on impact of plant diversity.

Relation to calculating the vegetation diversity along with environmental parameters, in location specific data needs clarification / rectification.

R#21 – We agreed, basic information regarding the data collection of soil and vegetation sampling are now mentioned in detail as per suggestion. (L. 106-112, 114-116, 123-137, 142-148, 154-159, 191-193)

Further, the following points / comments need clarification / improvement.

Line no. 24-31: The abstract needs little improvement.

R#22 – Improved.

Line no. 32 & 100-104: The location can be provided with district / provinces / state, etc. in Pakistan.

R#23 – Required details provided in both the sections. (L. 11, 90-91)

Line no. 106: What are the growing seasons targeted? The time period of the study also be specified.

R#24 – We now added the months that represent the growing season in the area, which is from March to October. (L. 106)

Line no. 116-118: What is the deepness of soil sampling? Whether the soil has been collected for each stands / transect, please clarify.

R#25 – “Soil samples of 200-400grams were collected from three randomly selected transects (0-30cm depth) within each sampling stand of the studied vegetation area.” For more details, please see section: Environmental gradients. (L. 123-137)

Line no. 119-121: The duration, intervals, etc. of data obtained from the handheld weather station and position / place of the weather station for the study are not clear.

R#26 – We used a small remote weather station and recorded the data at transect level and then averaged it to stand level. (L. 142-144)

Line no. 177: Details of 244 species recorded may be supplemented.

R#27 – Two new supplementary tables have been with a summary of the environmental variables and the species composition based on the importance value index.

Line no. 182-186: The community may be specified as Trees, Shrubs, Grass, etc. and can be analysed properly. For example, the presence of *Indigofera* is shrub and *Cynodon* is grass and the landscape is shrubland or grassland. On the other hand, the presence of *Pinus* and *Cedrus* are clearly representing tree communities. Whether the landscape is having patches of vegetation of trees / shrub / grass composition or uniform vegetation dominated by these group, need clarification.

R#28 – As per suggestion, the required details have been mentioned following the communities. For instance, the IHC community was primarily found in the lower mountainous ranges (1932.3-2338.4 m.a.s.l), where the dominating flora was a combination of shrub and herb species owing to the existence of a substantial herbaceous layer of *Cynodon dactylon*, which carpeted the landscape alongside *Indigofera heterantha* patches……shrubby associates. (L. 256-265)

Line no. 190-194: Any specific observation on *Cedrus, Pinus,* etc. Because, we could not find the all vegetation in all aspects, especially the studied tree species.

R#29 – Please see R#28 for the answer about this question. (L. 237-246, 264-266)

Discussion: Need revision in view of answering the above questions.

R#30 – We provided more details about how the variables can influence plant community. We cordially ask to check the lines (L. 356-358, 393-395, 404-412, 424-460), where we added information about the influence of other variables in the community structure.

**The manuscript holds excellent statistical analysis. Although, basic collection data on vegetation assessment and related environmental parameters are missing, and can be addressed.**

We cordially appreciate your positive words, however, we have considered and addressed each of your suggestion accordingly.
