# Supplementary material for: Environmental variables drive plant species composition and distribution in the moist temperate forests of Northwestern Himalaya, Pakistan
Source: PLoS One. 2022 Feb 24;17(2):e0260687. doi: 10.1371/journal.pone.0260687 (PMC8870539; doi:10.1371/journal.pone.0260687)
Supplement: Attachment — Submitted filename: Response to reviewers.docx [file pone.0260687.s006.docx]

Manuscript ID # PONE-D-21-11989

Dear Editor,

We thank you and Reviewers for their constructive criticism and are pleased to resubmit a revised and improved version of our manuscript (ID # PONE-D-21-11989) entitled “**Environmental variables drive plant species composition and distribution in the moist temperate forests of Northwestern Himalaya, Pakistan**”. We carefully responded to each comment in the revised version. In the response letter below, the only point left has been explained in detail, while all the rest were positively considered and incorporated (see in track version).

Sincerely yours,

Inayat Ur Rahman, PhD

**Revision notes**

Reviewers and Editor’s comments black font; author answers in blue font.

**Reviewer(s)’ Comments to Author:**

**Editor’s Comments to Author:**

**Comment:** The authors have studied the variation of plant communities along environmental gradients in the Moist North Western Himalaya, and linked the plant communities with various environmental variables. The article is well written with clarity in introduction, methods (including data analysis), results and discussion. In the first round of review, three independent expert reviewers provided valuable comments which were mostly addressed by the authors. The revised version was sent to all the three same previous reviewers and all of them have positively commented on the MS. I have once again thoroughly evaluated the MS and provided some editorial comments. I suggest the authors to look into the comments (as sticky notes in the attached pdf manuscript files) and address them critically. I also suggest the authors to provide some more details in the sampling design and methodology section as pointed by reviewer 1. Once all these minor issues are resolved, the MS may be considered for publication. I request the authors to revise the MS quickly and submit the same. The MS may not be sent for further external review but will be evaluated by the academic editor before rendering the final decision. Looking forward to the revised MS at an early date.

**Response:** We really appreciate the reviewers for very constructive criticism and acceptance of our work. We are thankful to the Editor for highlighting such important points, which made the revised version much better. We have considered all the raised points and corrected the manuscript accordingly.

**Comment:** The Editor highlighted suggestions in the pdf file.

**Response:** We are thankful to the Editor for highlighting such important points, which made the revised version much better. We have considered all the raised points and corrected the manuscript accordingly. Each Table and Figure legend/caption were corrected with proper and complete details. The analysis and the linked results that were highlighted to be deleted have been done as suggested. We cordially ask you to check the track version for corrections.

**Comment:** Sorenson dissimilarly index (βsor) is an incidence based index which can be partitioned into turnover (βsim) and nestedness (βnes) components. Simpson is a different index. Again why authors have used only incidence based index? Why not abundance based index because abundance-based β-diversity can be estimated as Bray-Curtis dissimilarity index (dBC) and then partitioned into balanced variation (dBC-bal) and abundance gradient components (dBC-gra). Since authors have abundance data, both the approaches could give better result. Please see the following article for details:

Sharma, K., B.K. Acharya, G. Sharma, D. Valente, M.R Pasimeni, I. Petrosillo, and T. Selvan. 2020. Land use effect on butterfly alpha and beta diversity in the Eastern Himalaya, India. Ecological Indicators 110: 105605.

**Response:** We agree that when working with abundance, abundance-based B-diversity estimated as dBC is the best option. However, in all our analyses we used the importance value (IV), as described in the Vegetation Sampling and plant identification subtopic in Material and Methods, to standardize our analyses over the manuscript. IV takes in account relative frequency, relative density, and relative dominance. In this way, we chose to use the Bsor index based on the incidence to decrease potential confounding effects of using IV when calculating dBC. In few words, we used a more conservative Beta diversity analysis. For these reasons, we cordially ask you to keep the incidence-based Beta diversity analysis in our study.
